# Seasonal plasticity of cognition and related biological measures in adults with and without Alzheimer disease: Analysis of multiple cohorts

**DOI:** 10.1371/journal.pmed.1002647

**Published:** 2018-09-04

**Authors:** Andrew S. P. Lim, Chris Gaiteri, Lei Yu, Shahmir Sohail, Walter Swardfager, Shinya Tasaki, Julie A. Schneider, Claire Paquet, Donald T. Stuss, Mario Masellis, Sandra E. Black, Jacques Hugon, Aron S. Buchman, Lisa L. Barnes, David A. Bennett, Philip L. De Jager

**Affiliations:** 1 Division of Neurology, Department of Medicine, Hurvitz Brain Sciences Program, Sunnybrook Health Sciences Centre, University of Toronto, Toronto, Ontario, Canada; 2 Rush Alzheimer Disease Center, Rush University Medical Center, Chicago, Illinois, United States of America; 3 Department of Neurological Sciences, Rush University, Chicago, Illinois, United States of America; 4 Department of Pharmacology and Toxicology, University of Toronto, Toronto, Ontario, Canada; 5 Centre de Neurologie Cognitive, Hôpitaux Saint-Louis Lariboisière Fernand-Widal, Assistance Publique–Hôpitaux de Paris, University of Paris Diderot, Paris, France; 6 Inserm U942, Paris, France; 7 Department of Behavioral Sciences, Rush University Medical Center, Chicago, Illinois, United States of America; 8 Center for Translational & Computational Neuroimmunology, Department of Neurology, Columbia University Medical Center, New York, New York, United States of America; Mayo Clinic, UNITED STATES

## Abstract

**Background:**

There are few data concerning the association between season and cognition and its neurobiological correlates in older persons—effects with important translational and therapeutic implications for the diagnosis and treatment of Alzheimer disease (AD). We aimed to measure these effects.

**Methods and findings:**

We analyzed data from 3,353 participants from 3 observational community-based cohort studies of older persons (the Rush Memory and Aging Project [MAP], the Religious Orders Study [ROS], and the Minority Aging Research Study [MARS]) and 2 observational memory-clinic-based cohort studies (Centre de Neurologie Cognitive [CNC] study at Lariboisière Hospital and the Sunnybrook Dementia Study [SDS]). We performed neuropsychological testing and, in subsets of participants, evaluated cerebrospinal fluid AD biomarkers, standardized structured autopsy measures, and/or prefrontal cortex gene expression by RNA sequencing. We examined the association between season and these variables using nested multiple linear and logistic regression models. There was a robust association between season and cognition that was replicated in multiple cohorts (amplitude = 0.14 SD [a measure of the magnitude of seasonal variation relative to overall variability; 95% CI 0.07–0.23], *p =* 0.007, in the combined MAP, ROS, and MARS cohorts; amplitude = 0.50 SD [95% CI 0.07–0.66], *p =* 0.017, in the SDS cohort). Average composite global cognitive function was higher in the summer and fall compared to winter and spring, with the difference equivalent in cognitive effect to 4.8 years’ difference in age (95% CI 2.1–8.4, *p =* 0.002). Further, the odds of meeting criteria for mild cognitive impairment or dementia were higher in the winter and spring (odds ratio 1.31 [95% CI 1.10–1.57], *p =* 0.003). These results were robust against multiple potential confounders including depressive symptoms, sleep, physical activity, and thyroid status and persisted in cases with AD pathology. Moreover, season had a marked effect on cerebrospinal fluid Aβ 42 level (amplitude 0.30 SD [95% CI 0.10–0.64], *p =* 0.003), which peaked in the summer, and on the brain expression of 4 cognition-associated modules of co-expressed genes (m6: amplitude = 0.44 SD [95% CI 0.21–0.65], *p =* 0.0021; m13: amplitude = 0.46 SD [95% CI 0.27–0.76], *p =* 0.0009; m109: amplitude = 0.43 SD [95% CI 0.24–0.67], *p =* 0.0021; and m122: amplitude 0.46 SD [95% CI 0.20–0.71], *p =* 0.0012), which were in phase or anti-phase to the rhythms of cognition and which were in turn associated with binding sites for several seasonally rhythmic transcription factors including BCL11A, CTCF, EGR1, MEF2C, and THAP1. Limitations include the evaluation of each participant or sample once per annual cycle, reliance on self-report for measurement of environmental and behavioral factors, and potentially limited generalizability to individuals in equatorial regions or in the southern hemisphere.

**Conclusions:**

Season has a clinically significant association with cognition and its neurobiological correlates in older adults with and without AD pathology. There may be value in increasing dementia-related clinical resources in the winter and early spring, when symptoms are likely to be most pronounced. Moreover, the persistence of robust seasonal plasticity in cognition and its neurobiological correlates, even in the context of concomitant AD pathology, suggests that targeting environmental or behavioral drivers of seasonal cognitive plasticity, or the key transcription factors and genes identified in this study as potentially mediating these effects, may allow us to substantially improve cognition in adults with and without AD.

## Abstract 2

Andrew Lim and colleagues analyze data from five cohort studies to examine whether seasonal fluctuations in cognition are accompanied by fluctuations in Alzheimer’s disease-related proteins and gene expression patterns in the brain.

## Author summary

**Why was this study done?:**

- Season affects many aspects of human biology including human brain biology.
- Several studies suggest that season may be associated with cognitive function in some populations of younger adults, but data from older adults is lacking and little is known about the underlying mechanisms.

**What did the researchers do and find?:**

- We studied 3,353 older adults in the United States, Canada, and France.
- We tested their thinking and concentration, measured Alzheimer-disease-related proteins in their spinal fluid, performed autopsies on those who died, and measured brain gene expression.
- We found that older adults with and without dementia have better thinking and concentration in the late summer and early fall than in the winter and spring.
- This was accompanied by seasonal rhythms in Alzheimer-disease-related proteins in the spinal fluid, and in the expression of specific genes in the brain, giving us a window into the underlying mechanisms.

**What do these findings mean?:**

- There may be benefit to increasing dementia diagnosis and care resources in the winter and early spring, when individuals’ thinking and concentration are at their worst.
- By shedding light on the mechanisms underlying the seasonal improvement in cognition in the summer, these findings also open the door to new avenues of treatment for Alzheimer disease.

## Introduction

Seasonal rhythms modulate several aspects of human behavior and physiology including brain functions such as mood in seasonal affective disorder [1], symptom onset in schizophrenia [2], and functional MRI (fMRI) brain responses to cognitive tasks [3]. Several studies suggest that season may modulate cognition in younger adults [4,5], although this is not a universal finding [3,6,7], and data from older adults are lacking.

Demonstration of seasonal modulation of cognition and its neural substrates in older adults would have important clinical and translational implications. It would suggest that Alzheimer disease (AD) might be a seasonal illness, and that dementia care resources should be targeted to seasons of peak need, both to identify those at the earliest stages of disease, and to support patients when they are most vulnerable. Seasonal modulation may account for the observation that some individuals with mild cognitive impairment (MCI) subsequently revert to normal cognition, and may be an important source of diagnostic misclassification both in clinical trials and in clinical practice. Most importantly, it may shed light on mechanisms of cognitive plasticity that might be leveraged to improve cognition in patients with AD.

We recently demonstrated seasonal rhythms in human neocortical gene expression in older adults with and without AD [8], In the present study of 3,353 older adults with and without AD in the United States, France, and Canada, we tested the hypotheses that season has a significant association with cognition, the odds of being diagnosed with MCI or dementia, cerebrospinal fluid (CSF) AD biomarkers, and the expression of cognition-associated modules of co-expressed genes in the human brain.

## Methods

### Study design and participants

We analyzed cross-sectional data at multiple time points from participants in 3 prospective observational community-based cohort studies of older persons (the Rush Memory and Aging Project [MAP], Religious Orders Study [ROS], and Minority Aging Research Study [MARS]) and 2 prospective observational clinic-based case series (the Centre de Neurologie Cognitive [CNC] study, at Lariboisière Hospital, University of Paris Diderot, and Assistance Publique–Hôpitaux de Paris, and the Sunnybrook Dementia Study [SDS]).

Unless otherwise indicated, all analyses were planned and performed in March 2018. These analyses were not planned prior to data collection. The analyses were performed following an analysis of seasonal rhythms of gene expression and epigenetic modification in the ROS and MAP cohorts, which has been reported elsewhere, and were partially driven by these earlier results [8]. Moreover, we had previously analyzed the cognitive data in relation to sleep [9]. The analyses were otherwise conceived of prior to seeing data from the MARS, SDS, and CNC cohorts. The analysis incorporating thyroid endocrine status (see below) was performed in revision in June of 2018 at the request of a reviewer. We related measures of cognition and clinical diagnoses of dementia to date of cognitive evaluation, CSF biomarkers of AD to date of lumbar puncture, and postmortem neocortical RNA sequencing (RNA-Seq) data to date of death relative to the calendar year.

Characteristics of the study participants are given in Table 1. The ROS, MAP, and MARS studies are prospective observational cohort studies of the risk factors for common chronic diseases of aging. They share nearly identical protocols. The ROS is a longitudinal study of aging in Catholic brothers, nuns, and priests from across the US [10]. The MAP is a community-based study of aging in the greater Chicago area that enrolls participants with diverse backgrounds and socioeconomic status from continuous care retirement communities throughout northwestern Illinois, as well as from individual homes across the Chicago metropolitan area [10]. Both the ROS and MAP cohorts consist primarily of individuals of self-reported European descent (93%). The MARS is a community-based study of older adults of self-reported African descent in the greater Chicago area [11]. Participants in all 3 studies are free of known dementia at study enrollment. All participants in the ROS and MAP cohorts and a subset of participants in the MARS cohort agree to brain donation upon death. For our primary analyses, we included all ROS, MAP, and MARS participants with complete cognitive testing (see below) and without cognitive impairment at the time of their first cognitive assessment. Of the 4,004 ROS, MAP, and MARS participants enrolled between January 1994 and May 2017, 3,924 completed the entire cognitive battery at their baseline assessment, 2,761 of whom were without cognitive impairment. Cognitive, demographic, and clinical data from these 2,761 participants were included in our primary analyses. In addition, structured autopsies were performed on 1,410 decedents, and RNA-Seq data from the dorsolateral prefrontal cortex (DLPFC) were obtained from 507 of these.

**Table 1:** Characteristics of the study population.

Characteristic	ROS/MAP baseline	MARS baseline	ROS/MAP/MARS				SDS	CNC
			Baseline	Last available	Deceased	Deceased with RNA-Seq		
*n*	2,234	527	2,761	2,761	1,410	507	271	321
Age at assessment (years)	77.4 (71.2, 82.3)	71.5 (68.6, 76.0)	76.0 (70.2, 81.4)	83.5 (77.5, 88.9)	88.0 (83.4, 92.3)	87.7 (83.5, 91.8)	72.1 (63.9, 78.9)	68.6 (62.8, 75.0)
Age at death (years)	NA	NA	NA	NA	89.2 (84.6, 93.4)	88.8 (84.5, 92.7)	NA	NA
Female sex	1,663 (74.4%)	418 (79.3%)	2,081 (75.4%)	2,081 (75.4%)	939 (66.6%)	314 (61.9%)	132 (48.7%)	182 (56.7%)
European descent	2,103 (94.1%)	0 (0.0%)	2,103 (76.2%)	2,103 (76.2%)	1,349 (95.7%)	502 (99.0%)	NA	NA
Years of education	16 (14, 19)	14 (12, 16)	16 (13, 18)	16 (13, 18)	16 (13, 18)	16 (14, 18)	13 (12, 16)	NA
MMSE	29 (28, 30)	29 (27, 29)	29 (28, 30)	28 (26, 29)	25 (17, 28)	26 (19, 28)	23 (20, 26)	24 (20, 27)
MCI	0 (0%)	0 (0%)	0 (0%)	457 (16.6%)	366 (26.0%)	145 (28.6%)	0 (0%)	56 (17.4%)
Clinical dementia	0 (0%)	0 (0%)	0 (0%)	356 (12.9%)	532 (37.7%)	186 (36.7%)	271 (100%)	176 (54.8%)
Pathological AD	NA	NA	NA	NA	901 (63.9%)	295 (58.2%)	NA	NA

Data are given as *n* (percent) or median (IQR).

AD, Alzheimer disease; CNC, Centre de Neurologie Cognitive; MAP, Rush Memory and Aging Project; MARS, Minority Aging Research Study; MCI, mild cognitive impairment; MMSE, Mini-Mental State Examination; NA, not applicable; RNA-Seq, RNA sequencing; ROS, Religious Orders Study; SDS, Sunnybrook Dementia Study.

The SDS (ClinicalTrials.gov NCT01800214) is an observational cohort study of consecutively collected cases (1992–2014) from a tertiary care clinic in Toronto, Canada [12]. For this study, we included participants clinically classified as having AD at their baseline assessment (see criteria below), and in whom Dementia Rating Scale (DRS) scores were available. Of 1,143 participants enrolled between 1992 and 2014, 285 had a primary clinical diagnosis of AD, of whom 271 had DRS scores at the time of baseline assessment. Clinical, demographic, and psychometric data from these 271 participants were included in this study. The CNC cohort consists of patients who attended a clinical and research memory clinic specializing in the care of patients with cognitive disorders, the memory clinic at the Lariboisière Hospital, between July 2, 2008, and June 15, 2017, who had undergone CSF collection with measurement of amyloid and tau, whose entire clinical work-up was performed at the Lariboisière Hospital, and for whom a final clinical diagnosis was available. Of 2,298 patients assessed between July 2008 and June 2017, the vast majority had had a portion of their clinical work-up completed at an outside institution and were excluded from these analyses. Of the remaining 424 participants with a final clinical diagnosis, 321 also had a lumbar puncture with quantification of Aβ 40, Aβ 42, tau, and phospho-tau. Psychometric, clinical, demographic, and CSF data from these 321 participants were included in this study.

### Statement of ethics approval

The ROS, MAP, and MARS were approved by the Institutional Review Board of Rush University Medical Center, the SDS was approved by the Research Ethics Board of Sunnybrook Research Institute, and the CNC study was approved by the Institutional Ethics Committee of University of Paris Diderot. All studies were conducted in accordance with the latest version of the Declaration of Helsinki, and all participants provided written informed consent. In addition, all ROS and MAP participants, and some MARS participants, signed an Anatomical Gift Act for organ donation.

### Assessment of cognition and diagnosis of dementia

Participants in the ROS, MAP, and MARS cohorts underwent annual uniform structured cognitive evaluations consisting of a battery of 19 cognitive tests spanning 5 domains (episodic memory [logical memory Ia, logical memory IIa, immediate story recall, delayed story recall, word list memory, word list recall, word list recognition], semantic memory [Boston naming test, category fluency, national adult reading test], working memory [digit span forward, digit span backward, digit ordering], perceptual speed [symbol digit modalities test, number comparison, Stroop word reading, Stroop color naming], and visuospatial ability [judgment of line orientation, standard progressive matrices]). As described previously [13], a composite measure for each domain was created by converting each test within each domain to a *z*-score and averaging the *z*-scores. The 5 domain composite measures were then averaged to create a composite global cognitive score scaled such that 0 represents the mean score of all participants at baseline, positive scores indicate better performance, and 1 unit represents approximately 1 standard deviation of performance. Participants were classified as having a clinical diagnosis of dementia by National Institute of Neurological and Communicative Disorders and Stroke–Alzheimer’s Disease and Related Disorders Association criteria [14]. Persons with cognitive impairment by neuropsychological testing but without a clinical diagnosis of dementia were classified as having MCI. Participants without dementia or MCI were classified as having no cognitive impairment. Date of cognitive assessment was recorded at the time of cognitive assessment.

Patients in the SDS cohort underwent a comprehensive battery of 17 tests as described previously [15]. Of these, data from the DRS [16], the California Verbal Learning Test [17], digit span forward and backward [18], the Digit Symbol Substitution Test [19], the Wisconsin Card Sorting Test [20], semantic fluency for animals [21,22], and the Benton Line Orientation Test [23] were included in these analyses. AD diagnoses were made by consensus of 2 independent clinicians according to prevailing clinical consensus criteria [24]. Date of cognitive assessment was recorded at the time of cognitive assessment.

Patients in the CNC cohort underwent routine clinical, neurological, and neuropsychological evaluations, and brain imaging as clinically indicated. On the basis of all available data, including CSF biomarkers, and in accordance with National Institute on Aging (NIA)–Alzheimer’s Association criteria [24], patients were classified into 2 groups: AD and non-AD. Complex cases were discussed, and diagnoses were made by a multidisciplinary team of neurologists, geriatricians, and neuropsychologists. Non-AD participants included participants with cognitive disorders other than AD including frontotemporal dementia, Lewy body disease, Parkinson disease, Creutzfeldt-Jakob disease, and non-degenerative dementia (including vascular dementia, alcohol-related dementia, normal pressure hydrocephalus, infectious disease, and psychiatric disorders among others).

### Assessment of clinical covariates

In the ROS, MAP, and MARS cohorts, we computed age at the time of cognitive assessment from the self-reported date of birth and the date of assessment. We computed age at death from the self-reported date of birth and the date of death. We recorded sex and self-reported race at the time of the baseline interview. Depressive symptoms were assessed on the date of cognitive evaluation with a 10-item version of the Center for Epidemiologic Studies–Depression (CES-D) scale [25]. Clock time of cognitive testing was recorded at the time of testing. Times were binned into hours, and considered as a categorical variable. For sleep duration, MAP and ROS participants were asked on the date of cognitive evaluation to report how many hours they usually slept at night over the preceding month. For physical activity, MAP and ROS participants were asked on the date of cognitive evaluation to report the number of hours per week spent in 5 categories of activities: walking, gardening or yard work, calisthenics or general exercise, bicycle riding, and swimming or water exercises [26]. In a subset of ROS, MAP, and MARS participants, serum thyroid stimulating hormone (TSH) levels were assessed by immunoassay by Quest Diagnostics (Madison, NJ, US).

In the SDS cohort, age, sex, and years of education were extracted from clinical records.

In the CNC cohort, age and sex were extracted from clinical records.

### Evaluation of CSF

In the CNC cohort, lumbar punctures were performed on fasting patients within 1 month following their clinical diagnosis, usually between 09:00 and 12:00. Within 4 hours of collection, samples were centrifuged at 1,000*g* for 10 minutes at 4 °C, aliquoted into 0.5-ml polypropylene tubes, and stored at −80 °C. Subsequently, CSF Aβ 40, Aβ 42, total tau, and phospho-tau were measured using a commercially available sandwich enzyme-linked immunosorbent assay (INNOTEST, Fujirebio Europe, Gent, Belgium) according to the manufacturer’s instructions. Date of lumbar puncture was recorded at the time of lumbar puncture.

### Neuropathological evaluation

Neuropathological evaluation was performed in decedents from the ROS, MAP, and MARS cohorts. AD pathology was quantified as described previously [27,28]. Neurofibrillary tangles, diffuse plaques, and neuritic plaques were visualized by Bielschowsky silver staining in sections from the frontal, temporal, parietal, and entorhinal cortices and the hippocampus. For a categorical pathological diagnosis of AD, cases were classified as no AD, low likelihood AD, intermediate likelihood AD, or high likelihood AD based on the NIA-Reagan criteria [29]; a participant was considered to have a pathological diagnosis of AD if their NIA-Reagan classification was “intermediate likelihood” or “high likelihood.” To generate a composite continuous measure of the burden of AD pathology, neurofibrillary tangles, diffuse plaques, and neuritic plaques were counted in the regions above, the raw counts were divided by the standard deviation of the same index for that region across the entire cohort, and the scaled scores were averaged as described previously [28]. In addition to the above, the percent area occupied by amyloid beta was quantified as previously described [30]. Briefly, after immersion fixation in paraformaldehyde, tissue blocks from 8 brain regions (mid-frontal cortex, premotor cortex, inferior temporal cortex, angular gyrus, calcarine cortex, anterior cingulate cortex, entorhinal cortex, and hippocampus CA1/subiculum) were embedded in paraffin, sectioned, and incubated with antibodies to amyloid beta (monoclonal mouse anti–amyloid beta clone 10D5; Elan Pharmaceuticals, San Francisco, CA, US) followed by development with diaminobenzidine and nickel. The mean percent area occupied by amyloid beta–immunopositive material was quantified in each of the 8 brain regions in each individual using a systematic random sampling approach and a multistage computational image analysis protocol as previously described [30]. These values were averaged across regions to generate a summary measure of amyloid load for each individual.

To quantify Lewy body pathology, 6-μm paraffin-embedded sections from the cingulate, entorhinal, midfrontal, middle temporal, and inferior parietal cortices and the substantia nigra were immunostained with antibodies to alpha synuclein (pSyn-64, 1:20,000; Wako Chemicals USA; Richmond, VA, US). The distribution of Lewy bodies was graded on a semi-quantitative scale (0 = none, 1 = brainstem or limbic predominant, 2 = neocortical) according to a modified version of published criteria [31].

Macroscopic cerebral infarcts were identified by visual inspection of 1-cm coronal slabs and confirmed by histological review as previously described [32]. Microscopic infarcts were quantified in a minimum of 9 regions (6 cortical and 3 subcortical) as previously described [33].

### Evaluation of DLPFC transcript expression

RNA-Seq was performed on blocks of DLPFC from a subset of ROS and MAP participants as previously described [8]. RNA was extracted from DLPFC blocks and quantified by Nanodrop (Thermo Fisher Scientific, Waltham, MA, US). An Agilent Bioanalyzer was used to assess quality. Samples from which less than 5-μg of RNA were obtained and samples with an RNA integrity (RIN) score of 5 or less were excluded from further analysis. The strand-specific dUTP method [34] with poly-A selection [35] was used by the Broad Institute Genomics Platform to prepare the RNA-Seq library. Poly-A selection was followed by first-strand-specific cDNA synthesis, with dUTP used for second-strand-specific cDNA synthesis, followed by fragmentation and Illumina adapter ligation for library construction. An Illumina HiSeq machine was used to perform sequencing with 101-bp paired-end reads, achieving a coverage of 150M reads for the first 12 samples, which served as a deep coverage reference. The remaining samples were sequenced with coverage of 50M reads. Next, beginning and ending low-quality bases and adapter sequences were trimmed from the reads, and ribosomal RNA reads were removed. The Bowtie 1 software package [36] was used to align the trimmed reads to the reference genome. Finally, the RSEM software package was used to estimate, in units of fragments per kilobase per million mapped fragments (FPKM), expression levels for 55,889 individual GENCODE v14 genes, which were then quantile-normalized, correcting for batch effect with Combat [37]. These data are available through the Synapse AMP-AD Knowledge Portal (Synapse ID syn3388564; 10.7303/syn3388564).

We used a consensus clustering method called SpeakEasy [38,39] to robustly define co-expressed molecular systems based on the gene–gene correlation matrix. Briefly, this method clusters the data hundreds of times under slightly different conditions, and selects the most representative clustering. SpeakEasy is relatively insensitive to parameter selection, is able to utilize negative correlations in the clustering process, minimizes unclassified “gray module” nodes, and shows good performance on synthetic clustering benchmarks including the Lancichinetti-Fortunato-Radicchi benchmarks [38]. Applying this approach to our RNA-Seq data generated 257 modules, of which 47 ranged in size from 20 to 556 genes. In all, 98% of all genes were assigned to one of these 47 modules. This approach yielded very similar results to standard WGCNA algorithms [40] when applied to our data [39].

For each gene in each module, we computed the normalized expression level by subtracting the mean expression level for that gene across all individuals, and dividing by the standard deviation. Then, we summarized the expression level of each module in each individual by computing the mean of the normalized expression levels of all genes in that module.

Data from 507 samples meeting quality control criteria as above, and with full clinical data, were included in this analysis (Table 1).

### Statistical analysis

A summary of the statistical analyses is provided here. For a full description see S1 Methods.

For all analyses, only observations with complete data for each analysis were used.

The main outcomes of interest were cognitive performance considered as a continuous variable, combined clinical diagnosis of MCI or dementia considered as a categorical variable, levels of CSF AD biomarkers (including Aβ 42, Aβ 40, tau, and phospho-tau) considered as continuous variables, and DLPFC expression of GENCODE v14 genes, considered as continuous variables. The main predictor of interest was date relative to the calendar year (of cognitive assessment for the cognitive outcomes, of lumbar puncture for CSF markers, and of death for postmortem RNA-Seq) considered as an angular continuous variable. Potential confounders included age (at time of cognitive evaluation, lumbar puncture, or death) considered as a continuous variable, sex considered as a dichotomous variable, years of education considered as a continuous variable, number of depressive symptoms considered as a continuous variable, hours of sleep considered as a continuous variable, hours of physical activity considered as a continuous variable, serum TSH level as a continuous variable, and clock time of assessment or death considered as a categorical variable in 1-hour bins. Potential effect modifiers included age (continuous), sex (dichotomous), and race (categorical).

In the ROS, MAP, and MARS cohorts, we characterized seasonal patterns in cognition by considering cognition as a linear function of the cosine of the date of evaluation, adjusted for age at evaluation, sex, and level of education (S1 Fig). We determined the significance of rhythmicity by comparing this model fit to one without the cosine term using an F-test. We extracted the amplitude, and the timing of the acrophase (peak) and nadir (trough) of the cosine curve (S1 Fig). We calculated standardized amplitudes by dividing the amplitude by the standard deviation of cognitive function across the cohort at baseline. Moreover, we contextualized the magnitude of this amplitude by comparing it to the magnitude of the effect estimate for age from Equation 1 in S1 Methods.

Using this approach, in our primary analyses, we assessed seasonal rhythmicity in composite global cognitive function at the baseline evaluation in the ROS and MAP cohorts, controlling for age, sex, and level of education, and including only those participants without MCI or dementia. We then did the same for the MARS cohort. Then, we analyzed the ROS, MAP, and MARS cohorts together, controlling for the effect of study cohort as a fixed effect. Although multiple annual cycles of cognitive data are available in some ROS, MAP, and MARS participants, the design of these studies is such that participants are assessed at roughly the same date across cycles (interquartile range −13 to +11 days). Because of the high intra-participant correlation of assessment dates, adding data from multiple cycles per participant would not substantially improve our estimates of seasonal effects nor would it allow for effective estimation of intra-participant seasonal effects. Therefore, in the interests of analytical parsimony, we did not include multiple cycles of data in our primary analyses.

Cognition may be affected by a number of potential confounders that may also show seasonal variation, including time of cognitive testing, depression, hours of sleep, and physical activity. To account for these, we considered models adjusted for time of cognitive testing (considered as a categorical variable by hour and available in the ROS, MAP, and MARS cohorts), number of depressive symptoms (considered as a continuous variable and available in the ROS, MAP, and MARS cohorts), hours of sleep (considered as a continuous variable and available in the ROS and MAP cohorts only), and hours spent engaged in physical activities (considered as a continuous variable and available in the ROS and MAP cohorts only). Moreover, in June 2018, we considered an additional model adjusted for serum level of TSH. Assessment of TSH was added to the ROS and MAP assessment protocols only after 2002, and so data on serum TSH levels were not available for nearly 50% of ROS and MAP participants at their baseline assessment. In contrast, data on serum TSH levels were available for 97% of MARS participants at their baseline assessment. Therefore, the analysis of TSH was restricted to the MARS cohort.

Next we examined whether seasonal rhythms in cognition may vary by specific cognitive domain. To do so, in the combined ROS, MAP, and MARS cohorts, we repeated our primary analyses, considering separately summary scores in the specific domains of working memory, perceptual speed, visuospatial ability, semantic memory, and episodic memory.

Seasonal variation in cognition may plausibly lead to seasonal variation in the diagnosis of MCI or dementia. To assess this, we followed the ROS, MAP, and MARS participants from our primary analyses above, and considered their clinical diagnosis at the last available cognitive evaluation. In June of 2018, we repeated our primary analysis, considering the last available in lieu of the baseline cognitive evaluation. Then, we considered the odds of being classified as having MCI or dementia as a function of the cosine of the date of assessment, adjusted for age, sex, education, and source cohort. To quantify the contribution of seasonal rhythmicity to the odds of being classified as having MCI or dementia, we compared this model to a reduced equation without the cosine term using a likelihood ratio test.

We also considered season as a categorical variable, defining winter/spring as January–June and summer/fall as July–December, and considered the effect of categorical season on the odds of being diagnosed with MCI/dementia, comparing this model to a reduced equation without the seasonal term with a likelihood ratio test. In June of 2018, we also considered models adjusted for time of cognitive testing, number of depressive symptoms, hours of sleep, hours of physical activity, and serum level of TSH.

Next, we examined whether AD pathology is associated with differential seasonal rhythmicity of cognition. To do so, we repeated our primary analyses above using the last known cognitive assessments from deceased ROS, MAP, and MARS participants with and without AD pathology, defined as an NIA-Reagan classification of intermediate or high, at death. We considered an augmented model, allowing for independent effects of AD pathology on the level and rhythmicity of cognition.

Next, we repeated our primary analyses in the SDS cohort with the total DRS score as the primary outcome, and then with the DRS subscores, as well as scores on the digit span test, Digit Symbol Substitution Test, California Verbal Learning Test, semantic fluency (animal naming) test, Wisconsin Card Sorting Test, and Benton Line Orientation Test as secondary outcomes, all considered as continuous variables.

Pathophysiological processes underlying AD, including amyloid and tau metabolism, are important contributors to impaired cognition in older adults. To examine for evidence of seasonal rhythmicity in amyloid and tau biology, we examined CSF Aβ 40, Aβ 42, tau, and phospho-tau in the CNC cohort as a function of date of lumbar puncture, adjusted for age and sex, using an identical approach as used for cognition above, considering first all participants together, adjusted for diagnosis (AD versus non-AD), and then allowing for differences in level and rhythmicity between participants with and without AD.

Next, we set out to identify co-expressed molecular systems that may be related to seasonal rhythms in cognition. We considered a set of co-expressed genes (module) to be potentially related to seasonal rhythms in cognition if it fulfilled all of the following: (1) expression was seasonally rhythmic, (2) expression was either in phase or antiphase to the rhythm of cognition, and (3) expression was associated with cognitive performance proximate to death. To assess seasonal rhythmicity, we considered the mean expression level of each gene module as a function of date and time of death, adjusted for age at death, sex, level of education, and methodological covariates such as batch, postmortem interval, and RNA quality (RIN score), and accounting for multiple comparisons by permutation, as described previously [8]. A module was considered seasonally rhythmic if its *p*-value, adjusted for multiple comparisons, was <0.05. To assess association with cognition proximate to death, we considered the composite global cognition proximate to death as a linear function of mean module expression, adjusted for age, sex, education, and methodological covariates including postmortem interval, batch, and RNA quality (RIN score), while accounting for multiple comparisons by generation of 10,000 null datasets created by permuting module labels. A module was considered associated with cognition if its *p*-value, adjusted for multiple comparisons, was <0.05. In June of 2018, we utilized the same approach to relate module expression to burden of amyloid pathology in autopsy. In June of 2018, we also characterized cognitive rhythms proximate to death by repeating our primary analyses on the subset of ROS and MAP participants with RNA-Seq data available, using the last available cognitive assessment proximate to death, and considering composite global cognitive function, as well as the 5 cognitive domains.

For co-expression modules meeting the above criteria, we controlled for potential confounders including depression and neuropathologies potentially affecting cognition—including the burden of AD pathology considered as a continuous measure, the presence or absence of cortical Lewy bodies considered as a dichotomous variable, and the number of macroscopic and microscopic cortical infarcts considered as continuous measures—by repeating the above analysis in models adjusted for these covariates. We also examined the association of AD pathology with the level and rhythmicity of module expression by considering augmented models similar to the analysis we performed with cognition in Equations 10, 11, and 12 in S1 Methods.

We next examined associations between module expression and other cognition-related phenotypes including the individual cognitive domains, Mini-Mental State Examination scores, and measures of AD pathology by calculating Spearman correlations between mean module expression at death and either cognitive phenotypes at the last measurement proximate to death or neuropathological phenotypes at death, adjusting for multiple comparisons by Bonferroni correction.

Finally, we examined transcription factor binding sites associated with the 4 co-expression modules putatively associated with seasonal rhythms of cognition. To do so, we used genome-wide annotated binding sites for 161 transcription factors from the ENCODE project [41–43]. We considered a transcript to be locally associated with a transcription factor if its transcription start site overlapped with one of the ENCODE-annotated transcription factor binding sites or was within 2,000 bp of it. For each of the 4 co-expression modules, we then used logistic regression models to examine the independent association of the local presence of binding sites for each of the 161 ENCODE transcription factors with the odds of a transcript being a member of a given co-expression module. We corrected for multiple comparisons using Bonferroni correction for 161 transcription factors (α = 0.05/161 = 0.0003).

## Results

### Study participants

Characteristics of the study participants are given in Table 1.

### Association between season and cognition in older adults without cognitive impairment

**Fig 1:**
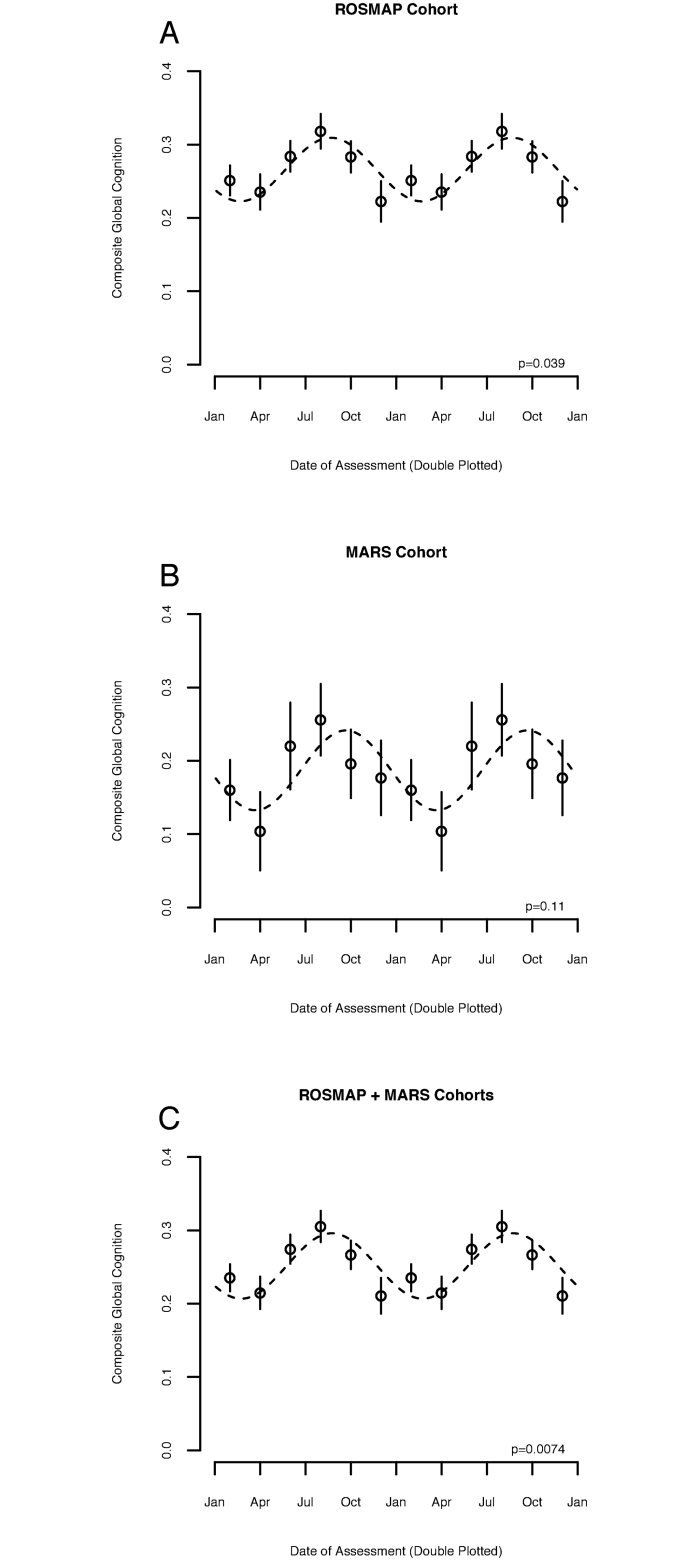
Association between season and cognitive function in adults without cognitive impairment. Composite global cognitive performance as a function of date of assessment, double plotted. Circles indicate means of 2-month bins. Bars indicate standard errors of the mean. Dotted lines indicate fit cosine curve. *p*-Value indicates significance of contribution of seasonal rhythmicity to overall variance, calculated as described in the text. (A) Baseline measurements for the Religious Orders Study (ROS) and Rush Memory and Aging Project (MAP) cohorts combined (*n =* 2,234). (B) Baseline measurements for the Minority Aging Research Study (MARS) cohort (*n =* 527). (C) Baseline measurements for the combined ROS, MAP, and MARS cohorts considered together (*n =* 2,761).

We first examined the association between season and baseline cognition in participants in the ROS and MAP without cognitive impairment (Table 1; *n =* 2,234). The ROS and MAP are longitudinal cohort studies of aging in which participants are free of dementia at study enrollment and agree to brain donation upon death. The mean (SD) baseline composite global cognitive score among participants without cognitive impairment in the combined ROS and MAP cohort was 0.26 (0.44) units. In an unadjusted model, season had a significant association with composite global cognitive function, with the highest average composite global cognition seen just before the fall equinox (*p =* 0.004). Compared to participants assessed at the winter/spring trough, the composite global cognitive score of participants assessed at the summer/fall peak was 0.20 standard deviations (a measure of the magnitude of seasonal variation relative to overall variability) higher (95% CI 0.09 to 0.32, *p =* 0.004). In a model adjusted for age, sex, and education, the effect of season on composite global cognition was similar, with a peak before the fall equinox (Fig 1A). Compared to participants assessed in the winter and spring, the composite global cognitive score of participants assessed in the summer and fall was 0.13 SD higher (95% CI 0.04 to 0.24, *p =* 0.039)—equivalent to that of participants 4.4 years younger (95% CI 1.8 to 8.6, *p =* 0.018). In the African-American MARS cohort (Table 1; *n =* 527), the mean (standard deviation) composite global cognitive score was 0.19 (0.47). In the MARS cohort, in a model adjusted for age, sex, and education, the magnitude of the seasonal effect was similar to that in the ROS and MAP cohorts, with 0.21 SD of composite global cognition (95% CI 0.06 to 0.47, *p =* 0.109) separating participants evaluated at the winter/spring nadir and summer/fall acrophase, equivalent to an age difference of 4.3 years (95% CI 1.29 to 9.26, *p =* 0.064). The *p*-value in the MARS cohort was somewhat attenuated, although this may reflect the smaller number of MARS participants (*n =* 527) compared to ROS/MAP participants (*n =* 2,234) in these analyses. Pooling data across all 3 cohorts (Table 1) enhanced the effect (Fig 1C; amplitude = 0.14 SD [95% CI 0.07 to 0.23], *p =* 0.007, with the peak to trough difference equivalent in effect to a 4.8-year difference in age [95% CI 2.1 to 8.5], *p =* 0.009). The association between season and cognition did not vary by age, sex, race, or source cohort (*p >* 0.05 for interaction terms). Moreover, inclusion of terms for time of cognitive test, depression, sleep, physical activity, or serum level of TSH did not substantially change estimates of these seasonal effects compared to models without these terms (S1 Table). The timing of rhythms of cognitive performance was similar across cognitive subdomains (S2 Fig), with peak performance near the fall equinox. However, the seasonal effect was strongest for working memory and perceptual speed and weaker for the other domains.

### Association between season and clinical diagnosis

**Fig 2:**
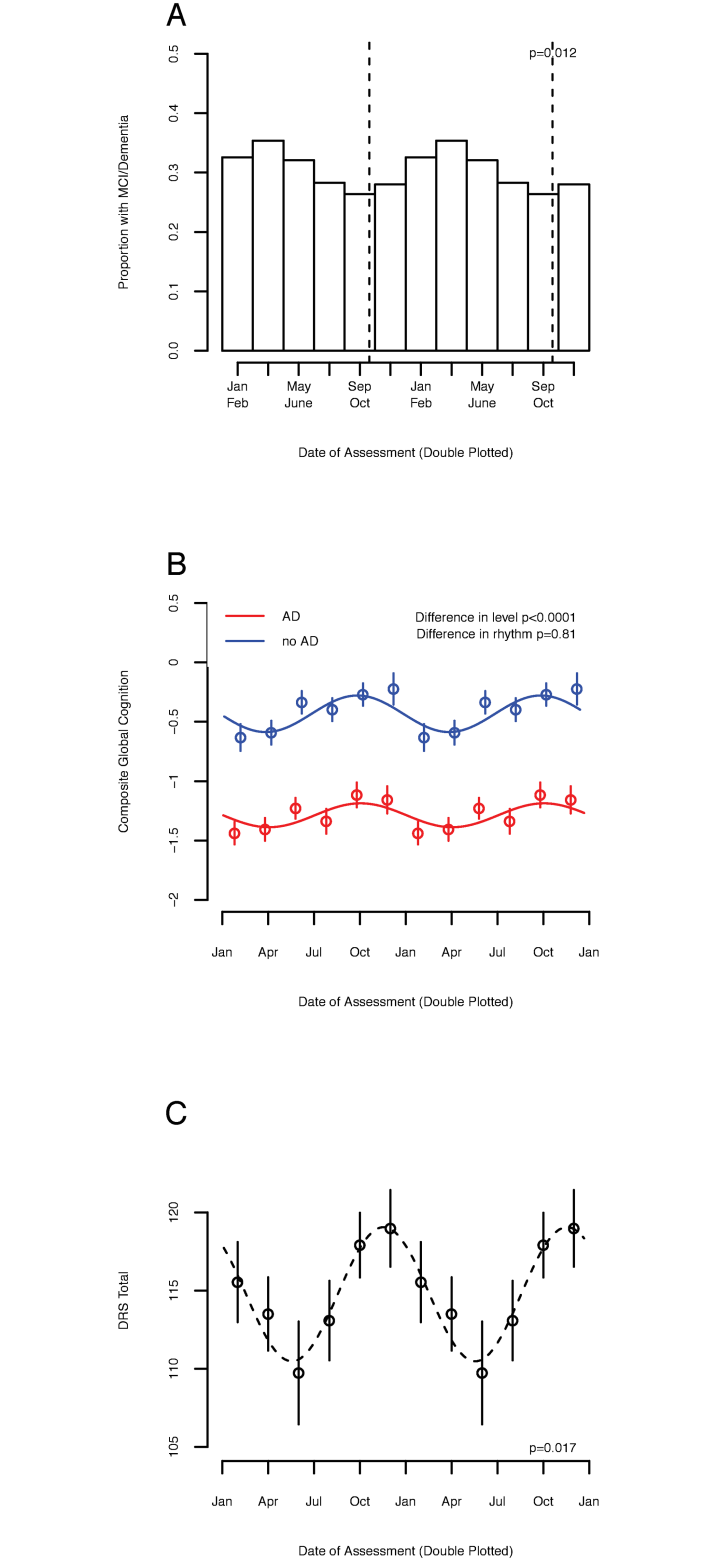
Association between season and clinical diagnosis and cognitive function in adults with Alzheimer disease (AD). (A) Proportion of participants classified as having mild cognitive impairment (MCI) or dementia, in 2-month bins double plotted, considering the last available assessment in the Religious Orders Study (ROS), Rush Memory and Aging Project (MAP), and Minority Aging Research Study (MARS) cohorts together (*n =* 2,761). Dotted line indicates the timing of the acrophase of composite global cognition. *p*-Value indicates significance of contribution of seasonal rhythmicity to the log-odds of being classified as having MCI or dementia. (B) Composite global cognitive performance as a function of date of assessment, double plotted, stratified by presence/absence of a pathological diagnosis of AD, in decedents from the ROS, MAP, and MARS cohorts (*n =* 1,410). Circles indicate means of 2-month bins. Bars indicate standard errors of the mean. Lines indicate fit cosine curves. Red = without a pathological diagnosis of AD. Blue = with a pathological diagnosis of AD. *p*-Values indicate significance of difference in either level or rhythmicity between participants with and without a pathological diagnosis of AD, as calculated in the text. (C) Total Dementia Rating Scale (DRS) score as a function of date of assessment, double plotted, for the baseline assessment of participants in the SDS cohort (*n =* 271). Circles indicate means of 2-month bins. Bars indicate standard errors of the mean. Dotted line indicates fit cosine curve. *p*-Value indicates significance of contribution of seasonal rhythmicity to overall variance, calculated as described in the text.

We hypothesized that the association of season with cognition would be reflected in rates of cognitive diagnoses. To test this, we analyzed the last available assessment for ROS, MAP, and MARS participants (Table 1; a median [IQR] of 6 [3 to 11] years since study enrollment). In a model adjusted for age, sex, and education, there persisted significant seasonal rhythmicity of composite global cognitive performance at the last available wave of data (amplitude = 0.13 SD [95% CI 0.05 to 0.24], *p =* 0.022). Results were similar when we restricted these analyses to deceased participants in whom RNA-Seq data were available (S3 Fig). We then used logistic regression models adjusted for age, sex, education, and source cohort to model the association between assessment date and the odds of meeting criteria for MCI or dementia. Of the 2,761 participants, 813 (29.5%) met diagnostic criteria for MCI or AD. In unadjusted models, the effect of season on diagnosis was significant (deviance = −10.3, *p =* 0.006). A participant evaluated in the winter or spring (January–June) had a roughly 24% higher odds of meeting criteria for MCI or dementia than one evaluated in the summer or fall (July–December; odds ratio 1.24 [95% CI 1.05–1.47], *p =* 0.008). In models adjusted for age, sex, and education, the effects were similar (Fig 2A; deviance = −10.2, *p =* 0.006). In these adjusted models, a participant evaluated in the winter or spring (January–June) had a higher odds of meeting criteria for MCI or dementia than one evaluated in the summer or fall (July–December; odds ratio 1.31 [95% CI 1.10–1.57], *p =* 0.003). This effect remained similarly significant in models adjusted for time of test, number of depressive symptoms, self-reported hours of sleep, self-reported hours of physical activity, or serum level of TSH (S2 Table).

### Association between season and cognition in adults with AD

AD affects circadian rhythmicity and may plausibly affect seasonal rhythmicity. To examine the association between pathologically confirmed AD and seasonal cognitive rhythms, we analyzed the last available assessment in ROS, MAP, and MARS decedents (Table 1; *n =* 1,410). The median (IQR) time between last cognitive assessment and death was 282 days (152–434). Considering all participants, there remained a significant association between season and composite global cognition (Fig 2B; amplitude = 0.23 SD [95% CI 0.11 to 0.38], *p =* 0.026). Participants with pathological AD had lower mean composite global cognition than those without (estimate = −0.65 SD, SE = 0.05, *p* < 0.0001); however, the seasonal rhythmicity of cognition did not differ significantly between these groups (*p =* 0.81), suggesting preserved seasonality of cognition in pathological AD.

To assess generalizability, we replicated our analyses in baseline data from the Canadian SDS cohort, an observational study of cases from a tertiary care memory clinic in Toronto, Canada. The mean (SD) Mattis DRS score of participants with AD was 115 (17). In a model adjusted for age, sex, and years of education, the association between season and the total DRS score was significant (amplitude 0.50 SD [95% CI 0.07 to 0.66], *p =* 0.017; Fig 2C). The highest model-predicted DRS scores were seen in the fall (November 16), with an average difference of 8 points between participants evaluated at the spring nadir (May 18) and fall peak. There were strong effects on the DRS subdomains of attention, speed, and initiation, and weaker effects on other domains (S4 and S5 Figs).

### Seasonal rhythms of CSF AD biomarkers

**Fig 3:**
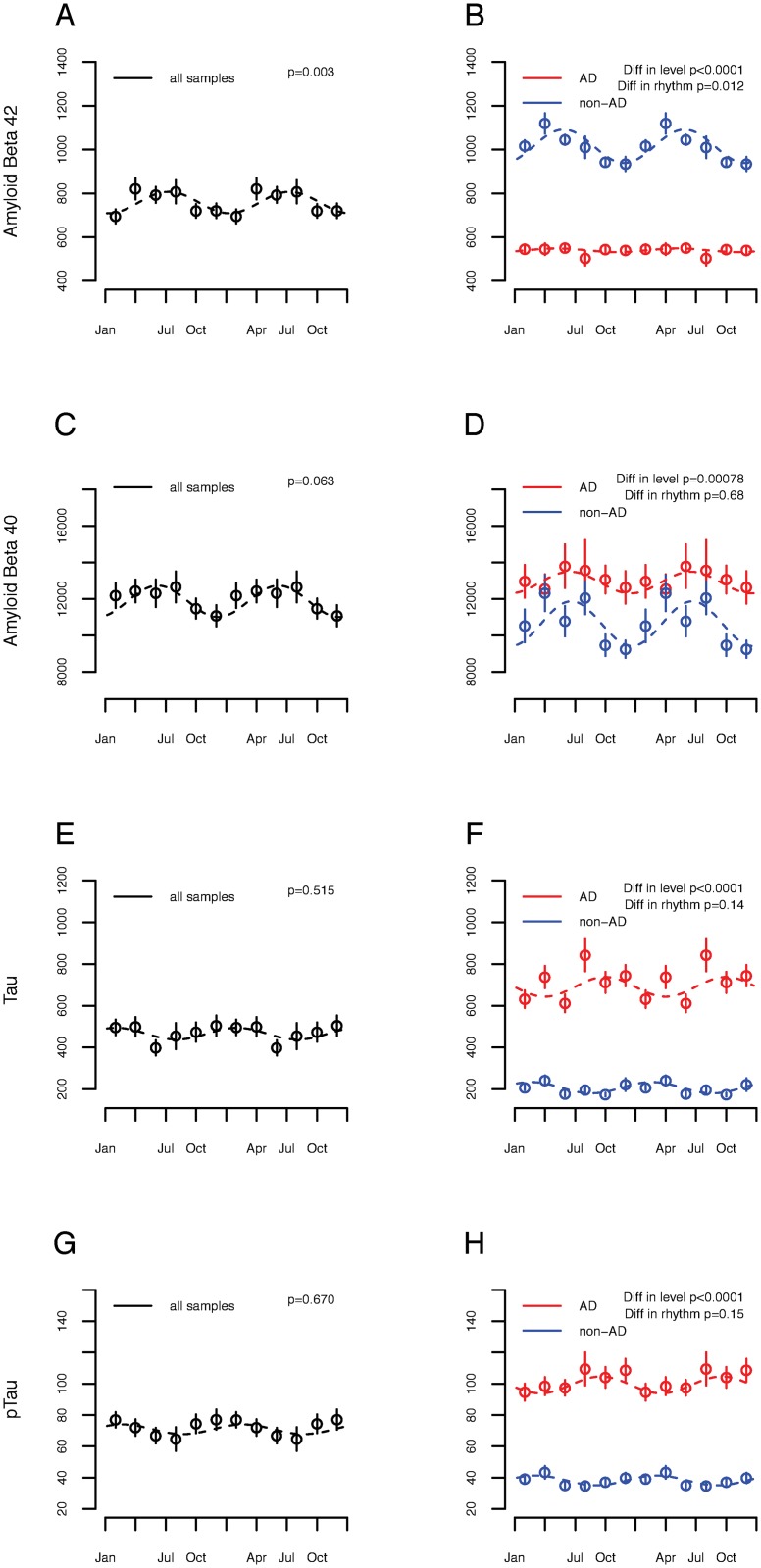
Association between season and Alzheimer disease (AD) cerebrospinal fluid biomarkers. Concentrations of AD biomarkers in the cerebrospinal fluid of Centre de Neurologie Cognitive (CNC) participants as a function of date of lumbar puncture (*n =* 321). Circles indicate means of 2-month bins. Bars indicate standard errors of the mean. Dotted lines indicate fit cosine curves. (A, C, E, and G) All CNC participants considered together. (B, D, F, and H) Stratified by clinical diagnosis of AD (red) versus non-AD (blue) cognitive impairment. *p*-Values indicate significance of difference in either level or rhythmicity between participants with and without a clinical diagnosis of AD, as calculated in the text. (A and B) Aβ 42. (C and D) Aβ 40. (E and F) Tau. (G and H) Phosphorylated tau.

Amyloid and tau pathology contribute to cognitive impairment in older adults [44], and CSF amyloid levels are diurnally rhythmic [45]. To test for an association between season and amyloid and tau biology, we analyzed CSF amyloid and tau levels in patients with (*n =* 176) and without (*n =* 145) clinical AD from the CNC cohort, an observational cohort study of patients from a tertiary care memory center in Paris, France (Table 1; *n =* 321). The mean (SD) CSF Aβ 42, Aβ 40, tau, and phospho-tau levels were 754 (280), 11,835 (4,856), 469 (318), and 71 (40) pg/ml, respectively. Considering all participants together, in a model adjusted for age and sex, CSF Aβ 42 level was strongly rhythmic, peaking in late June (amplitude 0.30 SD [95% CI 0.10 to 0.64], *p =* 0.003; Fig 3A). In the models adjusted for age and sex, season was not significantly associated with Aβ 40 (*p =* 0.06; Fig 3C). Tau and phospho-tau were not rhythmic (*p =* 0.515 for tau; *p =* 0.67 for phospho-tau; Fig 3E and 3G). Patients with AD had lower CSF Aβ 42 and higher Aβ 40, tau, and phospho-tau, as expected (Fig 3B, 3D, 3F and 3H). Moreover, the rhythmicity of Aβ 42 was attenuated in participants with AD (Fig 3B).

### Association between season and neocortical gene expression

Seasonal rhythms of gene expression correlate with tissue function in other organ systems such as the immune system [46]. To identify genes linking season to cognition, we used RNA-Seq to quantify gene expression in postmortem DLPFC samples from 507 ROS and MAP participants, as previously reported [8]. The DLPFC is a key node for working memory, the cognitive domain most strongly affected by season in our data. We analyzed groups of co-expressed genes called “modules,” which are detected as clusters of correlated genes. These modules are driven by cellular or regulatory mechanisms controlling a wide range of functions [47] and are more robust markers of biological functions than individual genes. We considered a module as potentially linking season to cognition if its expression was (1) itself seasonally rhythmic, (2) in phase or antiphase with cognition, and (3) associated with cognition proximate to death. Of 47 modules examined, in models adjusted for age, sex, education, time of death, postmortem interval, and methodological variables such as batch, postmortem interval, and RNA quality (RIN score), 4 modules (labeled arbitrarily m6: *n =* 328 genes, amplitude = 0.44 SD [95% CI 0.21 to 0.65], *p =* 0.0021; m13: *n =* 353 genes, amplitude = 0.46 SD [95% CI 0.27 to 0.76], *p =* 0.0009; m109: *n =* 390 genes, amplitude = 0.43 SD [95% CI 0.24 to 0.67], *p =* 0.0021; and m122: *n =* 370 genes, amplitude 0.46 SD [95% CI 0.20 to 0.71], *p =* 0.0012) met all 3 criteria (S3 Table).

**Fig 4:**
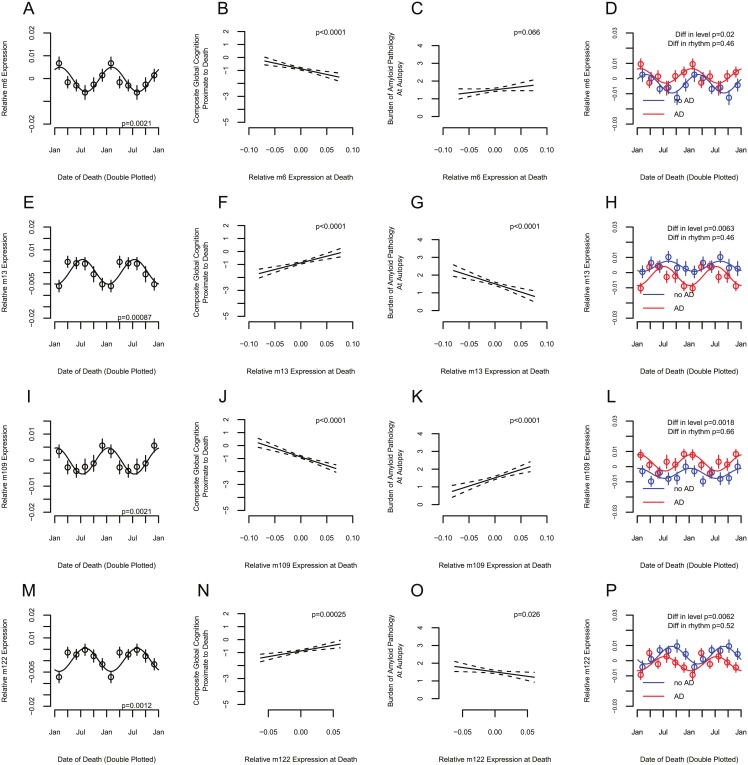
Association between season and the expression of cognition-associated co-expressed molecular systems. Co-expressed molecular systems (modules), comprised of gene sets putatively associated with seasonal rhythmicity of cognition, as operationally defined in the text (*n =* 507). (A, D, E, H, I, L, M, and P) Mean module expression as a function of date of death, double plotted. Circles indicate means of 2-month bins. Bars indicate standard errors of the mean. Dotted lines indicate fit cosine curves. (A, E, I, and M) All participants considered together; *p*-Value indicates significance of contribution of seasonal rhythmicity to overall variance, calculated as described in the text. (D, H, L, and P) Stratified by presence (red) or absence (blue) of pathological diagnosis of Alzheimer disease (AD). *p*-Values indicate significance of difference in either level or rhythmicity between participants with and without a pathological diagnosis of AD, as calculated in the text. (B, C, F, G, J, K, N, and O) Association between composite global cognition at last measurement before death (B, F, J, and N) or burden of amyloid pathology at death (C, G, K, and O) and module expression at death. Solid line indicates model-predicted response for unadjusted model. Dotted lines indicate 95% confidence intervals on the prediction. *p*-Value is adjusted for covariates as indicated in the text.

**Fig 5:**
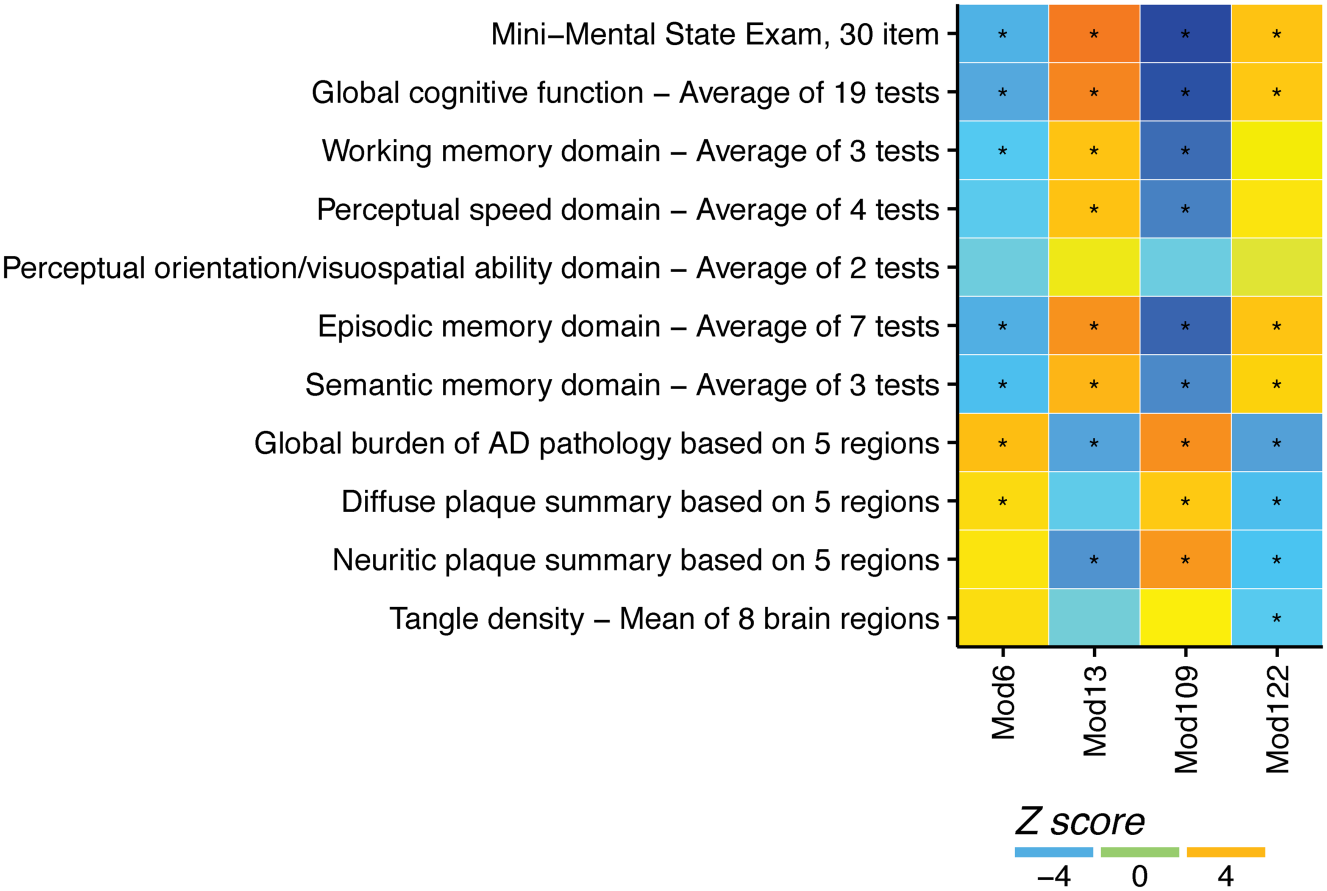
Association of gene modules with other cognitive and neuropathological phenotypes. Colors indicate *z*-scores; asterisks indicate significance after Bonferroni correction for multiple comparisons (*n =* 507). AD, Alzheimer disease; Mod, module.

The genes in these modules are in S4 Table. Two modules, m13 and m122, showed peak expression at the time of peak cognition (Fig 4A–4D and 4I–4L). Higher expression of these modules was associated with better cognition proximate to death, and with a lower burden of amyloid pathology at autopsy. The other 2 modules, m6 and m109, showed peak expression at the time of poorest cognition (Fig 4E–4H and 4M–4P). Higher expression of these modules was associated with worse cognition proximate to death, and with a higher burden of amyloid pathology at autopsy. These modules remained associated with season and cognition in models adjusted for depression and other neuropathologies (S5 Table). They were also associated with other cognitive and neuropathological phenotypes (Fig 5). Module rhythmicity was similar in those with and without AD pathology (*p >* 0.05 for all 4 modules).

Finally, we used genome-wide annotated binding sites for 161 transcription factors from the ENCODE project [41] to identify sites associated with each module. After correction for multiple comparisons by permutation, 15, 13, 6, and 9 transcription factor binding sites were associated with membership in modules m6, m13, m109, and m122, respectively (S6 Table). Of these, 5 (BCL11A, EGR1, MEF2C, and THAP1 associated with m109, and CTCF associated with m13) were themselves previously identified as seasonally rhythmic [8].

## Discussion

In this study of 3,353 older adults across multiple countries, cohorts, and races, there was a significant and reproducible association between season and cognition, with peak cognition near the fall equinox and a seasonal effect equivalent to a roughly 4-year difference in age. This association was independent of mood, sleep, physical activity, and thyroid status; it was clinically significant, as reflected in a nearly 30% higher odds of meeting criteria for MCI or dementia in winter and spring compared to summer and fall; and it persisted in cases with pathologically confirmed AD. Moreover, season was also associated with CSF Aβ levels and the brain expression of cognition-associated gene modules that were associated with identifiable transcription factor binding sites. Additional dementia care resources may be needed in the winter and spring, when cognition is likely to be worse. Moreover, these findings suggest substantial seasonal plasticity of cognition, amyloid biology, and cognition-related transcriptional programs, even in the presence of pathological AD. Seasonal factors may thus be important modifiers of the association of cognition with AD pathology, an effect potentially mediated by BCL11A, EGR1, THAP1, CTCF, MEF2C, and other key transcription factors, some of which are themselves seasonally rhythmic. Further work to understand the mechanisms underlying this plasticity may lead to novel environmental, behavioral, or pharmacological interventions to improve cognition in AD by enhancing the normal summer and fall peak in cognition, and/or extending it into other seasons.

Several previous studies found no association of season with cognition [3,6,7], while others showed association between season and some domains but not others [4,48], or only in individuals with psychopathology [5]. In contrast, we demonstrated a robust association of season with cognition in older adults with and without AD in multiple cohorts. Several factors may account for this divergence from previous studies. First, several previous studies examined cognition only near the solstices [4,6] or correlated cognition to day length [7]. If, as our data suggest, cognition peaks near the fall equinox, these approaches would fail to uncover significant seasonality. Second, several studies did not examine working memory [6,7], which in our data was the cognitive domain most affected by season. Third, it is possible that in younger adults, who were the subject of most previous studies, an abundance of cognitive reserve minimized the association between season and cognition, whereas this phenomenon becomes more important as cognitive reserve diminishes with cognitive aging. One study that found no association between season and cognition in young adults nevertheless found an association between season and cortical fMRI responses during a working memory task [3], with peak responses occurring near the fall equinox, roughly coincident with the peak in working memory observed in our study. This supports the hypothesis of a subclinical association between season and cognition in younger adults, seen on fMRI but not cognitive testing.

The persistence of a robust summer/fall peak in cognition suggests that even in pathologically confirmed AD, there remains substantial cognitive plasticity. Identifying drivers or mediators of this effect may enable leveraging this plasticity to improve cognition year-round. There are several hypotheses regarding potential mediators and drivers. First, the summer and fall peak in cognition may be driven by environmental factors such as light and temperature. If true, then interventions such as phototherapy or temperature modification may be effective in sustaining this peak year-round. Second, behavioral factors such as activity [49], sleep [50], and diet [51] show seasonality and may drive the summer and fall peak in cognition. In this study, the association between season and cognition was independent of self-reported sleep and physical activity, although studies incorporating objective markers of these and other behaviors may reveal a more important role for behavioral factors. Third, seasonal rhythms in psychological state (e.g., depression) may drive the association between season and cognition. In this study, the seasonality of cognition was independent of depression; however, other psychological factors, such as negative affect, which has been associated with MCI and dementia [52], may be important. While data on positive and negative affect were collected on some of our participants, they were available on too few participants to test their association with seasonal rhythms of cognition in this study. Fourth, seasonal rhythms in physiological state may potentially drive the association between season and cognition. In our study, adjusting for serum levels of TSH did not substantially attenuate estimates of the association between season and cognition. However, additional metabolic factors that may potentially link season to cognition are vitamin D [53], sex hormones like testosterone [54], and melatonin [55]. Unfortunately, data on serum levels of vitamin D, testosterone, and melatonin were not available in our study. Finally, an endogenous seasonal clock may drive rhythms in cognition. Supporting this, there is some evidence for such a clock in other species [56], and seasonal rhythms in fMRI responses to cognitive tasks persist even with control of behavioral and environmental factors [3]. Studies combining serial measurements of cognition, environment, behavior, and physiology in the same individuals at multiple time points across the year will help to distinguish these possibilities.

In the CNC cohort, CSF Aβ 42 level was highest in the summer, slightly preceding the time of peak cognition and lowest risk for MCI/dementia in the ROS, MAP, and MARS cohorts. This peak was unrelated to the clock time of lumbar puncture as all lumbar punctures were performed within a narrow 2- to 3-hour window each day. The phase difference between the CSF Aβ rhythms in the CNC cohort and the cognitive and molecular rhythms in the other cohorts may relate in part to differences in latitude (CNC 49° N versus south of 44° N for the other cohorts). The overlap between the timing of greatest CSF Aβ 42 levels and lowest odds of MCI/dementia is in keeping with existing literature showing that high CSF Aβ 42 levels are associated with better cognition even in older adults without a clinical diagnosis of AD [57]. Other studies have identified diurnal rhythms of CSF amyloid [45], but, to our knowledge, seasonal rhythms have not been described. These rhythms may reflect seasonal variation in Aβ 42 synthesis, or in transitions between compartments (parenchyma, interstitium, CSF, and systemic circulation) or forms (monomeric, oligomeric, and insoluble) of amyloid. CSF monomeric Aβ 42 concentrations are inversely related to brain interstitial oligomeric [58] and fibrillar [59] Aβ in some contexts. Therefore, low winter CSF monomeric Aβ 42 levels may reflect high levels of brain interstitial oligomeric Aβ species, which may have an adverse impact on cognition [60]. Studies examining the association between season and oligomeric Aβ species may shed light on this.

Irrespective of the ultimate drivers of the summer/fall peak in cognition, efforts to understand its neurobiological substrates may identify molecular pathways whose plasticity is preserved in pathological AD and which may therefore be promising targets to enhance cognitive function in AD. In this study, the summer/fall peak in cognition paralleled the seasonal nadirs of 2 prefrontal cortex co-expression gene modules (m6 and m109) negatively associated with cognition, and the seasonal acrophases of 2 gene modules (m13 and m122) positively associated with cognition. These phase relationships mean that the seasonal effects of these modules may be synergistic; peak cognition in the summer and fall may be driven by a synergy of higher expression of modules positively associated with cognition and negatively associated with Aβ (m13 and m122) and lower expression of modules negatively associated with cognition and positively associated with Aβ (m6 and m109), while in the winter and spring the opposite occurs. Gene ontology and cell type enrichment analyses for these modules have previously been published [39]. Although 3 of the 4 modules (m6, m13, and m122) are not strongly enriched for specific gene ontology pathways or cell types, m109 is relatively enriched for pathways involved in chromatin modification and cell cycle control, and is associated with cognitive decline and amyloid pathology [39]. In experiments in human cell lines, knock-down of selected m109 genes, including INPPL1 and PLXNB1, results in reduced extracellular amyloid levels, potentially linking seasonal rhythms of m109 to seasonal rhythms of brain extracellular amyloid [39]. Thus, one possible scenario is that in the summer and early fall, neocortical expression of m109 and associated genes such as INPPL1 and PLXNB1 is at its lowest, leading to relatively lower levels of brain interstitial oligomeric Aβ species (reflected by relatively high CSF monomeric Aβ levels), which leads to improved cognition, while in the winter and early spring the opposite is true (S6 Fig). Additional work is needed to examine the cognitive impact of increasing or decreasing expression of these modules in model systems, and to understand the mechanisms, such as the transcription factors identified in this study, that account for their rhythmicity. This may enable therapeutically increasing the expression of m13 and m122, or therapeutically decreasing the expression of m109 and m6 to enhance cognition year-round in individuals with AD.

Some limitations should be noted. First, each participant or sample contributed only 1 data point per annual cycle. It would be preferable to derive individual-level estimates of rhythmicity by repeated testing or sampling from the same individual throughout the year. However, to limit participant burden, we limited evaluations to once per year and CSF sampling to once per participant. Moreover, it would not be ethically permissible to repeatedly sample neocortical tissue from living participants. Second, we relied on self-report to measure environmental and behavioral factors leading up to each evaluation, and had limited quantitative data, making it difficult to distinguish the influence of these factors. Of particular note, as all of the study cohorts were based in relatively northern latitudes (north of 41° N), it is possible that extremes of weather, particularly cold, may have resulted in participants with greater degrees of neurological disability (e.g., those with greater cognitive impairment) being less likely to be able to attend study visits in the winter. However, this would have biased the study toward finding a lower likelihood of meeting diagnostic criteria for MCI or dementia in the coldest months, which is the opposite of the effect that was seen. Third, we had limited information on cause of death in decedents, which may influence gene expression at time of death, and which may be seasonal. Fourth, although this was one of the largest studies to date to examine seasonal rhythms of human brain gene expression, it nevertheless lacked statistical power to draw firm conclusions at the individual gene level, hence our analysis of gene sets defined by co-expression patterns. Fifth, this study consisted primarily of individuals of self-described European or African descent living in temperate regions of the northern hemisphere, limiting generalizability to other races or geographic locations. It would be of particular interest to study populations in temperate regions of the southern hemisphere, where one might expect seasonal effects to be in antiphase to those in the northern hemisphere, and to study populations in equatorial regions, where one might expect seasonal effects to be relatively attenuated compared to temperate regions. Finally, all cognitive and CSF assessments were performed during a relatively limited window within the 24-hour day—during daylight hours. While this limited the potential for confounding by time of assessment, it also limited the capacity to obtain robust estimates of circadian effects, which would require sampling around the 24-hour clock.

This study also had several strengths. Notwithstanding the population and geographic limitations noted above, we replicated our primary analyses in cohorts from 3 different countries, and 2 different racial groups, enhancing generalizability. Second, the temporal convergence of cognitive and molecular data enhanced biological plausibility and allowed us to draw links between molecular and behavioral rhythms. Third, cognitive assessments, lumbar punctures, and dates of death were spread throughout the year, rather than limited to discrete sampling times, maximizing the capacity to detect rhythmicity irrespective of phase. Fourth, we had histopathological confirmation in decedents, providing a high degree of diagnostic certainty.

Overall, we report robust estimates of an important source of variation in cognitive performance and its biological correlates among a diverse set of older individuals. Season should be considered as an important confounder when analyzing data from therapeutic trials and observational studies in AD. In particular, studies aiming to estimate rates of cognitive decline with repeated measurements of the same participant should either assess the same individual at the same date in different study cycles (as is done in the ROS, MAP, and MARS cohorts) or should consider season as a covariate. In clinical practice, seasonal effects may be an important source of diagnostic misclassification and may account for the observation that some individuals with MCI revert to normal cognition on subsequent testing. Our findings should also inform the design of clinical algorithms that leverage the winter/spring cognitive nadir to enhance sensitivity in identifying participants at the earliest stages of disease. Finally, the effect sizes seen in this study suggest that targeting the environmental or behavioral drivers of seasonal cognitive plasticity, or the key transcription factors and genes identified in this study as potentially mediating these effects, may allow us to substantially improve cognition in adults with and without AD pathology.

## Supporting information

S1 Fig Example of cosine curve fit to cognitive data. (EPS)

S2 Fig Association between season and cognitive function in adults without cognitive impairment—Individual cognitive domains. Cognitive performance (baseline measurements for ROS, MAP, and MARS cohorts considered together; *n =* 2,761) as a function of date of assessment, double plotted. Circles indicate means of 2-month bins. Bars indicate standard errors of the mean. Dotted lines indicate fit cosine curve. *p*-Value indicates significance of contribution of seasonal rhythmicity to overall variance, calculated as described in the text. (A) Working memory; (B) perceptual speed; (C) semantic memory; (D) episodic memory; (E) visuospatial ability.
(EPS)

S3 Fig Association between season and cognitive function in deceased ROS and MAP participants with available RNA-Seq data. Cognitive performance (deceased ROS and MAP participants with RNA-Seq data; *n =* 507) as a function of date of assessment, double plotted. Circles indicate means of 2-month bins. Bars indicate standard errors of the mean. Dotted lines indicate fit cosine curve. *p*-Value indicates significance of contribution of seasonal rhythmicity to overall variance, calculated as described in the text. (A) Composite global cognitive function; (B) working memory; (C) perceptual speed; (D) semantic memory; (E) episodic memory; (F) visuospatial ability.
(EPS)

S4 Fig Association between season and DRS subdomains in the SDS cohort. Cognitive performance as a function of date of assessment, double plotted (*n =* 271). Circles indicate means of 2-month bins. Bars indicate standard errors of the mean. Dotted lines indicate fit cosine curve. *p*-Value indicates significance of contribution of seasonal rhythmicity to overall variance, calculated as described in the text. (A) Initiation; (B) attention; (C) memory; (D) conceptualization; (E) construction.
(EPS)

S5 Fig Association between season and additional cognitive tests in the SDS cohort. Cognitive performance as a function of date of assessment, double plotted (*n =* 271). Circles indicate means of 2-month bins. Bars indicate standard errors of the mean. Dotted lines indicate fit cosine curve. *p*-Value indicates significance of contribution of seasonal rhythmicity to overall variance, calculated as described in the text. (A) Sum of digit span forward and backward; (B) Digit Symbol Substitution Test (number correct); (C) semantic fluency animal naming (number correct); (D) California Verbal Learning Test long delay free recall (words correct); (E) Benton Line Orientation Test (number correct); (F) Wisconsin Card Sorting Test (number correct).
(EPS)

S6 Fig Hypothesized relationship between seasonal rhythms of gene expression, amyloid biology, and cognition. (EPS)

S1 Methods Full description of statistical methods. (DOCX)

S1 STROBE checklist STROBE checklist for observational studies. (DOCX)

S1 Table Association between season and composite global cognitive function in adults without AD—Consideration of potential confounders at baseline. (DOCX)

S2 Table Association between season (winter/spring versus summer/fall) and odds of meeting criteria for MCI or dementia—Consideration of potential confounders at time of last available cognitive assessment. (DOCX)

S3 Table Association between season and the expression of cognition-associated molecular systems. Based on ROS, MAP, and MARS participants with available DLPFC RNA-Seq data (*n =* 507). Acrophase in radians. **In acrophase column: within 2 months of acrophase or nadir of cognition. Amplitude in standard units of composite global cognition. *p*-Value for rhythmicity adjusted for multiple comparisons by permutation. **In *p*-value for rhythmicity (adjusted) column: adjusted *p*-value < 0.05. *p*-Value for association with cognition adjusted for multiple comparisons by permutation. **In *p*-value for association with cognition (adjusted) column: *p <* 0.05.
(DOCX)

S4 Table Genes contained in m6, m13, m109, and m122. (DOCX)

S5 Table Association between season and the expression of cognition-associated molecular systems—Consideration of potential confounders. (DOCX)

S6 Table Transcription factor binding sites linked to cognition-associated molecular modules. Columns indicate *p*-values for association with module membership for each set of transcription factor binding sites as described in the text. **Significant after correcting for multiple comparisons. Transcription factors labeled with “++” were themselves found to have seasonally rhythmic transcript expression in a recent study [8].
(DOCX)

## Abbreviations

AD: Alzheimer disease
CNC: Centre de Neurologie Cognitive
CSF: cerebrospinal fluid
DLPFC: dorsolateral prefrontal cortex
DRS: Dementia Rating Scale
fMRI: functional MRI
MAP: Rush Memory and Aging Project
MARS: Minority Aging Research Study
MCI: mild cognitive impairment
NIA: National Institute on Aging
RIN: RNA integrity
RNA-Seq: RNA sequencing
ROS: Religious Orders Study
SDS: Sunnybrook Dementia Study
TSH: thyroid stimulating hormone
