# Supplementary material for: Seasonal plasticity of cognition and related biological measures in adults with and without Alzheimer disease: Analysis of multiple cohorts
Source: PLoS Med. 2018 Sep 4;15(9):e1002647. doi: 10.1371/journal.pmed.1002647 (PMC6122787; doi:10.1371/journal.pmed.1002647)
Supplement: S1 Methods — (DOCX) [file pmed.1002647.s007.docx]

**Supplementary Methods**

*Statistical Analysis*

For all analyses, only observations with complete data for each analysis were used.

The main outcomes of interest were cognitive performance considered as a continuous variable, combined clinical diagnosis of MCI or dementia considered as a categorical variable, levels of CSF AD biomarkers (including Aβ42, Aβ40, tau, and phospho-tau) considered as continuous variables, and dorsolateral prefrontal cortex expression of GENCODE v14 genes, considered as continuous variables. The main predictor of interest was date relative to the calendar year (of cognitive assessment for the cognitive outcomes, of lumbar puncture for CSF markers, and of death for post-mortem RNA-sequencing) considered as an angular continuous variable. Potential confounders included age (at time of cognitive evaluation, lumbar puncture or death) considered as a continuous variable, sex considered as a dichotomous variable, years of education considered as a continuous variable, number of depressive symptoms considered as a continuous variable, hours of sleep considered as a continuous variable, hours of physical activity considered as a continuous variable, serum thyroid stimulating hormone levels as a continuous variable, and clock time of assessment or death considered as a categorical variable in 1-hour bins. Potential effect modifiers included age (continuous), sex (dichotomous), and race (categorical).

In the ROS, MAP, and MARS cohorts, we characterized seasonal patterns in cognition by considering cognition as a function of date of evaluation, adjusted for age at evaluation, sex, and level of education as follows:

 (1)

where E(C) is the expected composite global cognitive function, d is the date of assessment in radians, A_s_ is the amplitude [peak to trough difference] of seasonal rhythmicity, ϕ_s_ is the acrophase [date of peak cognition] of seasonal rhythmicity, and β_1_x_1_ … β_n_x_n_ are the covariates age, sex, and years of education. For these analyses, the seasonal period was fixed at 365 days. This is a limitation of any study design where each individual contributes only 1 data point to 365-day sampling period. All dates were converted to radians (2π radians = 365 days; 0 radians = January 1) for analysis and then converted back to calendar dates for the purposes of visual representation.

For computational efficiency, we fit equivalent linearized models of the form

 (2)

and A_s_, and ϕ_s_ from equation (1) were calculated using the formulae

 (3)

 (4)

We calculated standardized amplitudes by dividing the amplitude from equation (3) by the standard deviation of composite global cognitive function across the cohort at baseline. Moreover, we contextualized the magnitude of this amplitude by comparing it to the magnitude of the effect estimate for age from equation (1). We determined 95% confidence intervals on both the standardized amplitude and amplitude relative to age by bootstrapping over 1000 iterations. Amplitudes were considered negative if more than pi/2 radians out of phase from the un-resampled data. Moreover, to obtain empiric p-values for the estimates of seasonal amplitude relative to age effects, we generated 1000 permuted null datasets by shuffling dates of assessment, estimated the amplitude of seasonal rhythmicity relative to the effect of age for each null dataset, and then computed the proportion of these null datasets for which the ratio was greater than seen in our observed data. To quantify the contribution of seasonal rhythmicity to the model fit in equation (2), we compared the residual sum of squares for equation (2) to that of a reduced model without the terms for seasonal rhythmicity

 (5)

and determined the F-statistic

 (6)

and associated 2-sided p-value with α=0.05. The greater the contribution of seasonal rhythmicity to the overall model fit, the greater the value of F_s_. To examine for effect modification by age, we augmented equation (2) with multiplicative interaction terms between age and the cosine and sine terms from equation (2) and compared this augmented model with equation (2), generating an F-statistic and p-value. We then repeated this for sex, self-reported race, and source cohort.

Using this approach, in our primary analyses, we assessed seasonal rhythmicity in composite global cognitive function at the baseline evaluation in the ROS and MAP cohorts, controlling for age, sex, and level of education, and including only those participants without MCI or dementia. We then did the same for the MARS cohort. Then, we analyzed the ROS, MAP, and MARS cohorts together, controlling for the effect of study cohort as a fixed effect. Although multiple annual cycles of cognitive data are available in some ROS, MAP, and MARS participants, the design of these studies is such that participants are assessed at roughly the same date across cycles (interquartile range -13 to +11 days). Because of the high intra-participant correlation of assessment dates, adding data from multiple cycles per participants would not substantially improve our estimates of seasonal effects nor would it allow for effective estimation of intra-participant seasonal effects. Therefore, in the interests of analytical parsimony, we did not include multiple cycles of data in our primary analyses.

Cognition may be affected by a number of potential confounders that may also show seasonal variation, including time of cognitive testing, depression, hours of sleep, and physical activity. To account for these, we considered models adjusted for time of cognitive testing (considered as a categorical variable by hour; available in the ROS, MAP, and MARS cohorts), number of depressive symptoms (considered as a continuous variable, available in the ROS, MAP, and MARS cohorts), hours of sleep (considered as a continuous variable, available in the ROS and MAP cohorts only), and hours spent engaged in physical activities (considered as a continuous variable; available in the ROS and MAP cohorts only). Moreover, in June 2018, we considered an additional model adjusted for serum levels of thyroid stimulating hormone (TSH). As assessment of TSH was added to the ROS and MAP assessment protocols only after 2002 and so data on serum TSH levels were not available on nearly 50% of ROS and MAP participants at their baseline assessment. In contrast, data on serum TSH levels were available on 97% of MARS participants at their baseline assessment. Therefore, the analysis of TSH was restricted to the MARS cohort.

Next we examined whether seasonal rhythms in cognition may vary by specific cognitive domain. To do so, in the combined ROS, MAP, and MARS cohorts, we repeated our primary analyses, considering separately summary scores in the specific domains of working memory, perceptual speed, visuospatial function, semantic memory, and episodic memory.

Seasonal variation in cognition may plausibly lead to seasonal variation in the diagnosis of mild cognitive impairment or dementia. To assess this, we followed the ROS, MAP and MARS participants from our primary analyses above, and considered their clinical diagnosis at the last available cognitive evaluation. In June of 2018, we repeated our primary analysis, considering the last available instead of the baseline cognitive evaluation. Then, we considered the odds of being classified as having MCI or dementia as a function of the date of assessment, adjusted for age, sex, education, and source cohort:

 (7)

where d is the date of last assessment in radians. To quantify the contribution of seasonal rhythmicity to the log odds of being classified as having MCI or dementia, we compared fit of equation (7) to a reduced equation without the seasonal terms using a likelihood ratio test, with 2-sided p-value α=0.05:

 (8)

We also considered season as a categorical variable, defining winter/spring as January-June and summer/fall as July-December, and considered the effect of categorical season on the log odds of being diagnosed with MCI/dementia, comparing to a reduced equation without the seasonal terms suing a likelihood ratio test, with 2-sided p-value α=0.05:

 (9)

In June of 2018, we also considered models adjusted for time of cognitive testing, number of depressive symptoms, hours of sleep, hours of physical activity, and serum levels of thyroid stimulating hormone.

Next, we examined whether Alzheimer’s disease pathology is associated with differential seasonal rhythmicity of cognition. To do so, we repeated our primary analyses above using the last known cognitive assessments from deceased ROS, MAP, and MARS participants with and without Alzheimer’s disease pathology, defined as an NIA-Reagan score of intermediate or higher, at death. To do so, we considered an augmented model, allowing for independent effects of AD pathology on the level and rhythmicity of cognition:

 (10)

To quantify the contribution of AD pathology to level of cognition, we used the F-test to compare equation (10) to a reduced model without the linear term for AD pathology, with a 2-sided p-value α=0.05:

 (11)

To quantify the contribution of AD pathology to the rhythmicity of cognition, we compared equation (10) to a reduced model without the terms for the AD effect on rhythmicity, with a 2-sided p-value α=0.05:

 (12)

Next, we repeated our primary analyses in the SDS cohort, with the total Dementia Rating Scale score as the primary outcome, and then with the Dementia Rating Scale subscores, as well as scores on the digit span test, Symbol Digit Substitution test, California Verbal Learning Test, Semantic Fluency (animal naming) test, Wisconsin Card Sort Test, and Benton Line Orientation Test as secondary outcomes, all considered as continuous variables.

Pathophysiological processes underlying Alzheimer’s disease, including amyloid and tau metabolism, are important contributors to impaired cognition in older adults. To examine for evidence of seasonal rhythmicity in amyloid and tau biology, we examined CSF Aβ40, Aβ 42 tau, and phospho-tau in the CNC cohort as a function of date of lumbar puncture, adjusted for age and sex, using an identical approach as used for cognition above, considering first all participants together, adjusted for diagnosis (AD vs. non-AD) similar to equations 2 and 4 above, and then allowing for differences in level and rhythmicity between participants with and without AD, similar to equations 10, 11, and 12 above but with CSF biomarker levels rather than cognition as the outcomes.

Next, we set out to identify co-expressed molecular systems that may be related to seasonal rhythms in cognition. We considered a set of co-expressed genes potentially related to seasonal rhythms in cognition if it fulfilled all the of the following: 1) it was seasonally rhythmic, 2) it was either in phase or antiphase to the rhythm of cognition and 3) it was associated with cognitive performance proximate to death. To assess seasonal rhythmicity, we considered the mean expression level of each gene module as a function of date and time of death, adjusted for age at death, sex, level of education, and methodological covariates such as batch, postmortem interval, and RNA quality (RIN score), and accounting for multiple comparisons by permutation, as described previously.[1] A module was considered seasonally rhythmic if its p-value, adjusted for multiple comparisons, was <0.05. To assess association with cognition proximate to death, we considered the composite global cognition proximate to death as a linear function of mean module expression, adjusted for age, sex, education, and methodological covariates including postmortem interval, batch, and RNA quality (RIN score), while accounting for multiple comparisons by generation of 10,000 null datasets created by permuting module labels. A module was considered associated with cognition if its p-value adjusted for multiple comparisons was <0.05. In June of 2018, we utilized the same approach to relate module expression to burden of amyloid pathology autopsy. In June of 2018, we utilized the same approach to relate module expression to burden of amyloid pathology autopsy. In June of 2018, we also characterized cognitive rhythms proximate to death by repeating our primary analyses on the subset of ROS and MAP participants with RNA-sequencing data available, using the last available cognitive assessment proximate to death, and considering both composite global cognitive function, as well as the 5 cognitive domains.

For co-expression modules meeting the above criteria, we controlled for potential confounders including depression and neuropathologies potentially affecting cognition including the burden of Alzheimer’s disease pathology considered as a continuous measure, the presence or absence of cortical Lewy bodies considered as a dichotomous variable, and the number of macroscopic and microscopic cortical infarcts considered as continuous measures by repeating the above analysis in models adjusted for these covariates. We also examined the impact of Alzheimer’s disease pathology on the level and rhythmicity of module expression by considering augmented models similar to the analysis we performed with cognition in equations 10, 11, and 12 above.

We next examined for associations between module expression and other cognition-related phenotypes including the individual cognitive domains, Mini-Mental state examination scores, and measures of AD pathology by calculating Spearman correlations between mean module expression at death, and either cognitive phenotypes at the last measurement proximate to death, or neuropathological phenotypes at death, adjusting for multiple comparisons by Bonferroni correction.

Finally, we examined for transcription factor binding sites associated with the 4 co-expression modules putatively associated with seasonal rhythms of cognition. To do so, we used genome-wide annotated binding sites for 161 transcription factors from the ENCODE project [2-4]. We considered a transcript to be locally associated with a transcription factor if its transcription start sites overlapped with one of the transcription factor ENCODE-annotated binding sites, or was within 2000bp of it. For each of the four co-expression modules, we then used logistic regression models of the form

(13)

to examine the independent association of the local presence of binding sites for each of the 161 ENCODE transcription factors with the odds of a transcript being a member of a given co-expression module. We corrected for multiple comparisons using Bonferroni correction for 161 transcription factors (α=0.05/161=0.0003).

**References for S1 Methods**

1. Lim AS, Klein HU, Yu L, Chibnik LB, Ali S, Xu J, et al. Diurnal and seasonal molecular rhythms in human neocortex and their relation to Alzheimer's disease. Nature communications. 2017;8:14931. doi: 10.1038/ncomms14931. PubMed PMID: 28368004; PubMed Central PMCID: PMCPMC5382268.

2. Wang J, Zhuang J, Iyer S, Lin XY, Greven MC, Kim BH, et al. Factorbook.org: a Wiki-based database for transcription factor-binding data generated by the ENCODE consortium. Nucleic Acids Res. 2013;41(Database issue):D171-6. doi: 10.1093/nar/gks1221. PubMed PMID: 23203885; PubMed Central PMCID: PMCPMC3531197.

3. Wang J, Zhuang J, Iyer S, Lin X, Whitfield TW, Greven MC, et al. Sequence features and chromatin structure around the genomic regions bound by 119 human transcription factors. Genome Res. 2012;22(9):1798-812. doi: 10.1101/gr.139105.112. PubMed PMID: 22955990; PubMed Central PMCID: PMCPMC3431495.

4. Gerstein MB, Kundaje A, Hariharan M, Landt SG, Yan KK, Cheng C, et al. Architecture of the human regulatory network derived from ENCODE data. Nature. 2012;489(7414):91-100. doi: 10.1038/nature11245. PubMed PMID: 22955619; PubMed Central PMCID: PMCPMC4154057.
