# Supplementary material for: Seasonal plasticity of cognition and related biological measures in adults with and without Alzheimer disease: Analysis of multiple cohorts
Source: PLoS Med. 2018 Sep 4;15(9):e1002647. doi: 10.1371/journal.pmed.1002647 (PMC6122787; doi:10.1371/journal.pmed.1002647)
Supplement: S1 STROBE checklist — (DOCX) [file pmed.1002647.s008.docx]

STROBE Statement—checklist of items that should be included in reports of observational studies

|  | Item No. | Recommendation | Section and Paragraph | Relevant text from manuscript |
| --- | --- | --- | --- | --- |
| **Title and abstract** | 1 | (*a*) Indicate the study’s design with a commonly used term in the title or the abstract | Title, Abstract | See below |
|  |  | **“Title:** Seasonal plasticity of cognition and related biological measures suggest new therapeutic targets for Alzheimer’s disease: analysis of multiple cohorts  **Abstract: Methods and Findings:** We analysed data from 3353 participants from three observational community-based cohort studies of older persons (the Rush Memory and Aging Project [MAP], the Religious Orders Study [ROS], and the Minority Aging Research Study [MARS]), and two observational memory-clinic based cohort studies (Centre de Neurologie Cognitive Hopital Lariboisiere [CNC] and the Sunnybrook Dementia Study [SDS]).” | | |
|  |  | (*b*) Provide in the abstract an informative and balanced summary of what was done and what was found | Abstract | See below |
|  |  | **Methods and Findings:** We analysed data from 3353 participants from three observational community-based cohort studies of older persons (the Rush Memory and Aging Project [MAP], the Religious Orders Study [ROS], and the Minority Aging Research Study [MARS]), and two observational memory-clinic based cohort studies (Centre de Neurologie Cognitive Hopital Lariboisiere [CNC] and the Sunnybrook Dementia Study [SDS]). We performed neuropsychological testing, and, in subsets of participants, evaluated for cerebrospinal fluid Alzheimer’s disease (AD) biomarkers, standardized structured autopsy measures, and/or prefrontal cortex gene expression by RNA-sequencing. We examined the impact of season on these variables using nested multiple linear and logistic regression models. Season had a robust impact on cognition that was replicated in multiple cohorts (amplitude=0.14SD [95% CI 0.07-0.23] p=0.0066 in the combined MAP, ROS, and MARS cohorts; amplitude=0.50SD [95% CI 0.07-0.66] p=0.017 in the SDS cohort). Average composite global cognitive function was higher in the summer and fall compared to winter and spring, equivalent in cognitive effect to 4.8 years’ difference in age [95%CI 2.4 to 8.0]. Further, the odds of meeting criteria for MCI or dementia were over 30% higher in the winter and spring (OR 1.31; 95% CI 1.10-1.57, p=0.003). These results were robust against multiple potential confounders including depression, and persisted in cases with AD pathology. Moreover, season had a marked effect on cerebrospinal fluid Aβ42 levels (amplitude 0.30SD [95% CI 0.10-0.64], p=0.003) which peaked in the summer, and on the brain expression of four cognition-associated modules of co-expressed genes (module 6: amplitude=0.44SD [95%CI 0.21-0.65], p=0.0021; module 13: amplitude=0.46SD [95% CI 0.27-0.76],p=0.00087; module 109: amplitude=0.43SD [95% CI 0.24-0.67], p=0.0021; and module 122: amplitude 0.46SD [95% CI 0.20-0.71], p=0.0012) which were in phase or anti-phase to the rhythms of cognition, and which were in turn associated with binding sites for several seasonally rhythmic transcription factors including BCL11A, CTCF, EGR1, MEF2C, and THAP1. Limitations include the evaluation of each participant or sample once per annual cycle, limited objective data onreliance on self-report for measurement of environmental and behavioral factors, and potentially limited generalizability to individuals in equatorial regions or in the southern hemisphere. | | |

| Introduction | | | |  |
| --- | --- | --- | --- | --- |
| Background/rationale | 2 | Explain the scientific background and rationale for the investigation being reported | Introduction paragraphs 1-2 | See below |
|  |  | Seasonal rhythms modulate several aspects of human behaviour and physiology including brain functions such as mood in seasonal affective disorder [1], symptom onset in schizophrenia [2], and fMRI brain responses to cognitive tasks [3]. Several studies suggest that season may modulate cognition in younger adults [4, 5], although this is not a universal finding [3, 6, 7], and data from older adults are lacking.  Demonstration of seasonal modulation of cognition and its neural substrates in older adults would have important clinical and translational implications. It would suggest that Alzheimer’s disease (AD) might be a seasonal illness, and that dementia care resources should be targeted to seasons of peak need, both to identify those at the earliest stages of disease, and to support patients when they are most vulnerable. It may account for the observation that some individuals with mild cognitive impairment (MCI) subsequently revert to normal cognition and may be an important source of diagnostic misclassification both in clinical trials and in clinical practice. Most importantly, it may shed light on mechanisms of cognitive plasticity that might be leveraged to improve cognition in patients with Alzheimer’s disease. | | |
| Objectives | 3 | State specific objectives, including any prespecified hypotheses | Introduction paragraph 3 | See below |
|  |  | In the present study of 3353 older adults with and without AD in the United States, France, and Canada, we tested the hypotheses that season has a significant impact on cognition, the odds of being diagnosed with MCI or dementia, cerebrospinal fluid (CSF) AD biomarkers, and the expression of cognition-associated modules of co-expressed genes in the human brain. | | |
| Methods | | | |  |
| Study design | 4 | Present key elements of study design early in the paper | Methods paragraph 1 | See below |
|  |  | We analyzed cross-sectional data at multiple time points from participants in three prospective observational community-based cohort studies of older persons (the Rush Memory and Aging Project [MAP cohort], Religious Orders Study [ROS cohort] and Minority Aging Research Study [MARS cohort]), and two prospective observational clinic-based case series (Centre de Neurologie Cognitive, Lariboisiere Hospital, University of Paris Diderot and Hopital Paris Assistance Publique [CNC cohort] and the Sunnybrook Dementia Study [SDS]). We related measures of cognition and clinical diagnoses of dementia to date of cognitive evaluation, cerebrospinal fluid biomarkers of Alzheimer’s disease to date of lumbar puncture, postmortem neocortical RNA-sequencing data to date of death relative to the calendar year. | | |
| Setting | 5 | Describe the setting, locations, and relevant dates, including periods of recruitment, exposure, follow-up, and data collection | Methods paragraph 1 | See below |
|  |  | The ROS, MAP, and MARS studies are prospective observational cohort studies of the risk factors for the common chronic disease of aging. They share nearly identical protocols. The ROS is a longitudinal study of aging in Catholic brothers, nuns, and priests from across the United States [22]. The MAP is a community-based study of aging in the greater Chicago area that enrols participants with diverse backgrounds and socioeconomic status from continuous care retirement communities throughout north-western Illinois, as well as from individual homes across the Chicago metropolitan area [23]. Both the ROS and MAP cohorts consist primarily of individuals of self-reported European descent (93%). The MARS is a community-based study of older adults of self-reported African descent in the greater Chicago area [24]. Participants in all three studies are free of known dementia at study enrolment. All participants in the ROS and MAP cohorts and a subset of participants in the MARS cohort agree to brain donation upon death. For our primary analyses, we included all ROS, MAP, and MARS participants with complete cognitive testing (see below) and without cognitive impairment at the time of their first cognitive assessment. Of the 4004 ROS, MAP, and MARS participants enrolled between January 1994 and May 2017, 3924 completed the entire cognitive battery at their baseline assessment, 2761 of whom were without cognitive impairment. Cognitive, demographic, and clinical data from these 2761 participants were included in our primary analyses. In addition, structured autopsies were performed on 1410 decedents, and RNA-sequencing data from the dorsolateral prefrontal cortex were obtained from 507 of these. The SDS (www.clinicaltrials.gov registration NCT01800214) is an observational cohort study of consecutively collected cases (1992-2014) from a tertiary care clinic in Toronto, Canada [25]. For this study, we studied participants clinically classified as having Alzheimer’s disease at their baseline assessment (see criteria below), and in whom Dementia Rating Scale (DRS) scores were available. Of 1143 participants enrolled between 1992 and 2014, 285 had a primary clinical diagnosis of Alzheimer’s disease, of whom 271 had DRS scores at the time of baseline assessment. Clinical, demographic, and psychometric data from these 271 participants were included in this study. The CNC cohort consists of patients who attended a clinical and research memory clinic specializing in the care of patients with cognitive disorders, the memory clinic at the Lariboisiere Hospital, between July 2, 2008 and June 15, 2017, who had undergone CSF collection with measurement of amyloid and tau, who had undergone CSF collection with measurement of amyloid and tau, whose entire clinical work-up was performed at the Lariboisiere hospital, and for whom a final clinical diagnosis was available. Of 2298 patients assessed between July 2008 and June 2017, the vast majority had had a portion of their clinical work-up completed at an outside institution and were excluded from these analyses. Of the remaining 424 participants with a final clinical diagnosis, 321 also had a lumbar puncture with quantification of Aβ40, Aβ42, Tau, and phospho-Tau. Psychometric, clinical, demographic, and cerebrospinal fluid data from these 321 participants were included in this study. | | |
| Participants | 6 | (*a*) *Cohort study*—Give the eligibility criteria, and the sources and methods of selection of participants. Describe methods of follow-up  *Case-control study*—Give the eligibility criteria, and the sources and methods of case ascertainment and control selection. Give the rationale for the choice of cases and controls  *Cross-sectional study*—Give the eligibility criteria, and the sources and methods of selection of participants | Methods paragraph 1 | See above |
|  |  |  |  |  |
|  |  | (*b*) *Cohort study*—For matched studies, give matching criteria and number of exposed and unexposed  *Case-control study*—For matched studies, give matching criteria and the number of controls per case | NA | NA |

| Variables | | 7 | Clearly define all outcomes, exposures, predictors, potential confounders, and effect modifiers. Give diagnostic criteria, if applicable | | | Methods paragraphs 3-5, 19 | See below |
| --- | --- | --- | --- | --- | --- | --- | --- |
|  | |  | The main outcomes of interest were cognitive performance considered as a continuous variable, clinical dementia diagnosis considered as a categorical variable, levels of CSF AD biomarkers (including Aβ42, Aβ40, tau, and phospho-tau) considered as continuous variables, and dorsolateral prefrontal cortex expression of GENCODE v14 genes, considered as continuous variables. The main predictor of interest was date relative to the calendar year (of cognitive assessment for the cognitive outcomes, of lumbar puncture for CSF markers, and of death for post-mortem RNA-sequencing) considered as an angular continuous variable. Potential confounders included age (at time of cognitive evaluation, lumbar puncture or death) considered as a continuous variable, sex considered as a dichotomous variable, years of education considered as a continuous variable, number of depressive symptoms considered as a continuous variable, hours of sleep considered as a continuous variable, hours of physical activity considered as a continuous variable, serum thyroid stimulating hormone levels as a continuous variable, and clock time of assessment or death considered as a categorical variable in 1-hour bins. Potential effect modifiers included age (continuous), sex (dichotomous), and race (categorical).  Participants in the ROS, MAP, and MARS cohorts underwent annual uniform structured cognitive evaluations consisting of a battery of 19 cognitive tests spanning 5 domains (episodic memory [logical Memory Ia, logical Memory IIa, immediate story recall, delayed story recall, word list memory, word list recall, word list recognition], semantic memory [Boston naming test, category fluency, national adult reading test], working memory [digit span forward, digit span backward, digit ordering], perceptual speed [symbol digit modalities test, number comparison, Stroop word reading, Stroop color naming], and visuospatial ability [judgment of line orientation, standard progressive matrices). As described previously [26], a composite measure for each domain was created by converting each test within each domain to a z-score and averaging the z-scores. The five domain composite measures were then averaged to create a composite global cognitive score scaled such that 0 represents the mean score of all participants at baseline, positive scores indicate better performance, and 1 unit represents approximately 1 standard deviation of performance. Participants were classified as having a clinical diagnosis of dementia by National Institute of Neurological And Communicative Disorders and Stroke-Alzheimer’s Disease and Related Disorders Association criteria [27], Persons with cognitive impairment by neuropsychological testing but without a clinical diagnosis of dementia were classified as having mild cognitive impairment (MCI). Participants without dementia or MCI were classified as having no cognitive impairment (NCI). Date of cognitive assessment was recorded at the time of cognitive assessment.  Patients in the SDS cohort underwent a comprehensive battery of 17 tests as described previously [28]. Of these, data from the Dementia Rating Scale [29], the California Verbal Learning Test [30], digit span forward and backward [31], the Digit Symbol Substitution Test [32], the Wisconsin Card Sort Test [33], semantic fluency for animals [34, 35], and the Benton Line Orientation Test [36] were included in these analyses. Alzheimer’s disease diagnoses were made by consensus of two independent clinicians according to prevailing clinical consensus criteria [37]. Date of cognitive assessment was recorded at the time of cognitive assessment.  Patients in the CNC cohort underwent routine clinical, neurological, and neuropsychological evaluations, and brain imaging as clinically indicated. On the basis of all available data, including CSF biomarkers, and in accordance with NIA-ADRDA criteria [38], patients were classified into two groups: AD and non-AD. Complex cases were discussed, and diagnoses were made by a multidisciplinary team of neurologists, geriatricians, and neuropsychologists. Non-AD subjects included subjects with cognitive disorders other than AD including frontotemporal dementia, Lewy body disease, Parkinson’s disease, Creutzfeldt-Jakob disease, and non-degenerative dementia (including vascular dementia, alcohol-related dementia, normal pressure hydrocephalus, infectious disease and psychiatric disorders among others). | | | | |
| Data sources/ measurement | | 8* | For each variable of interest, give sources of data and details of methods of assessment (measurement). Describe comparability of assessment methods if there is more than one group | | | Methods paragraphs 1, 6-16 | See below |
|  | | |  | **Assessment of Clinical Covariates**  In the ROS, MAP, and MARS cohorts, we computed age at the time of cognitive assessment form the self-reported date of birth and date of assessment. We computed age at death from the self-reported date of birth and the date of death. We recorded sex and self-reported race at the time of the baseline interview. Depressive symptoms were assessed on the date of cognitive evaluation with a 10-item version of the Center for Epidemiologic Studies Depression (CES-D) scale [25]. Clock time of cognitive testing was recorded at the time of testing. Times were binned into hours, and considered as a categorical variable. For sleep duration, MAP and ROS participants were asked on the date of cognitive evaluation to report how many hours they usually slept at night over the preceding month. For physical activity, MAP and ROS participants were asked on the date of cognitive evaluation to report the number of hours per week spent in 5 categories of activities: walking, gardening or yard work, calisthenics or general exercise, bicycle riding, and swimming or water exercises [26]. In a subset of ROS, MAP, and MARS participants, serum thyroid stimulating hormone levels were assessed by immunoassay by Quest Diagnostics (New Jersey, United States).  In the SDS cohort, age, sex, and years of education were extracted from clinical records.  In the CNC cohort, age and sex were extracted from clinical records.  **Evaluation of Cerebrospinal Fluid**  In the CNC cohort, lumbar punctures were performed on fasting patients within one month following their clinical diagnosis, usually between 09:00 and 12:00. Within 4 hours of collection, samples were centrifuged at 1,000 g for 10 minutes at 4oC, aliquoted to 0·5-ml polypropylene tubes, and stored at -80oC. Subsequently, CSF Aβ40, Aβ42, total tau, and p-tau were measured using a commercially available sandwich enzyme-linked immunosorbent assay (INNOTEST, Fujirebio Europe NV, Gent, Belgium) according to the manufacturer’s instructions. Date of lumbar puncture was recorded at the time of cognitive assessment.  **Neuropathological Evaluation**  Neuropathological evaluation was performed in decedents from the ROS, MAP, and MARS cohorts. Alzheimer’s disease pathology was quantified as described previously [40, 41]. Neurofibrillary tangles, diffuse plaques, and neuritic plaques were visualized by Bielschowsky silver staining in sections from the frontal, temporal, parietal, and entorhinal cortices and the hippocampus. For a categorical pathological diagnosis of Alzheimer’s disease, cases were classified as no Alzheimer’s disease, low likelihood Alzheimer’s disease, intermediate likelihood Alzheimer’s disease or high likelihood Alzheimer’s disease based on the National Institutes of Aging (NIA)-Reagan criteria [42]; a participant was considered to have a pathological diagnosis of Alzheimer’s disease if their NIA-Reagan classification was “intermediate likelihood” or “high likelihood”. To generate a composite continuous measure of the burden of Alzheimer’s disease pathology, neurofibrillary tangles, diffuse plaques, and neuritic plaques were counted in the regions above, the raw counts were divided by the standard deviation of the same index for that region across the entire cohort, and the scaled scores were averaged as described previously [41]. [28]. In addition to the above, the percent area occupied by amyloid beta was quantified as previously described [30]. Briefly, after immersion fixation in paraformaldehyde, tissue blocks from 8 brain regions (mid-frontal cortex, premotor cortex, inferior temporal cortex, angular gyrus, calcarine cortex, anterior cingulate cortex, entorhinal cortex, and hippocampus CA1/subiculum) were embedded in paraffin, sectioned, and incubated with antibodies to amyloid beta (monoclonal mouse anti-amyloid beta clone 10D5, Elan Pharmaceuticals, San Francisco) followed by development with diaminobenzidine and nickel. The mean % area occupied by amyloid beta immunopositive material was quantified in each of the 8 brain regions in each individual using a systematic random sampling approach and a multistage computational image analysis protocol as previously described [30]. These values were averaged across regions to generate a summary measure of amyloid load for each individual.  To quantify Lewy body pathology, 6 micron paraffin-embedded sections from the cingulate, entorhinal, midfrontal, middle temporal, and inferior parietal cortices and the substantia nigra were immunostained with antibodies to alpha synuclein (pSyn-64; 1:20,000; Wako Chemical USA Inc; Richmond, VA). The distribution of Lewy bodies was graded on a semi-quantitative scale (0 = none, 1 = brainstem or limbic predominant, 2 = neocortical) according to a modified version of published criteria [43].  Macroscopic cerebral infarcts were identified by visual inspection of 1cm coronal slabs, and confirmed by histological review as previously described [44]. Microscopic infarcts were quantified in a minimum of 9 regions (6 cortical and 3 subcortical) as previously described [45].  **Evaluation of Dorsolateral Prefrontal Cortex Transcript Expression**  RNA sequencing was performed on blocks of dorsolateral prefrontal cortex from a subset of ROS and MAP participants as previously described [8]. RNA was extracted from dorsolateral prefrontal cortex blocks and quantified by Nanodrop (Thermo Fisher Scientific, Waltham, MA). An Agilent Bioanalyzer was used to assess quality. Samples from which less than 5ug of RNA were obtained, or samples with a RNA integrity (RIN) score of 5 or less, were excluded from further analysis. The strand-specific dUTP method [46] with poly-A selection [47] was used by the Broad Institute Genomics Platform to prepare the RNA sequencing library. Poly-A selection was followed by first strand specific cDNA synthesis, with dUTP used for second strand specific cDNA synthesis, followed by fragmentation and Illumina adapter ligation for library construction. An Illumina HiSeq machine was used to perform sequencing with 101 bp paired-end reads, achieving a coverage of 150M reads for the first 12 samples, which served as a deep coverage reference. The remaining samples were sequenced with coverage of 50M reads. Next, beginning and ending low quality bases and adapter sequences were trimmed from the reads, and ribosomal RNA reads were removed. The Bowtie 1 software package [48] was used to align the trimmed reads to the reference genome. Finally, the RSEM software packing was used to estimate, in units of fragments per kilobase per million mapped fragments (FPKM), expression levels for 55,889 individual GENCODE v14 genes, which were then quantile-normalized, correcting for batch effect with Combat [36]. These data are available through the synapse.org AMP-AD Knowledge Portal (www.synapse.org SynapseID syn3388564).  We used a consensus clustering methods called SpeakEasy [49] to robustly define coexpressed molecular systems based on the gene-gene correlation matrix. We then calculated the average expression level across all genes in each module.  Data from 507 samples meeting quality control criteria as above, and with full clinical data, were included in this analysis (Table 1). | | | |
| Bias | | 9 | Describe any efforts to address potential sources of bias | | | Discussion paragraph 6 | See below |
|  | |  | … Of particular note, as all of the study cohorts were based in relatively northern latitudes (north of 41^o^N) it is possible that extremes of weather, particularly cold, may have resulted in participants with greater degrees of neurological disability (e.g. those with greater cognitive impairment) being less likely to be able to attend study visits in the winter. However, this would have biased the study toward finding a lower likelihood of meeting diagnostic criteria for MCI or AD in the coldest months, which is the opposite of the effect that was seen. | | | | |
| Study size | | 10 | Explain how the study size was arrived at | | | Methods paragraph 1 | See response to item 5 above |

| Quantitative variables | 11 | Explain how quantitative variables were handled in the analyses. If applicable, describe which groupings were chosen and why | Methods paragraph 19 | See below |
| --- | --- | --- | --- | --- |
|  |  | The main outcomes of interest were cognitive performance considered as a continuous variable, combined clinical diagnosis of MCI or dementia considered as a categorical variable, levels of CSF AD biomarkers (including Aβ42, Aβ40, tau, and phospho-tau) considered as continuous variables, and dorsolateral prefrontal cortex expression of GENCODE v14 genes, considered as continuous variables. The main predictor of interest was date relative to the calendar year (of cognitive assessment for the cognitive outcomes, of lumbar puncture for CSF markers, and of death for post-mortem RNA-sequencing) considered as an angular continuous variable. Potential confounders included age (at time of cognitive evaluation, lumbar puncture or death) considered as a continuous variable, sex considered as a dichotomous variable, years of education considered as a continuous variable, number of depressive symptoms considered as a continuous variable, hours of sleep considered as a continuous variable, hours of physical activity considered as a continuous variable, serum thyroid stimulating hormone levels as a continuous variable, and clock time of assessment or death considered as a categorical variable in 1-hour bins. Potential effect modifiers included age (continuous), sex (dichotomous), and race (categorical). | | |
| Statistical methods | 12 | (*a*) Describe all statistical methods, including those used to control for confounding | S1 Methods | See below |
|  |  | The main outcomes of interest were cognitive performance considered as a continuous variable, combined clinical diagnosis of MCI or dementia considered as a categorical variable, levels of CSF AD biomarkers (including Aβ42, Aβ40, tau, and phospho-tau) considered as continuous variables, and dorsolateral prefrontal cortex expression of GENCODE v14 genes, considered as continuous variables. The main predictor of interest was date relative to the calendar year (of cognitive assessment for the cognitive outcomes, of lumbar puncture for CSF markers, and of death for post-mortem RNA-sequencing) considered as an angular continuous variable. Potential confounders included age (at time of cognitive evaluation, lumbar puncture or death) considered as a continuous variable, sex considered as a dichotomous variable, years of education considered as a continuous variable, number of depressive symptoms considered as a continuous variable, hours of sleep considered as a continuous variable, hours of physical activity considered as a continuous variable, serum thyroid stimulating hormone levels as a continuous variable, and clock time of assessment or death considered as a categorical variable in 1-hour bins. Potential effect modifiers included age (continuous), sex (dichotomous), and race (categorical).  In the ROS, MAP, and MARS cohorts, we characterized seasonal patterns in cognition by considering cognition as a function of date of evaluation, adjusted for age at evaluation, sex, and level of education as follows:   (1)  where E(C) is the expected composite global cognitive function, d is the date of assessment, As is the amplitude [peak to trough difference] of seasonal rhythmicity, ϕs is the acrophase [date of peak cognition] of seasonal rhythmicity, and β_1_x_1_ … β_n_x_n_ are the covariates age, sex, and years of education.. For these analyses, the seasonal period was fixed at 365 days. This is a limitation of any study design where each individual contributes only 1 data point to 365-day sampling period. All dates were converted to radians (2π radians = 365 days; 0 radians = January 1) for analysis and then converted back to calendar dates for the purposes of visual representation.  For computational efficiency, we fit equivalent linearized models of the form   (2)  and As, and ϕs from equation (1) were calculated using the formulae   (3)   (4)  We calculated standardized amplitudes by dividing the amplitude from equation (3) by the standard deviation of composite global cognitive function across the cohort at baseline. Moreover, we contextualized the magnitude of this amplitude by comparing it to the magnitude of the effect estimate for age from equation (1). We determined 95% confidence intervals on both the standardized amplitude and amplitude relative to age by bootstrapping over 1000 iterations. Amplitudes were considered negative if more than pi/2 radians out of phase from the un-resampled data. Moreover, to obtain empiric p-values for the estimates of seasonal amplitude relative to age effects, we generated 1000 permuted null datasets by shuffling dates of assessment, estimated the amplitude of seasonal rhythmicity relative to the effect of age for each null dataset, and then computed the proportion of these null datasets for which the ratio was greater than seen in our observed data. To quantify the contribution of seasonal rhythmicity to the model fit in equation (2), we compared the residual sum of squares for equation (2) to that of a reduced model without the terms for seasonal rhythmicity (5)  and determined the F-statistic   (6)  and associated 2-sided p-value with α=0.05. The greater the contribution of seasonal rhythmicity to the overall model fit, the greater the value of Fs. To examine for effect modification by age, we augmented equation (2) with multiplicative interaction terms between age and the cosine and sine terms from equation 2 and compared this augmented model with equation (2), generating an F-statistic and p-value. We then repeated this for sex, self-reported race, and source cohort.  Using this approach, in our primary analyses, we assessed seasonal rhythmicity in composite global cognitive function at the baseline evaluation in the ROS and MAP cohorts, controlling for age, sex, and level of education, and including only those participants without MCI or dementia. We then did the same for the MARS cohort. Then, we analyzed the ROS, MAP, and MARS cohorts together, controlling for the effect of study cohort.  Cognition may be affected by a number of potential confounders that may also show seasonal variation, including time of cognitive testing, depression, hours of sleep, and physical activity. To account for these, in the combined ROS, MAP, and MARS cohorts, we considered models adjusted for time of cognitive testing (considered as a categorical variable by hour), number of depressive symptoms (considered as a continuous variable), hours of sleep (considered as a continuous variable), and hours spent engaged in physical activities (considered as a continuous variable).  Next we examined whether seasonal rhythms in cognition may vary by specific cognitive domain. To do so, in the combined ROS, MAP, and MARS cohorts, we repeated our primary analyses, considering separately summary scores in the specific domains of working memory, perceptual speed, visuospatial function, semantic memory, and episodic memory.  Seasonal variation in cognition may plausibly lead to seasonal variation in the diagnosis of mild cognitive impairment or dementia. To assess this, we followed the ROS, MAP and MARS participants from our primary analyses above, and considered their clinical diagnosis at the last available cognitive evaluation. We considered the odds of being classified as having MCI or dementia as a function of the date of assessment, adjusted for age, sex, education, and source cohort:   (7)  To quantify the contribution of seasonal rhythmicity to the log odds of being classified as having MCI or dementia, we compared fit of equation (7) to a reduced equation without the seasonal terms using a likelihood ratio test, with 2-sided p-value α=0.05:   (8)  We also considered season as a categorical variable, defining winter/spring as January-June and summer/fall as July-December, and considered the effect of categorical season on the log odds of being diagnosed with MCI/dementia, comparing to a reduced equation without the seasonal terms suing a likelihood ratio test, with 2-sided p-value α=0.05:   (9)  Next, we examined whether Alzheimer’s disease pathology is associated with differential seasonal rhythmicity of cognition. To do so, we repeated our primary analyses above using the last known cognitive assessments from deceased ROS, MAP, and MARS participants with and without Alzheimer’s disease pathology, defined as an NIA-Reagan score of intermediate or higher, at death. To do so, we considered an augmented model, allowing for independent effects of AD pathology on the level and rhythmicity of cognition:   (10)  To quantify the contribution of AD pathology to level of cognition, we used the F-test to compare equation (10) to a reduced model without the linear term for AD pathology, with a 2-sided p-value α=0.05:   (11)  To quantify the contribution of AD pathology to the rhythmicity of cognition, we compared equation (10) to a reduced model without the terms for the AD effect on rhythmicity, with a 2-sided p-value α=0.05:   (12)  Next, we repeated our primary analyses in the SDS cohort, with the total Dementia Rating Scale score as the primary outcome, and then with the Dementia Rating Scale subscores, as well as scores on the digit span test, Symbol Digit Substitution test, California Verbal Learning Test, Semantic Fluency (animal naming) test, Wisconsin Card Sort Test, and Benton Line Orientation Test as secondary outcomes, all considered as continuous variables.  Pathophysiological processes underlying Alzheimer’s disease, including amyloid and tau metabolism, are important contributors to impaired cognition in older adults. To examine for evidence of seasonal rhythmicity in amyloid and tau biology, we examined CSF Aβ40, Aβ 42 tau, and phospho-tau in the CNC cohort as a function of date of lumbar puncture, adjusted for age and sex, using an identical approach as used for cognition above, considering first all participants together, adjusted for diagnosis (AD vs. non-AD) similar to equations 2 and 4 above, and then allowing for differences in level and rhythmicity between participants with and without AD, similar to equations 10, 11, and 12 above but with CSF biomarker levels rather than cognition as the outcomes.  Next, we set out to identify co-expressed molecular systems that may be related to seasonal rhythms in cognition. We considered a set of co-expressed genes potentially related to seasonal rhythms in cognition if it fulfilled all the of the following: 1) it was seasonally rhythmic, 2) it was either in phase or antiphase to the rhythm of cognition and 3) it was associated with cognitive performance proximate to death. To assess seasonal rhythmicity, we considered the mean expression level of each gene module as a function of date and time of death, adjusted for age at death, sex, level of education, and methodological covariates such as batch, post-mortem interval, and RNA quality (RIN score), and accounting for multiple comparisons by permutation, as described previously.[8] A module was considered seasonally rhythmic if its p-value, adjusted for multiple comparisons, was <0.05. To assess association with cognition proximate to death, we considered the composite global cognition proximate to death as a linear function of mean module expression, adjusted for age, sex, education, and methodological covariates including postmortem interval. Batch, and RNA quality (RIN score), while accounting for multiple comparisons by generation of 10,000 null datasets created by permuting module labels. A module was considered associated with cognition if its p-value adjusted for multiple comparisons was <0.05.  For co-expression modules meeting the above criteria, we controlled for potential confounders including depression and neuropathologies potentially affecting cognition including the burden of Alzheimer’s disease pathology considered as a continuous measure, the presence or absence of cortical Lewy bodies considered as a dichotomous variable, and the number of macroscopic and microscopic cortical infarcts considered as continuous variables, by repeating the above analysis in models adjusted for these covariates. We also examined the impact of Alzheimer’s disease pathology on the level and rhythmicity of module expression by considering augmented models similar to the analysis we performed with cognition in equations 10, 11, and 12 above.  We next examined for associations between module expression and other cognition-related phenotypes including the individual cognitive domains, Mini-Mental state examination scores, and measures of AD pathology by calculating Spearman correlations between mean module expression at death, and either cognitive phenotypes at the last measurement proximate to death, or neuropathological phenotypes at death, adjusting for multiple comparisons by Bonferroni correction.  Finally, we examined for transcription factor binding sites associated with the 4 co-expression modules putatively associated with seasonal rhythms of cognition. To do so, we used genome-wide annotated binding sites for 161 transcription factors from the ENCODE project [13, 50, 51]. We considered a transcript to be locally associated with a transcription factor if its transcription start sites overlapped with one of the transcription factor’ ENCODE-annotated binding sites, or was within 2000bp of it. For each of the four co-expression modules, we then used logistic regression models of the form   (13)  to examine the independent association of the local presence of binding sites for each of the 161 ENCODE transcription factors with the odds of a transcript being a member of a given co-expression module. We corrected for multiple comparisons using Bonferroni correction for 161 transcription factors (α=0.05/161=0.0003). | | |
|  |  | (*b*) Describe any methods used to examine subgroups and interactions | S1 Methods | See below |
|  |  | To examine for effect modification by age, we augmented equation (2) with multiplicative interaction terms between age and the cosine and sine terms from equation 2 and compared this augmented model with equation (2), generating an F-statistic and p-value. We then repeated this for sex, self-reported race, and source cohort.  …  Next, we examined whether Alzheimer’s disease pathology is associated with differential seasonal rhythmicity of cognition. To do so, we repeated our primary analyses above using the last known cognitive assessments from deceased ROS, MAP, and MARS participants with and without Alzheimer’s disease pathology, defined as an NIA-Reagan score of intermediate or higher, at death. To do so, we considered an augmented model, allowing for independent effects of AD pathology on the level and rhythmicity of cognition:   (10)  To quantify the contribution of AD pathology to level of cognition, we used the F-test to compare equation (10) to a reduced model without the linear term for AD pathology, with a 2-sided p-value α=0.05:   (11)  To quantify the contribution of AD pathology to the rhythmicity of cognition, we compared equation (10) to a reduced model without the terms for the AD effect on rhythmicity, with a 2-sided p-value α=0.05:   (12)  …  Pathophysiological processes underlying Alzheimer’s disease, including amyloid and tau metabolism, are important contributors to impaired cognition in older adults. To examine for evidence of seasonal rhythmicity in amyloid and tau biology, we examined CSF Aβ40, Aβ 42 tau, and phospho-tau in the CNC cohort as a function of date of lumbar puncture, adjusted for age and sex, using an identical approach as used for cognition above, considering first all participants together, adjusted for diagnosis (AD vs. non-AD) similar to equations 2 and 4 above, and then allowing for differences in level and rhythmicity between participants with and without AD, similar to equations 10, 11, and 12 above but with CSF biomarker levels rather than cognition as the outcomes.  …  For co-expression modules meeting the above criteria, we controlled for potential confounders including depression and neuropathologies potentially affecting cognition including a continuous measure of Alzheimer’s disease pathology, the presence or absence of cortical Lewy bodies, and the number of macroscopic and microscopic cortical infarcts by repeating the above analysis in models adjusted for these covariates. We also examined the impact of Alzheimer’s disease pathology on the level and rhythmicity of module expression by considering augmented models similar to the analysis we performed with cognition in equations 10, 11, and 12 above. | | |
|  |  | (*c*) Explain how missing data were addressed | Methods paragraph 18 | See below |
|  |  | For all analyses, only observations with complete data for each analysis were used. | | |
|  |  | (*d*) *Cohort study*—If applicable, explain how loss to follow-up was addressed  *Case-control study*—If applicable, explain how matching of cases and controls was addressed  *Cross-sectional study*—If applicable, describe analytical methods taking account of sampling strategy | NA | NA |
|  |  | (*e*) Describe any sensitivity analyses | NA | NA |
| Results | | | | |
| Participants | 13* | (a) Report numbers of individuals at each stage of study—eg numbers potentially eligible, examined for eligibility, confirmed eligible, included in the study, completing follow-up, and analysed | Methods paragraph 1 | See response to item 5 above. |
|  |  | (b) Give reasons for non-participation at each stage | Methods paragraph 1 | See response to item 5 above |
|  |  | (c) Consider use of a flow diagram | NA | NA |
| Descriptive data | 14* | (a) Give characteristics of study participants (eg demographic, clinical, social) and information on exposures and potential confounders | Table 1 | Table 1 |
|  |  | (b) Indicate number of participants with missing data for each variable of interest | Methods paragraph 1 | See response to item 5 above |
|  |  | (c) *Cohort study*—Summarise follow-up time (eg, average and total amount) | Results paragraph 3 | See below |
|  |  | We hypothesized that the seasonal impact on cognition would be reflected in rates of cognitive diagnoses. To test this, we analyzed the last available assessment for ROS, MAP, and MARS participants (Table 1 Column 4; a median [IQR] of 6.0 [3.0 to 11.0] years since study enrolment), and used logistic regression models adjusted for age, sex, education, and source cohort to model the impact of assessment date on the odds of meeting criteria for MCI or AD. | | |
| Outcome data | 15* | *Cohort study*—Report numbers of outcome events or summary measures over time | Results paragraphs 2-5 | See below |
|  |  | The ROS and MAP are longitudinal cohort studies of aging in which participants are free of dementia at study enrollment and agree to brain donation upon death. The mean (SD) baseline composite global cognitive score among participants without cognitive impairment in the combined ROS and MAP cohort was 0.26 (0.44) units.  (…)  In the African-American MARS cohort (Table 1 Column 2; N=527), the mean (SD) composite global cognitive score was 0.19 (0.47).  (…)  Of the 2761 participants, 813 (29.5%) met diagnostic criteria for MCI or AD.  (…)  To assess generalizability, we replicated our analyses in baseline data from the Canadian SDS cohort, an observational study of cases from a tertiary care memory clinic in Toronto, Canada. The mean (SD) Mattis Dementia Rating Scale score (DRS) of participants with Alzheimer’s disease was 115 (17).  (,,,)  To test for an impact of season on amyloid and tau biology, we analyzed CSF amyloid and tau levels in patients with (n=176) and without (n=145) clinical AD from the CNC cohort, an observational cohort study of patients from a tertiary care memory center in Paris, France (Table 1 Column 7; N=321). The mean (SD) CSF Aβ42, Aβ40, tau, and phopho-tau, were 754 (280), 11835 (4856), 469 (318), and 71 (40) pg/ml, respectively. |  |  |
|  |  | *Case-control study—*Report numbers in each exposure category, or summary measures of exposure | NA | NA |
|  |  | *Cross-sectional study—*Report numbers of outcome events or summary measures | NA | NA |
| Main results | 16 | (*a*) Give unadjusted estimates and, if applicable, confounder-adjusted estimates and their precision (eg, 95% confidence interval). Make clear which confounders were adjusted for and why they were included | Results paragraphs 1-9 | See below |
|  |  | In an unadjusted model, season had a significant association with composite global cognitive function, with the highest average composite global cognition seen just before the fall equinox (p=0.004). Compared to participants assessed at the winter/spring trough, the composite global cognitive score of participants assessed at the summer/fall peak was 0.20 standard deviations (SD) higher [95% CI 0.09 to 0.32] p=0.004. In a model adjusted for age, sex, and education, the effect of season on composite global cognition was similar, with a peak before the fall equinox (Fig 1A). Compared to participants assessed in the winter and spring, the composite global cognitive score of participants assessed in the summer and fall was 0.13 standard deviations higher [95% CI 0.04 to 0.24] p=0.039 – equivalent to that of participants 4.4 years younger [95% CI 1.8 to 8.6] p=0.018. In the African-American MARS cohort (Table 1 Column 2; N=527), the mean (standard deviation) composite global cognitive score was 0.19 (0.47). In the MARS cohort, in a model adjusted for age, sex, and education, the magnitude of the seasonal was similar was similar to the ROS and MAP cohorts, with 0.21 standard deviations of composite global cognition [95% CI 0.06 to 0.47] p=0.11 separating participants evaluated at the winter/spring nadir and summer/fall acrophase, equivalent to an age difference of 4.3 years [95% CI 1.29 to 9.26] p=0.064. The p-value in the MARS cohort was somewhat attenuated, although this may reflect the smaller number of MARS (n=527) compared to ROS+MAP participants (n=2234) in these analyses. Pooling data across all three cohorts (Table 1 Column 3) enhanced the effect (Fig 1C, amplitude=0.14 standard deviations [95% CI 0.07 to 0.23], p=0.0066, with the peak to trough difference equivalent in effect to a 4.8 year difference in age [95% CI 2.1 to 8.5] p=0.009).  (…)  We hypothesized that the seasonal impact on cognition would be reflected in rates of cognitive diagnoses. To test this, we analyzed the last available assessment for ROS, MAP, and MARS participants (Table 1 Column 4; a median [IQR] of 6.0 [3.0 to 11.0] years since study enrolment), and used logistic regression models adjusted for age, sex, education, and source cohort to model the impact of assessment date on the odds of meeting criteria for MCI or dementia. Of the 2761 participants, 813 (29.5%) met diagnostic criteria for MCI or AD. In unadjusted models, the effect of season on diagnosis was significant (deviance=-10.3, p=0.006). A participant evaluated in the winter or spring (January-June) had a roughly 24% higher odds of meeting criteria for MCI or dementia than one evaluated in the summer or fall (July-December; OR 1.24, 95% CI 1.05-1.47, p=0.008). In models adjusted for age, sex, and education, the effects were similar (Fig 2A; deviance=-10.2, p=0.006). In these adjusted models, a participant evaluated in the winter or spring (January-June) had a roughly 30% higher odds of meeting criteria for MCI or dementia than one evaluated in the summer or fall (July-December; OR 1.31, 95% CI 1.10-1.57, p=0.003).  (…)  To assess generalizability, we replicated our analyses in baseline data from the Canadian SDS cohort, an observational study of cases from a tertiary care memory clinic in Toronto, Canada. The mean (SD) Mattis Dementia Rating Scale score (DRS) of participants with Alzheimer’s disease was 115 (17). In a model adjusted for age, sex, and years of education, the impact of season on the total DRS score was significant (amplitude 0.50SD [95% CI 0.07 to 0.66], F=4.13, p=0.02; Fig 2C). The highest DRS scores were seen in the fall, with an average difference of eight points between participants evaluated in the spring and fall.  (…)  Considering all participants together, in a model adjusted for age and sex, CSF Aβ42 levels were strongly rhythmic, peaking in late June (amplitude 0.30SD [95% CI 0.10 to 0.64]; F=6.01; p=0.003; Fig 3A). There was a trend toward rhythmicity in Aβ40 (F-2.80; p=0.06; Fig 3C). Tau, and phospho-Tau were not rhythmic (F=0.67 and p=0.515 for Tau; F=0.40, p=0.67 for pTau; Fig 3, E and G).  (…)  Of 56 modules examined, in models adjusted for age, sex, education, time of death, postmortem interval, and methodological variables like batch and RNA quality (RIN) score, four modules (labeled arbitrarily m6: n=328 genes, amplitude=0.44SD [95%CI 0.21 to 0.65], F=6.24, p=0.0021; m13: n=353 genes, amplitude=0.46SD [95% CI 0.27 to 0.76], F=7.15, p=0.00087; m109: n=390 genes, amplitude=0.43SD [95% CI 0.24 to 0.67], F=6.25, p=0.0021; and m122: n=370 genes, amplitude 0.46SD [95% CI 0.20 to 0.71], F=6.84, p=0.0012) met all three criteria (S2 Table).  (…)  Finally, we used genome-wide annotated binding sites for 161 transcription factors from the ENCODE project [13] to identify sites associated with each module. After correction for multiple comparisons by permutation, fifteen, thirteen, six, and nine transcription factor binding sites were associated with membership in modules m6, m13, m109, and m122 respectively (S5 Table). Of these, five (BCL11A, EGR1, and THAP1 associated with m109; and CTCF and MEF2A associated with m13) were themselves previously identified as seasonally rhythmic [8]. | | |
|  |  | (*b*) Report category boundaries when continuous variables were categorized | NA | NA |
|  |  | (*c*) If relevant, consider translating estimates of relative risk into absolute risk for a meaningful time period | NA | NA |

Continued on next page

| Other analyses | 17 | Report other analyses done—eg analyses of subgroups and interactions, and sensitivity analyses | Results paragraphs 1-9 | See below |
| --- | --- | --- | --- | --- |
|  |  | The impact of season on cognition did not vary by age, sex, race, or source cohort (p<0.05 for interaction terms) and was independent of cognitive test time, depression, sleep, and physical activity (S1 Table). The timing of rhythms of cognitive performance was similar across cognitive subdomains (S1 Figure) with peak performance near the fall equinox. However, the seasonal effect was strongest for working memory and perceptual speed and weaker for the other domains.  (…)  Participants with pathological AD had lower mean composite global cognition than those without (estimate=-0.65SD, SE=0.05, p=1.2e-33); however, the seasonal rhythmicity of cognition did not differ significantly between these groups (F=0.22, p=0.81), suggesting preserved seasonality of cognition in pathological AD.  (…)  There were strong effects on the DRS subdomains of attention, speed, and initiation, and weaker effects on other domains (S2 Figure and S3 Figure).  (..)  Patients with AD had lower CSF Aβ42, and higher Aβ40, Tau and phospho-Tau, as expected (Fig 3B, D F, and H). Moreover, the rhythmicity of Aβ42 was attenuated in participants with AD (Fig 3B).  (…)  These modules remained associated with season and cognition in models adjusted for depression and other neuropathologies (S4 Table). They were also associated with other cognitive and neuropathological phenotypes (Fig 5). Module rhythmicity was similar in those with and without AD pathology (p>0.05 for all four modules). | | |
| Discussion | | | | |
| Key results | 18 | Summarise key results with reference to study objectives | Discussion paragraph 1 | See below |
|  |  | In this study of 3353 older adults across multiple countries, cohorts and races, season had a significant and reproducible impact on cognition, with peak cognition near the fall equinox and a seasonal effect equivalent to a roughly four-year difference in age. This was independent of mood, sleep, and physical activity; it was clinically significant as reflected in a nearly 30% higher odds of meeting criteria for MCI or dementia in winter and spring compared to summer and fall; and it persisted in cases with pathologically confirmed AD. Moreover, season also had an impact on CSF Aβ levels, and the brain expression of cognition-associated gene modules, which were associated with characteristic transcription factor binding sites. | | |
| Limitations | 19 | Discuss limitations of the study, taking into account sources of potential bias or imprecision. Discuss both direction and magnitude of any potential bias | Discussion paragraph 6 | See below |
|  |  | Some limitations should be noted. First, each subject or sample contributed only one data point per annual cycle. It would be preferable to derive individual-level estimates of rhythmicity by repeated testing or sampling from the same individual throughout the year. However, to limit participant burden, we limited evaluations to once per year and CSF sampling to once per participant. Moreover, it would not be ethically permissible to repeatedly sample neocortical tissue from living participants. Second, we relied on self report to measure environmental and behavioral factors leading up to each evaluation, and had limited quantitative data, making it difficult to distinguish the influence of these factors. Of particular note, as all of the study cohorts were based in relatively northern latitudes (north of 41oN) it is possible that extremes of weather, particularly cold, may have resulted in participants with greater degrees of neurological disability (e.g. those with greater cognitive impairment) being less likely to be able to attend study visits in the winter. However, this would have biased the study toward finding a lower likelihood of meeting diagnostic criteria for MCI or dementia in the coldest months, which is the opposite of the effect that was seen. Third, we had limited information on cause of death in decedents, which may influence gene expression at time of death, and which may be seasonal. Fourth, although this was one of the largest studies to date to examine seasonal rhythms of human brain gene expression, it nevertheless lacked statistical power to draw firm conclusions at the individual gene level, hence our analysis of gene sets defined by co-expression patterns. Fifth, this study was consisted primarily of individuals of self-described European or African descent living in temperate regions of the northern hemisphere, limiting generalizability to other races or geographic locations. It would be of particular interest to study populations in temperate regions of the southern hemisphere, where one might expect seasonal effects to be in antiphase to those in the northern hemisphere, and to study populations in equatorial regions, where one might expect seasonal effects to be relatively attenuated compared to temperate regions. Finally, all cognitive and CSF assessments were performed during a relatively limited window within the 24-hour day –during daylight hours. While this limited the potential for confounding by time of assessment, it also limited the capacity to obtain robust estimates of circadian effects, which would require sampling around the 24-hour clock. | | |
| Interpretation | 20 | Give a cautious overall interpretation of results considering objectives, limitations, multiplicity of analyses, results from similar studies, and other relevant evidence | Discussion paragraphs 2-8 | See below |
|  |  | Several previous studies found no seasonal impact on cognition [3, 6, 7] while others showed impact on some domains but not others [4, 48], or only in individuals with psychopathology [5]. In contrast, we demonstrated a robust impact of season on cognition in older adults with and without AD in multiple cohorts. Several factors may account for this divergence from previous studies. First, several studies examined cognition only near the solstices [4, 6], or correlated cognition to day length [7]. If, as our data suggest, cognition peaks near the fall equinox, these approaches would fail to uncover significant seasonality. Second, several studies did not examine working memory [6, 7], which in our data was the cognitive domain most affected by season. Third, it is possible that in younger adults, who were the subject of most previous studies, an abundance of cognitive reserve minimized the impact of season, whereas this effect becomes more important as cognitive reserve diminishes with cognitive aging. One study that found no seasonal impact on cognition in young adults nevertheless found an impact on cortical fMRI responses during a working memory task [3], with peak responses occurring near the fall equinox, roughly coincident with the peak in working memory observed in our study. This supports the hypothesis of a subclinical seasonal impact on cognition in younger adults, seen on fMRI but not cognitive testing.  The persistence of a robust summer and fall peak in cognition suggests that even in pathologically confirmed AD, there remains substantial cognitive plasticity. Identifying drivers or mediators of this effect may enable leveraging this plasticity to improve cognition year-round. There are several potential hypotheses. First, the summer and fall peak in cognition may be driven by environmental factors such as light and temperature. If true, then interventions such as phototherapy or temperature modification may be effective in sustaining this year-round. Second, behavioral factors such as activity [49], sleep [50], or diet [51] show seasonality and may drive the summer and fall peak in cognition. In this study, the seasonal impact on cognition was independent of self-reported sleep and physical activity, although studies incorporating objective markers of these and other behaviors may reveal a more important role for behavioral factors. Third, seasonal rhythms in psychological (e.g. depression) state may drive the seasonal impact on cognition. In this study, the seasonality of cognition was independent of depression; however, other psychological factors, such as negative affect, which has been associated with MCI and dementia [52], may be important. While data on positive and negative affect were collected on some of our participants, they were available on too few participants to test their impact on seasonal rhythms of cognition in this study. Fourth, seasonal rhythms in physiological state may potentially drive the seasonal impact on cognition. In our study, adjusting for serum levels of thyroid stimulating hormone did not substantially attenuate estimates of the seasonal effect on cognition. However, additional metabolic factors that may potentially link season to cognition are Vitamin D [53], sex hormones like testosterone [54], and melatonin [55]. Unfortunately, data on serum levels of vitamin D, testosterone, and melatonin were not available in our study. Finally, an endogenous seasonal clock may drive rhythms in cognition. Supporting this, there is some evidence for such a clock in other species [56], and seasonal rhythms in fMRI responses to cognitive tasks persist even with control of behavioral and environmental factors [3]. Studies combining serial measurements of cognition, environment, behavior, and physiology in the same individuals at multiple time points across the year will help to distinguish these possibilities.  In the CNC cohort, CSF Aβ42 levels were highest in the summer, slightly preceding the time of peak cognition and the lowest risk for MCI/dementia in the ROS, MAP and MARS cohorts. This was unrelated to the clock time of LP as all LPs were performed within a narrow 2-3 hour window each day. The phase difference between the CSF rhythms in the CNC cohort and the cognitive and molecular rhythms in the other cohorts may relate in part to differences in latitude (CNC 49oN vs. south of 44oN for other cohorts). The overlap between the timing of greatest CSF Aβ42 levels and lowest odds of MCI/dementia are in keeping with existing literature showing that high CSF Aβ42 levels are associated with better cognition even in older adults without a clinical diagnosis of Alzheimer’s disease [57]. Other studies have identified diurnal rhythms of CSF amyloid [45], but to our knowledge, seasonal rhythms have not been described. These rhythms may reflect seasonal variation in Aβ42 synthesis, or in transitions between compartments (parenchyma, insterstitium, CSF, systemic circulation), or forms (monomeric, oligomeric, and insoluble) of amyloid. CSF monomeric Aβ42 concentrations are inversely related to brain interstitial oligomeric [58] and fibrillar [59] Aβ in some contexts. Therefore, low winter CSF monomeric Aβ42 levels may reflect high levels of brain interstitial oligomeric Aβ species, with which may have an adverse impact on cognition [60]. Studies examining the impact of season on oligomeric Aβ species may shed light on this.  Irrespective of the ultimate drivers of the summer and fall peak in cognition, efforts to understand its neurobiological substrates may identify molecular pathways whose plasticity is preserved in pathological AD, and which may therefore be promising targets to enhance cognitive function in AD. In this study, the summer and fall peak in cognition paralleled the seasonal nadirs of two prefrontal cortex co-expression gene modules (m6 and m109) negatively associated with cognition, and the seasonal acrophases of two gene modules (m13 and m122) positively associated with cognition. These phase relationships mean that the seasonal effects of these modules may be synergistic; peak cognition in the summer and fall may be driven by a synergy of higher expression of modules positively associated with cognition and negatively associated with Aβ (m13 and m122) and lower expression of modules negatively associated with cognition and positively associated with Aβ (m6 and m109) while in the winter and spring the opposite occurs. Gene ontology and cell type enrichment analyses for these modules have previously been published [39]. Although three of the four modules (m6, m13, and m122) are not strongly enriched for specific gene ontology pathways or cell types, m109 is relatively enriched for pathways involved in chromatin modification and cell cycle control, and is associated with cognitive decline and amyloid pathology [39]. In experiments in human cell lines, knock-down of selected m109 genes, including INPPL1 and PLXNB1, results in reduced extracellular amyloid levels, potentially linking seasonal rhythms of m109 to seasonal rhythms of brain extracellular amyloid [39]. Thus one possible scenario is that in the summer and early fall, neocortical expression of m109 and associated genes such as INPPL1 and PLXNB1 are at their lowest, leading to relatively lower brain interstitial oligomeric Aβ species (reflected by relatively high CSF monomeric Aβ) leading to improved cognition, while in the winter and early spring the opposite is true (S6 Fig). Additional work is needed to examine the cognitive impact of increasing or decreasing expression of these modules in model systems, and on understanding the mechanisms, such as the transcription factors identified in this study, that account for their rhythmicity. This may enable therapeutically increasing the expression of modules 13 and 122, or therapeutically decreasing the expression of modules 109 and 6 to enhance cognition year-round in individuals with AD.  Some limitations should be noted. First, each subject or sample contributed only one data point per annual cycle. It would be preferable to derive individual-level estimates of rhythmicity by repeated testing or sampling from the same individual throughout the year. However, to limit participant burden, we limited evaluations to once per year and CSF sampling to once per participant. Moreover, it would not be ethically permissible to repeatedly sample neocortical tissue from living participants. Second, we relied on self report to measure environmental and behavioral factors leading up to each evaluation, and had limited quantitative data, making it difficult to distinguish the influence of these factors. Of particular note, as all of the study cohorts were based in relatively northern latitudes (north of 41oN) it is possible that extremes of weather, particularly cold, may have resulted in participants with greater degrees of neurological disability (e.g. those with greater cognitive impairment) being less likely to be able to attend study visits in the winter. However, this would have biased the study toward finding a lower likelihood of meeting diagnostic criteria for MCI or dementia in the coldest months, which is the opposite of the effect that was seen. Third, we had limited information on cause of death in decedents, which may influence gene expression at time of death, and which may be seasonal. Fourth, although this was one of the largest studies to date to examine seasonal rhythms of human brain gene expression, it nevertheless lacked statistical power to draw firm conclusions at the individual gene level, hence our analysis of gene sets defined by co-expression patterns. Fifth, this study was consisted primarily of individuals of self-described European or African descent living in temperate regions of the northern hemisphere, limiting generalizability to other races or geographic locations. It would be of particular interest to study populations in temperate regions of the southern hemisphere, where one might expect seasonal effects to be in antiphase to those in the northern hemisphere, and to study populations in equatorial regions, where one might expect seasonal effects to be relatively attenuated compared to temperate regions. Finally, all cognitive and CSF assessments were performed during a relatively limited window within the 24-hour day –during daylight hours. While this limited the potential for confounding by time of assessment, it also limited the capacity to obtain robust estimates of circadian effects, which would require sampling around the 24-hour clock.  This study also had several strengths. Notwithstanding the population and geographic limitations noted above, we replicated our primary analyses in cohorts from 3 different countries, and 2 different racial groups, enhancing generalizability. Second, the temporal convergence of cognitive and molecular data enhanced biological plausibility and allowed us to draw links between molecular and behavioral rhythms. Third, cognitive assessments, LPs, and dates of death were spread throughout the year, rather than limited to discrete sampling times, maximizing the capacity to detect rhythmicity irrespective of phase. Fourth, we had histopathological confirmation in decedents, providing a high degree of diagnostic certainty.  Overall, we report robust estimates of an important source of variation in cognitive performance and its biological correlates among a diverse set of older individuals. Season should be considered as an important confounder when analyzing data from therapeutic trials and observational studies in AD. In particular, studies aiming to estimate rates of cognitive decline with repeated measurements of the same participant should either assess the same individual at the same date in different study cycles (as is done in the ROS, MAP, and MARS cohorts) or should consider season as a covariate. In clinical practice seasonal effects may be an important source of diagnostic misclassification and may account for the observation that some individuals with MCI revert to normal cognition on subsequent testing. Our findings should also inform the design of clinical algorithms that leverage the winter and spring cognitive nadir to enhance sensitivity in identifying subjects at the earliest stages of disease. Finally, the effect sizes seen in this study suggest that targeting environmental or behavioral drivers of seasonal cognitive plasticity, or the key transcription factors and genes identified in this study as potentially mediating these effects, may allow us to substantially improve cognition in adults with and without Alzheimer’s disease pathology. | | |
| Generalisability | 21 | Discuss the generalisability (external validity) of the study results | Discussion paragraph 6 | See below |
|  |  | … this study was consisted primarily of individuals of self-described European or African descent living in temperate regions of the northern hemisphere, limiting generalizability to other races or geographic locations, particularly equatorial regions (…) Notwithstanding the population and geographic limitations noted above, we replicated our primary analyses in cohorts from 3 different countries, and 2 different racial groups, enhancing generalizability. | | |
| Other information | |  | | |
| Funding | 22 | Give the source of funding and the role of the funders for the present study and, if applicable, for the original study on which the present article is based | Online form | See below |
|  |  | This study was funded by National Institutes of Health (www.nih.gov) P30AG10161, R01AG052488, R01AG043379, R01AG15819, R01AG17917, R01AG36042, R01AG36836, U01AG046152, RF1AG022018, R01AG042210, and R01NS078009; Canadian Institutes of Health Research (http://www.cihr-irsc.gc.ca/), MOP125934, and MSH136642; Alzheimer’s Association (www.alz.org) and Brain Canada (www.braincanada.ca) Grant AARG501466; National Sciences and Engineering Research Council of Canada (www.nserc-crsng.gc.ca) grant RGPIN-2017-0692; Agence Nationale de la Recherche (www.agence-nationale-recherche.fr) MALZ grant 2013; Agence Publique Hopitaux de Paris (http://fondationrechercheaphp.fr/); Institut National de la Sante et de la Recherche Medicale (www.inserm.fr/en); the Illinois Department of Public Health (http://www.dph.illinois.gov/); the Alzheimer Society of Canada (alzheimer.ca/); the Heart and Stroke Foundation of Canada (http://www.heartandstroke.ca/); and the Robert C. Borwell Endowment Fund.  “The funders had no role in study design, data collection and analysis, decision to publish, or preparation of the manuscript.” | | |

*Give information separately for cases and controls in case-control studies and, if applicable, for exposed and unexposed groups in cohort and cross-sectional studies.

**Note:** An Explanation and Elaboration article discusses each checklist item and gives methodological background and published examples of transparent reporting. The STROBE checklist is best used in conjunction with this article (freely available on the Web sites of PLoS Medicine at http://www.plosmedicine.org/, Annals of Internal Medicine at http://www.annals.org/, and Epidemiology at http://www.epidem.com/). Information on the STROBE Initiative is available at www.strobe-statement.org.
