# Supplementary material for: Seasonal plasticity of cognition and related biological measures in adults with and without Alzheimer disease: Analysis of multiple cohorts
Source: PLoS Med. 2018 Sep 4;15(9):e1002647. doi: 10.1371/journal.pmed.1002647 (PMC6122787; doi:10.1371/journal.pmed.1002647)
Supplement: S1 Table — (DOCX) [file pmed.1002647.s009.docx]

**S1 Table: Association Between Season and Composite Global Cognitive Function in Adults Without Alzheimer’s Disease – Consideration of Potential Confounders at Baseline Assessment**

| **Potential**  **Confounder** | **Cohorts**  **Analyzed** | **Participants with Complete Data** | **Model** | **Adjusted For** | **Amplitude** | **Acrophase (radians)** | **F** | **P-value** |
| --- | --- | --- | --- | --- | --- | --- | --- | --- |
| Clock Time of Testing  (Hour) | ROSMAPMARS | 2600 | base | age+sex+education+source cohort | 0·036 | 4.1 | 6.1 | 0·002 |
|  |  |  | adjusted | base+test time | 0·036 | 4.1 | 6.0 | 0·005 |
| Depressive Symptoms  (Number) | ROSMAPMARS | 2761 | base | age+sex+education+source cohort | 0.032 | 4.1 | 4.9 | 0.007 |
|  |  |  | adjusted | base+depression | 0.030 | 4.1 | 4.5 | 0·011 |
| Sleep  (Hours) | ROSMAP | 2233 | base | age+sex+education+source cohort | 0.028 | 4.0 | 3.2 | 0.042 |
|  |  |  | adjusted | base+sleep hours | 0.028 | 4.0 | 3·2 | 0·042 |
| Physical Activity  (Hours) | ROSMAP | 2234 | base | age+sex+education+source cohort | 0.028 | 4.0 | 3.2 | 0.042 |
|  |  |  | adjusted | base+physical activity | 0·030 | 4.0 | 3.7 | 0·026 |
| Thyroid Stimulating  Hormone (U/mL) | MARS | 512 | base | age+sex+education | 0.049 | 4.3 | 2.2 | 0.11 |
|  |  |  | adjusted | base+TSH | 0.054 | 4.4 | 2.5 | 0.08 |
