# Supplementary material for: Seasonal plasticity of cognition and related biological measures in adults with and without Alzheimer disease: Analysis of multiple cohorts
Source: PLoS Med. 2018 Sep 4;15(9):e1002647. doi: 10.1371/journal.pmed.1002647 (PMC6122787; doi:10.1371/journal.pmed.1002647)
Supplement: S2 Table — (DOCX) [file pmed.1002647.s010.docx]

**S2 Table: Association Between Season (Winter/Spring vs. Summer/Fall) and Odds of Meeting Criteria for MCI or Dementia – Consideration of Potential Confounders at time of Last Available Cognitive Assessment.**

| **Potential**  **Confounder** | **Cohorts**  **Analyzed** | **Participants with Complete Data** | **Model** | **Adjusted For** | **Odds Ratio (Winter/Spring vs. Summer/Fall)** | **95% CI** | **P-value** |
| --- | --- | --- | --- | --- | --- | --- | --- |
| Clock Time of Testing  (Hour) | ROSMAPMARS | 2686 | base | age+sex+education+source cohort | 1.31 | 1.09-1.57 | 0.003 |
|  |  |  | adjusted | base+test time | 1.30 | 1.09-1.56 | 0.004 |
| Depressive Symptoms  (Number) | ROSMAPMARS | 2605 | base | age+sex+education+source cohort | 1.36 | 1.12-1.64 | 0.001 |
|  |  |  | adjusted | base+depression | 1.34 | 1.11-1.61 | 0.003 |
| Sleep  (Hours) | ROSMAP | 2233 | base | age+sex+education+source cohort | 1.29 | 1.11-1.49 | 0.001 |
|  |  |  | adjusted | base+sleep hours | 1.28 | 1.10-1.49 | 0·001 |
| Physical Activity  (Hours) | ROSMAP | 2234 | base | age+sex+education+source cohort | 1.28 | 1.11-1.49 | 0.001 |
|  |  |  | adjusted | base+physical activity | 1.27 | 1.09-1.48 | 0.002 |
| Thyroid Stimulating  Hormone (U/mL) | MARS | 512 | base | age+sex+education | 1.70 | 1.00-2.91 | 0.05 |
|  |  |  | adjusted | base+TSH | 1.73 | 1.02-2.97 | 0.04 |
