# Supplementary material for: Seasonal plasticity of cognition and related biological measures in adults with and without Alzheimer disease: Analysis of multiple cohorts
Source: PLoS Med. 2018 Sep 4;15(9):e1002647. doi: 10.1371/journal.pmed.1002647 (PMC6122787; doi:10.1371/journal.pmed.1002647)
Supplement: S3 Table — Based on ROS, MAP, and MARS participants with available DLPFC RNA-Seq data (n = 507). Acrophase in radians. **In acrophase column: within 2 months of acrophase or nadir of cognition. Amplitude in standard units of composite global cognition. p-Value for rhythmicity adjusted for multiple comparisons by permutation. **In p-value for rhythmicity (adjusted) column: adjusted p-value < 0.05. p-Value for association with cognition adjusted for multiple comparisons by permutation. **In p-value for association with cognition (adjusted) column: p < 0.05. (DOCX) [file pmed.1002647.s011.docx]

**S3 Table: Association Between Season and the Expression of Cognition-Associated Molecular Systems.** Based on ROS, MAP, and MARS participants with available dorsolateral prefrontal cortex RNA-seq data (N=507). Acrophase in radians. ** indicates within 2 months of acrophase or nadir of cognition. Amplitude in standard units of composite global cognition. P-value for rhythmicity adjusted for multiple comparisons by permutation. ** indicates adjusted p-value <0·05. P-value for association with cognition adjusted for multiple comparisons by permutation. ** indicates p<0·05.

| **Module** | **Acrophase** | **Amplitude** | **F-Statistic for Rhythmicity** | **P-Value**  **For Rhythmicity**  **(unadjusted)** | **P-Value for Rhythmicity (adjusted)** | **Estimated Effect on Cognition (per 1SD difference)** | **P-Value for Association with Cognition (unadjusted)** | **P-Value for Association with Cognition (adjusted)** |
| --- | --- | --- | --- | --- | --- | --- | --- | --- |
| m1 | 2·7 | 0·12 | 1·9 | 0·151 | 0·86 | 0·02 | 0·603 | >0·999 |
| m2 | **3·49 | 0·14 | 2·5 | 0·083 | 0·686 | 0·03 | 0·478 | >0·999 |
| m3 | 2·8 | 0·13 | 2·43 | 0·089 | 0·706 | 0·04 | 0·313 | >0·999 |
| m4 | **1·30 | 0·12 | 1·86 | 0·158 | 0·87 | -0·07 | 0·093 | 0·989 |
| m5 | **0·68 | 0·15 | 3·05 | 0·048 | 0·519 | -0·18 | <0·001 | **0·001 |
| m6 | **0·81 | 0·23 | 7·06 | 0·001 | **0·025 | -0·17 | <0·001 | **0·003 |
| m7 | **0·57 | 0·16 | 3·27 | 0·039 | 0·456 | -0·22 | <0·001 | **<0·001 |
| m8 | **1·47 | 0·12 | 1·94 | 0·144 | 0·848 | -0·11 | 0·012 | 0·448 |
| m9 | **1·89 | 0·05 | 0·33 | 0·718 | >0·999 | -0·09 | 0·055 | 0·93 |
| m10 | **1·38 | 0·11 | 1·53 | 0·217 | 0·938 | -0·08 | 0·07 | 0·964 |
| m11 | **1·16 | 0·04 | 0·2 | 0·815 | >0·999 | -0·17 | <0·001 | **0·003 |
| m12 | 3·11 | 0·16 | 3·2 | 0·041 | 0·473 | 0·15 | <0·001 | **0·016 |
| m13 | **3·37 | 0·23 | 7·23 | 0·001 | **0·021 | 0·19 | <0·001 | **<0·001 |
| m14 | 2·44 | 0·1 | 1·2 | 0·302 | 0·98 | 0·12 | 0·005 | 0·22 |
| m16 | **1·80 | 0·04 | 0·23 | 0·791 | >0·999 | 0·03 | 0·429 | >0·999 |
| m17 | **0·81 | 0·19 | 4·43 | 0·012 | 0·206 | -0·05 | 0·254 | >0·999 |
| m18 | **1·81 | 0·04 | 0·23 | 0·791 | >0·999 | 0·06 | 0·186 | >0·999 |
| m19 | 2·52 | 0·09 | 1·07 | 0·344 | 0·989 | 0·05 | 0·195 | >0·999 |
| m21 | 2·88 | 0·07 | 0·71 | 0·49 | 0·999 | 0·08 | 0·055 | 0·929 |
| m22 | **1·83 | 0·07 | 0·51 | 0·599 | >0·999 | 0·1 | 0·018 | 0·577 |
| m23 | **3·23 | 0·1 | 1·42 | 0·242 | 0·955 | 0·15 | <0·001 | **0·018 |
| m106 | **4·13 | 0·13 | 1·89 | 0·152 | 0·862 | 0·03 | 0·456 | >0·999 |
| m107 | 5·33 | 0·11 | 1·46 | 0·233 | 0·949 | -0·01 | 0·72 | >0·999 |
| m108 | 6 | 0·09 | 1·09 | 0·337 | 0·988 | -0·09 | 0·037 | 0·832 |
| m109 | **0·24 | 0·22 | 6·44 | 0·002 | **0·041 | -0·23 | <0·001 | **<0·001 |
| m110 | 6·13 | 0·08 | 0·77 | 0·461 | 0·998 | -0·14 | 0·001 | **0·026 |
| m111 | 5·75 | 0·13 | 2·27 | 0·105 | 0·757 | -0·1 | 0·013 | 0·469 |
| m112 | 5·7 | 0·06 | 0·41 | 0·661 | >0·999 | -0·05 | 0·195 | >0·999 |
| m113 | 5·64 | 0·12 | 1·77 | 0·171 | 0·889 | -0·03 | 0·428 | >0·999 |
| m114 | **0·14 | 0·19 | 4·88 | 0·008 | 0·147 | -0·14 | 0·001 | **0·030 |
| m115 | 5·74 | 0·09 | 1·07 | 0·343 | 0·988 | -0·05 | 0·195 | >0·999 |
| m116 | **0·28 | 0·13 | 2·11 | 0·122 | 0·804 | -0·05 | 0·203 | >0·999 |
| m117 | **0·10 | 0·13 | 2·23 | 0·108 | 0·768 | 0·01 | 0·789 | >0·999 |
| m118 | 5·4 | 0·09 | 1·07 | 0·342 | 0·988 | -0·03 | 0·453 | >0·999 |
| m119 | 6·04 | 0·06 | 0·56 | 0·573 | >0·999 | -0·02 | 0·717 | >0·999 |
| m121 | **4·37 | 0·16 | 3·15 | 0·044 | 0·489 | 0·06 | 0·155 | >0·999 |
| m122 | **3·81 | 0·24 | 7·48 | 0·001 | **0·017 | 0·17 | <0·001 | **0·004 |
| m123 | **4·57 | 0·11 | 1·46 | 0·234 | 0·95 | 0·12 | 0·006 | 0·251 |
| m125 | **3·82 | 0·11 | 1·66 | 0·192 | 0·914 | 0·14 | 0·001 | 0·054 |
| m126 | **3·90 | 0·03 | 0·1 | 0·907 | >0·999 | 0·15 | 0·001 | **0·028 |
| m127 | **3·61 | 0·19 | 4·97 | 0·007 | 0·136 | 0·19 | <0·001 | **<0·001 |
| m128 | **3·76 | 0·13 | 2·49 | 0·084 | 0·688 | 0·17 | <0·001 | **0·004 |
| m131 | 3·18 | 0·1 | 1·22 | 0·297 | 0·978 | 0·17 | <0·001 | **0·002 |
| m187 | **1·94 | 0·03 | 0·11 | 0·898 | >0·999 | 0·1 | 0·016 | 0·523 |
| m233 | **3·44 | 0·06 | 0·43 | 0·653 | >0·999 | 0·11 | 0·009 | 0·355 |
| m234 | 0·03 | 0·06 | 0·47 | 0·628 | >0·999 | -0·02 | 0·685 | >0·999 |
| m257 | **3·58 | 0·22 | 6·41 | 0·002 | **0·042 | 0·13 | 0·003 | 0 |
