# Supplementary material for: Seasonal plasticity of cognition and related biological measures in adults with and without Alzheimer disease: Analysis of multiple cohorts
Source: PLoS Med. 2018 Sep 4;15(9):e1002647. doi: 10.1371/journal.pmed.1002647 (PMC6122787; doi:10.1371/journal.pmed.1002647)
Supplement: S4 Table — (DOCX) [file pmed.1002647.s012.docx]

**S4 Table: Genes Contained in Modules 6, 13, 109, and 122.**

| **Module** | **Genes** |
| --- | --- |
| m6 | 7SK, ABCA6, ABCA7, ABCC10, AC004893.11, AC005306.3, AC018730.1, AC034193.5, AC084018.1, AC092143.1, AC093668.3, AC093838.4, AC103965.1, ACSS2, ACTN4, ADAM33, ADAMTS13, AF038458.5, AGAP3, AIP, AL136419.6, ALS2CR12, ANKRD11, AP000347.4, AP1G2, APBA3, ASPRV1, ATAD3C, ATF6B, ATG4B, ATP13A1, AUP1, AVIL, BANF1, BCAR1, C17orf28, C17orf67, C19orf44, C1QTNF6, C20orf96, C3orf62, C4orf48, C8orf73, CACTIN, CARD9, CARS2, CCDC11, CCDC120, CCDC144C, CCDC151, CCDC34, CCT3, CDC25B, CDK10, CDK11A, CEACAM19, CHD1, CHURC1-FNTB, CIZ1, CLASRP, CLDN15, CLDN9, CLEC18A, CNIH4, CNKSR1, COASY, COG4, COL21A1, COL6A1, COX19, CPSF3L, CPT1B, CREBRF, CTA-963H5.5, CTB-134H23.1, CTB-31O20.2, CTC-425O23.2, CTC-479C5.10, CTC-479C5.16, CTC-503J8.6, CTRL, CTSA, CXXC1, CYHR1, DDX11L2, DFFA, DFFB, DHFR, DNAJB2, DNAJC21, DOM3Z, DPH5, DPP9, DUSP18, E2F4, EEF1DP2, EIF2C3, ELAVL3, ELMOD3, ENTHD2, FAM167B, FAM179A, FAM50A, FAM89B, FANCL, FAP, FASTKD1, FBRS, FCHO1, FGF17, FIZ1, FLII, GDPD3, GIT1, GKAP1, GLTSCR1, GNRH1, GPS2, GS1-393G12.12, GSDMB, HAUS5, HDAC10, HELZ, HIGD2A, HIST1H4H, HMGB3P22, HOOK2, HSD11B2, HSD17B1, HSF1, HSF4, IFT140, IMPDH1, INTS9, IP6K2, JRK, KAT2A, KAT8, KEAP1, KIAA0913, KIAA0930, KIAA1551, KIF27, KLC4, KLHL17, KLKB1, KPTN, LPAR2, LRP5L, LRRC37B, LY6G5C, LZTR1, MACROD1, MAF1, MAFIPL, MAGED4B, METTL17, MFAP1P1, MIER2, MITD1, MLXIP, MLXIPL, MMP23B, MORC2, MORN5, MPZ, MRPS5, MUC20, N4BP1, NAA60, NBPF20, NBPF8, NBR2, NCKAP5L, NEIL1, NKAPL, NMT2, NOP2, NPFF, NPIPP1, NPR2, NR2F1, NRIP2, NSUN5P1, NUP62, OSBPL7, PACS1, PARP16, PCED1A, PI4KAP1, PI4KB, PLA2G4B, PLEKHM1, PLK1, PLK5, PLOD3, POLE, POLN, POLR2H, POLR2J4, PPARD, PPP1R10, PRKCSH, PRPF31, PRSS53, PTK7, PTOV1, PURG, QRICH2, RBM22P2, RBM48, RCN3, RFNG, RFX1, RNASEH2C, RNF216P1, RP11-108P20.1, RP11-147I3.1, RP11-157P1.4, RP11-192H23.4, RP11-192H23.8, RP11-196G11.2, RP11-223I10.1, RP11-296I10.6, RP11-311C24.1, RP11-318C24.2, RP11-334C17.5, RP11-340F14.5, RP11-355O1.11, RP11-395P17.3, RP11-440L14.1, RP11-459F3.6, RP11-465L10.7, RP11-46D6.1, RP11-46F15.2, RP11-487E1.1, RP11-498D10.6, RP11-552E20.3, RP11-561I11.2, RP11-57A19.3, RP11-582J16.5, RP11-58B17.1, RP11-611E13.2, RP11-61A14.2, RP11-635N19.1, RP3-406A7.7, RP4-791M13.3, RP5-995J12.2, RP6-109B7.3, RP9, RP9P, RPAP1, RPL19P12, RPPH1, RPS20P33, RSAD1, SAYSD1, SCXA, SDHAP1, SFTPC, SIRT7, SLC25A41, SLC43A1, SLCO3A1, SLMO1, SMARCD3, SMG5, SMPD4, SNRPA, SOLH, SOX15, SPRY1, SRRT, ST3GAL4, STK38, SULT1A3, SYNE2, TACC3, TAF3, TAZ, TBC1D17, TCAP, THAP4, TMCC2, TMEM214, TNFRSF25, TOP3B, TP53I3, TPD52L2, TPM2, TRAF7, TRIP4, TRMT1, TSEN54, TTC28-AS1, TTLL7, TUBA3FP, TYRO3, UBE2L3, UBXN7, UCKL1, UPF2, USP21, VARS2, WASH4P, WASH6P, WDR53, WDR83OS, WFIKKN1, WHSC2, XXbac-B461K10.4, YPEL3, ZBTB16, ZBTB44, ZC3H18, ZDHHC11, ZGLP1, ZMYND8, ZNF141, ZNF276, ZNF511, ZNF582, ZNF587, ZNF609, ZNF638, ZNF692, ZNF761, ZRSR1, |
| m13 | AAGAB, AARSD1, ABCA11P, ABCF3, ABLIM3, AC007246.3, AC012309.5, AC023490.2, AC069154.2, ACAD9, ACBD6, ACP2, ACTR1B, ADC, AIG1, AKR1A1, ALKBH3, AP1M1, AP4B1, APBB3, APEH, ARL16, ARSG, ARX, ASAH2B, ASB8, ASH2L, ASMTL, ASS1, ATAT1, ATF7IP2, ATPIF1, B9D1, BDH1, BEX2, BEX5, BIVM, BRE, BRF2, BZRAP1-AS1, BZW2, C14orf101, C14orf45, C15orf57, C17orf108, C17orf49, C19orf70, C1orf204, C22orf39, C2orf15, C3orf26, C6orf57, C7orf63, C8orf59, C9orf123, CBR3-AS1, CBWD7, CCDC115, CCDC13, CCDC96, CCNH, CDC40, CEP57, CHAF1B, CHCHD6, CHID1, CHML, CLYBL, CNNM4, COG1, COL12A1, COQ3, CRMP1, CTD-2162K18.5, CTD-2228K2.5, CTD-2517M22.14, CTNNBIP1, CWF19L1, CXorf56, CYP2C8, CYP2E1, DAK, DALRD3, DCTN1-AS1, DCUN1D2, DDX25, DDX28, DEM1, DEPDC5, DHDDS, DHRS11, DHX33, DHX35, DIRAS3, DNAJC5G, DNTTIP1, DOPEY2, DPH1, DUSP11, E2F3, EAPP, EID2B, EIF3K, ELAC2, ELP6, EML4, EXOG, FAF1, FAM149A, FAM153A, FAM160A2, FAM174A, FAM47E, FAM5B, FAM86DP, FANK1, FARSB, FASTKD3, FBXO16, FEN1, FGF9, FIBP, FOPNL, FOXRED1, FUCA1, G6PD, GABPB1, GALT, GARNL3, GGNBP2, GLOD4, GPAA1, GPX3, GRIK1, GRIK2, GRIPAP1, GSS, GSTA4, GUF1, HABP4, HACL1, HAUS2, HDDC3, HERC6, HPS6, HRASLS, ICA1, IKBKG, IMP4, IPO9, KCTD13, KIAA0895L, KIAA1984, KRT17P1, KRT17P2, LETMD1, LIAS, LIN52, LINC00339, LINC00515, LINC00665, LL22NC03-86G7.1, LMLN, LNP1, LRPPRC, MANBAL, MAP3K12, MDP1, ME3, MECR, MED6, MIR22HG, MORN1, MORN3, MPPED2, MRPL2, MRPL54, MRPS25, MRPS9, MSH3, MSTO1, MT-CO3, MTPAP, NDUFA10, NDUFAF5, NIT2, NLE1, NLN, NME5, NME9, NOL6, NRSN2, NUBPL, NUDT18, NUDT2, NUP210, OR7E154P, PA2G4, PACRG, PARK2, PARN, PARP2, PCYOX1L, PCYT2, PDE7A, PEBP1, PHF7, PIBF1, PIGZ, PIH1D1, PKD1P1, PMS1, PMS2P3, PMS2P4, PNMA3, POLR3F, PPARGC1A, PPIE, PPIH, PRPF6, PSEN2, PSMB2, PSMC3, PSMG1, PSPC1, PTGES3L, PTRH1, RAB37, RABEPK, RABL2A, RARS, RBAK, RBFA, RBM11, RBM45, RFC2, RFC5, RFXAP, RHOV, RP1-63G5.5, RP11-151N17.2, RP11-197K6.1, RP11-321E2.3, RP11-388C12.8, RP11-400F19.6, RP11-413E6.1, RP11-446H18.3, RP11-469M7.1, RP11-509E16.1, RP11-53O19.1, RP11-689P11.2, RP11-698N11.4, RP11-72I8.1, RP11-886D15.1, RP11-890B15.3, RP4-565E6.1, RP5-837J1.2, RP5-902P8.10, RP6-65G23.3, RRN3P1, RSPH1, RSRC1, RTN4IP1, RUFY2, RWDD2B, SDHAF1, SDSL, SEC14L5, SETD4, SFTPD, SIDT1, SIRT3, SLC25A26, SLC26A4, SLC35A1, SLC37A3, SLC4A1AP, SLX4, SNHG15, SNX14, SPAG7, SPATA7, SPEF2, SRSF8, SSSCA1, SST, STX16-NPEPL1, STX5, STXBP5-AS1, SULT1A1, SUPT16H, SYCE1, TARBP1, TBC1D22A, TBC1D24, TBP, TCEAL5, TCEAL6, TCERG1, TDP1, TFB1M, THUMPD3, TIMM10, TIMM23B, TIRAP, TMEM14B, TMEM17, TMEM9, TMSB15B, TOE1, TPBGL, TRAF5, TRANK1, TRAP1, TRAPPC2L, TRAPPC9, TSNAX, TSPYL2, TSPYL5, TSR2, TSSC1, TTC21B, TTC8, TTLL1, TUBG2, UBLCP1, UBXN11, USP11, USP43, UTP11L, VIPAS39, WBP2, WDR67, WDR86, WDR89, XXbac-B135H6.15, XXYLT1, YLPM1, YWHAE, ZBBX, ZNF200, ZNF223, ZNF337, ZNF425, ZNF441, ZNF529, ZNF540, ZNF544, ZNF554, ZNF629, ZNF707, ZNF720, ZNF890P, |

| m109 | A2ML1, ABL1, AC135178.1, ACIN1, ADORA2A, AK4, AKT1, AL133458.1, ANAPC16, ANKRD40, APLN, ARAP1, ARHGAP5, ARHGEF10L, ARHGEF40, ARID1A, ARL5A, B3GAT2, BAHCC1, BCAM, BCL2L1, BEND7, BLM, BRD7, BRD8, BTBD7, C10orf114, C14orf93, C22orf32, C22orf46, C6orf3, C9orf114, CAB39L, CALCOCO1, CARHSP1, CASC3, CCDC40, CCDC85C, CDA, CDK2AP1, CDKN2C, CEP104, CEP112, CEP350, CERS4, CGN, CHKB-CPT1B, CHST11, CLHC1, CLIP2, CMBL, CNNM3, CNOT6, COG2, COL5A3, COL9A1, CPSF2, CRB2, CREBBP, CRK, CSK, CSRP1, CTB-171A8.1, CTC-205M6.2, CTC-428G20.3, CTD-2380F24.1, CTNS, CXCR4, CYP20A1, DANCR, DAXX, DDIT4, DDIT4L, DDX11, DENND2A, DHX38, DLL1, DNASE1, DOT1L, DSTNP2, DVL2, EED, EHMT1, ELN, ENTPD3-AS1, EPN2, ERMAP, EZR, FAM118A, FAM193A, FAM43A, FAM48A, FAM59A, FAM59B, FAM63A, FAM65C, FBXL7, FBXO2, FLCN, FMNL3, FOXJ1, FOXN3, FOXO4, FTSJ3, FUT11, FYCO1, FZD8, GAL3ST4, GALM, GDF11, GIGYF2, GIPR, GJC1, GNG7, GPER, HBP1, HCFC1, HDAC1, HDGF, HEY2, HIBCH, HIGD1B, HMBOX1, HMG20B, HOMER3, HPS4, hsa-mir-6723, HSPB2, IDI2-AS1, IGFBP5, IKBKB, IL17RB, ILVBL, ING5, INPP5K, INPPL1, IQCE, ITGB5, ITPK1, KANK2, KANSL1, KAT6A, KATNAL2, KCNJ10, KCNJ16, KCTD11, KCTD18, KHSRP, KIAA1009, KIF13A, KIF1C, KIF5B, KLF15, KLHL24, KLHL36, KRBA2, KRCC1, KRT8P12, KTN1, LAMB2, LETM1, LINC00263, LINC00499, LRCH3, LRP5, LRRC58, LSS, MAF, MAML1, MAPK7, MAPKAP1, MARCH8, MAX, MDGA1, MED1, MED12, MFSD11, MFSD5, MGAT1, MGLL, MID1IP1, MIER1, MKLN1, MLLT6, MLYCD, MPST, MRAS, MSRB3, MSX1, MT-ND5, MT1E, MT1F, MT1G, MT1H, MTF1, MTSS1L, MXI1, NAA16, NADK, NARG2, NCOA1, NFATC3, NFIB, NIPBL, NKX2-2, NOTCH1, NR2F2, NSL1, NUDT16L1, OMA1, OSBPL2, P2RX7, PAF1, PAK4, PAN2, PCBP1-AS1, PDGFRB, PEAK1, PEPD, PER1, PHF19, PHF2, PHF21A, PHLPP1, PHRF1, PI4K2B, PIGM, PLCB3, PLCG1, PLEKHB1, PLEKHM2, PLXNB1, PM20D2, PNISR, PNPT1, POLH, POLR2F, POLR3GL, POU3F2, POU3F3, PPM1K, PPP1R3G, PPP4R2, PPT2, PRELP, PRICKLE4, PRKD1, PRPF38B, PRX, PSMC3IP, PSPH, PTPN23, PTRHD1, RAB7L1, RAF1, RASA4, RBM28, RBM4B, RBPMS2, RENBP, REPIN1, RERE, RGCC, RGMA, RHOJ, RHOQ, RND2, RORA, RP11-226L15.5, RP11-349A22.5, RP11-45M22.1, RP11-552D4.1, RP11-632K20.7, RP11-644F5.11, RP11-742N3.1, RP4-773N10.4, RPL36AL, RPS21, RRP1B, RUFY1, S100A4, SAFB2, SALL3, SASH1, SELPLG, SERHL2, SESN3, SETD2, SETD8, SF3B2, SFRP2, SGPL1, SH3PXD2B, SHROOM1, SIGLEC8, SIRT1, SKI, SLC12A7, SLC25A48, SLC26A6, SLC38A2, SLC39A11, SLC41A1, SLC4A11, SLC6A12, SLC6A8, SLC6A9, SLC9A3R2, SMAD7, SMARCC1, SMC3, SMEK1, SMOC1, SMOX, SMTN, SNORD17, SNRNP48, SOCS4, SOGA1, SOX13, SRCAP, STAG1, STRA13, SUPT6H, SYNRG, SYVN1, TAF1C, TAF4, TAF8, TAL1, TBC1D20, TBC1D9B, TCF3, TCF7L1, TECR, TEX261, TGFB1I1, TLE4, TMCO3, TMEM161B-AS1, TMEM170A, TMEM184B, TMEM229A, TMEM79, TOB2, TPM3P9, TRIM65, TRIM8, TRPV3, TSC22D3, TSPAN11, TTC31, UBAC1, UBE2G2, UBIAD1, UBR5, UNC119B, USF2, VASH1, VAT1, VEGFB, VPS54, WAPAL, WDR55, WDR59, WDR6, WSCD1, WWC3, ZBTB12, ZBTB7B, ZC3H10, ZEB1-AS1, ZFP106, ZHX1, ZNF234, ZNF235, ZNF260, ZNF271, ZNF319, ZNF326, ZNF385A, ZNF438, ZNF599, ZNF646, ZNF672, ZNF853, ZNHIT6, ZSCAN21, ZSCAN29, ZSWIM7, |
| --- | --- |
| m122 | AADAT, ABCB8, AC007405.6, AC034220.3, AC093734.11, ACADSB, ACOT1, ACOT2, ACSL6, ACTG1, ADAM17, AGL, ALDH5A1, ANAPC10, ANKLE2, ANXA7, APEX1, ARHGEF4, ASAH1, ASH1L-AS1, ATG7, ATP5A1, ATP5S, BCKDK, BPGM, BRD1, C11orf1, C3orf17, C3orf18, C6orf130, C8orf40, C8orf42, CAPZB, CBWD1, CCBL2, CCDC14, CCDC53, CCDC71, CCDC93, CCNT2, CCPG1, CCZ1, CD27-AS1, CDC23, CHAMP1, CHCHD7, CHURC1, CLEC3B, CLK4, CNTNAP4, COG3, COX15, COX18, COX8A, CPPED1, CRY2, CRYZL1, CSTF2, CTH, CUTC, CYB5D2, DBNL, DCAF8, DCTD, DCTN6, DDB1, DDHD2, DDX19A, DDX42, DDX50, DFNA5, DHRS4-AS1, DIABLO, DNAJB5, DNAJC10, DOLK, DPH2, DPP8, DTD1, DUS4L, DYNC2LI1, EAF2, EDEM1, EEF1B2, EFHA1, EFHC1, EIF2AK1, EIF2B1, ELMOD2, ENOPH1, ERBB4, ERCC3, ERI2, EXOC1, EXOC3, FAM104A, FAM156A, FAM157C, FAM204A, FAM54B, FAM66A, FAM76A, FBN1, FBXO38, FECH, FH, FIGNL1, FIS1, FN3KRP, FOXRED2, GABBR1, GAPVD1, GCA, GFM1, GGCX, GIT2, GK5, GLT8D1, GOLGA8IP, GPM6A, GPR107, GTF2H3, GTPBP4, HARS2, HEY1, HLTF, HMGB1P5, HMGB3, HMGN2, HMGXB3, HNRNPD, HNRNPH2, HRASLS5, IK, ING3, INTS3, IQCB1, KBTBD3, KCNIP1, KHDRBS1, KIAA0907, KIAA1143, KIAA1609, KLHDC1, KLHL26, L3HYPDH, L3MBTL1, LCMT2, LDB1, LIG3, LINC00094, LINS, LOH12CR1, LRBA, LRRC41, LRRFIP2, LSM5, LUC7L2, LYRM2, MAGED2, MAN2B2, MBD4, MCCC1, MCEE, MCM6, MDM1, MED4, METTL10, METTL2A, MFSD8, MGEA5, MLH1, MPPE1, MTMR1, MTR, MXRA7, NAA25, NAA40, NAT8L, NDUFV1, NET1, NFS1, NIT1, NRAS, NSMAF, NUDT7, NUP133, NUP54, NUTF2, NXPH3, OBFC1, ODF2, ORC3, OXA1L, PAPD4, PCCB, PDAP1, PDCD6, PDE7B, PDHB, PDZD11, PECR, PER2, PEX1, PEX3, PEX7, PLA2G4C, PLBD2, PLRG1, PMS2, PNPLA4, POC5, POLR1A, POLR2J2, POLR3C, POLR3E, PPA2, PPCS, PPWD1, PRKAA1, PRKRIR, PRR13, PSMA3, PSMD5, PTCD3, PTPRA, PWP1, PYROXD1, QRICH1, QTRTD1, RAB5A, RAD17, RANBP10, RARS2, RBM3, RBMX, RECK, RERGL, RFT1, RINT1, RNF121, RNU1-6, RNU1-9, RP1-39G22.7, RP11-1407O15.2, RP11-220I1.1, RP11-231E4.4, RP11-261C10.3, RP11-304M2.2, RP11-313P13.4, RP11-33B1.1, RP11-399D2.1, RP11-6N17.4, RP11-823P9.1, RP13-104F24.1, RP5-1129J21.3, RRM1, RSPRY1, SAMM50, SAP18, SBDSP1, SC5DL, SCG3, SCRN3, SDHAP3, SEC23IP, SIGMAR1, SLC23A2, SLC25A3, SLC25A6, SLC35B3, SLC6A1, SLC6A6, SMN1, SMN2, SNRNP40, SNX4, SPATA24, SPCS1, SPOCK2, SRP54, SRSF2, SSBP1, SSR1, STK16, STX12, SUN1, SYNE1, SYT15, TAF1D, TAF9B, TATDN1, TCTN3, TFCP2, THAP9-AS1, THRAP3, TM2D1, TMEM161B, TMEM168, TMEM68, TMEM8B, TOP2B, TPCN2, TPM1, TPP2, TRAM1L1, TRAPPC4, TRIM9, TRMT11, TRPC1, TSC1, TSPAN7, TSR1, TSTD2, TTC13, TTC19, TTLL11, TUBA1A, TUBE1, TUBGCP5, TXNDC15, TYW1, UBE2V1, UBL5, UFSP2, UQCRC2, VPS13B, VPS39, VPS4B, WDR11, WDR61, WDR91, WI2-3658N16.1, XPNPEP1, ZBED5, ZDHHC4, ZDHHC6, ZMYM6, ZMYND12, ZNF10, ZNF136, ZNF138, ZNF140, ZNF197, ZNF211, ZNF222, ZNF226, ZNF26, ZNF266, ZNF277, ZNF280D, ZNF333, ZNF334, ZNF354B, ZNF37BP, ZNF418, ZNF432, ZNF433, ZNF496, ZNF498, ZNF514, ZNF626, ZNF627, ZNF696, ZNF7, ZNF706, ZNF717, ZNF75D, ZNF841, ZRANB2, |
