# Supplementary material for: Seasonal plasticity of cognition and related biological measures in adults with and without Alzheimer disease: Analysis of multiple cohorts
Source: PLoS Med. 2018 Sep 4;15(9):e1002647. doi: 10.1371/journal.pmed.1002647 (PMC6122787; doi:10.1371/journal.pmed.1002647)
Supplement: S5 Table — (DOCX) [file pmed.1002647.s013.docx]

**S5 Table: Impact of Season on the Expression of Cognition-Associated Molecular Systems – Consideration of Potential Confounders**

| **Module** | **Model** | **Covariates** | **Acrophase (radians)** | **Amplitude** | **P-value for Rhythmicity** | **Estimated Effect on Cognition (per 1SD difference)** | **P-Value for Association with Cognition** |
| --- | --- | --- | --- | --- | --- | --- | --- |
| m6 | A | Base Model | 0·81 | 0·234 | 0·001 | -0·17 | <0·001 |
| m6 | B | A+depression | 0·81 | 0·234 | 0·001 | -0·16 | <0·001 |
| m6 | C | B+neuropathology | 0·95 | 0·214 | 0·003 | -0·09 | 0·009 |
| m13 | A | Base Model | 3·37 | 0·234 | 0·001 | 0·19 | <0·001 |
| m13 | B | A+depression | 3·36 | 0·233 | 0·001 | 0·17 | <0·001 |
| m13 | C | B+neuropathology | 3·42 | 0·197 | 0·007 | 0·1 | 0·012 |
| m109 | A | Base Model | 0·24 | 0·221 | 0·002 | -0·23 | <0·001 |
| m109 | B | A+depression | 0·23 | 0·221 | 0·002 | -0·22 | <0·001 |
| m109 | C | B+neuropathology | 0·32 | 0·182 | 0·013 | -0·14 | 0·021 |
| m122 | A | Base Model | 3·81 | 0·236 | 0·001 | 0·17 | <0·001 |
| m122 | B | A+depression | 3·8 | 0·237 | 0·001 | 0·16 | <0·001 |
| m122 | C | B+neuropathology | 3·95 | 0·207 | 0·004 | 0·08 | 0·008 |
