# Supplementary material for: Seasonal plasticity of cognition and related biological measures in adults with and without Alzheimer disease: Analysis of multiple cohorts
Source: PLoS Med. 2018 Sep 4;15(9):e1002647. doi: 10.1371/journal.pmed.1002647 (PMC6122787; doi:10.1371/journal.pmed.1002647)
Supplement: S6 Table — Columns indicate p-values for association with module membership for each set of transcription factor binding sites as described in the text. **Significant after correcting for multiple comparisons. Transcription factors labeled with “++” were themselves found to have seasonally rhythmic transcript expression in a recent study [8]. (DOCX) [file pmed.1002647.s014.docx]

**S6 Table: Transcription Factor Binding Sites Linked to Cognition-Associated Molecular Modules.** Columns indicate p-values for association with module membership for each set of transcription factor binding sites as described in the text. ** indicates significant after correcting for multiple comparisons. Transcription factors labeled with ++ were themselves found to have seasonally rhythmic transcript expression in a recent study [8].

|  | **p-value for association with module membership** | | | |
| --- | --- | --- | --- | --- |
|  | **m6** | **m13** | **m109** | **m122** |
| ARID3A | 2·0E-02 | 1·9E-01 | 2·3E-01 | 5·5E-02 |
| ATF1 | 4·2E-02 | 1·9e-05** | 4·1E-01 | 1·7E-01 |
| ATF2 | 4·5e-05** | 4·4E-01 | 8·7E-01 | 1·3E-01 |
| ATF3 | 7·4E-04 | 3·7E-01 | 4·9E-03 | 1·8E-01 |
| BACH1 | 6·7E-02 | 4·3E-04 | 2·1E-01 | 2·0E-01 |
| BATF | 6·6E-01 | 6·2E-01 | 2·5E-02 | 9·1E-03 |
| BCL11A++ | 4·7E-02 | 4·5E-03 | 2·8e-04** | 2·4E-01 |
| BCL3 | 6·5E-01 | 5·9E-01 | 1·8E-01 | 1·6e-06** |
| BCLAF1 | 4·7E-01 | 1·2E-02 | 2·0E-01 | 1·1E-03 |
| BDP1 | 7·1E-01 | 3·3e-06** | 9·6E-01 | 2·7E-02 |
| BHLHE40 | 4·2E-01 | 7·7E-01 | 2·4E-01 | 7·2E-01 |
| BRCA1 | 2·6E-01 | 1·7E-02 | 2·4E-02 | 3·8E-01 |
| BRF1++ | 9·3E-01 | 9·3E-01 | 9·7E-01 | 8·0E-01 |
| BRF2 | 1·0E-01 | 2·0E-01 | 2·7E-02 | 4·4E-02 |
| CBX3 | 4·0E-01 | 8·1E-02 | 2·5E-03 | 1·7E-03 |
| CCNT2++ | 3·6E-01 | 9·6E-01 | 1·0E-01 | 6·0E-01 |
| CEBPB | 1·0E-02 | 8·4E-02 | 1·6E-02 | 8·7E-04 |
| CEBPD | 2·4E-01 | 1·7E-01 | 7·8E-01 | 2·2E-01 |
| CHD1 | 2·1E-01 | 2·7e-07** | 1·3E-01 | 3·4E-04 |
| CHD2 | 1·0E-01 | 8·3E-01 | 8·8E-02 | 2·6E-01 |
| CREB1 | 1·5E-01 | 2·0e-04** | 5·9E-01 | 6·0E-01 |
| CTBP2 | 2·7E-01 | 8·4E-02 | 4·6E-02 | 5·2E-02 |
| CTCF++ | 2·2E-01 | 1·3e-04** | 1·7E-01 | 2·4E-02 |
| CTCFL | 6·1E-02 | 9·0E-02 | 4·8E-01 | 2·6E-01 |
| E2F1 | 3·8E-02 | 8·3E-01 | 1·5E-01 | 6·2E-01 |
| E2F4 | 4·7E-01 | 8·3E-02 | 1·8E-01 | 6·4E-02 |
| E2F6 | 3·6E-01 | 2·2E-01 | 9·6E-01 | 1·0E+00 |
| EBF1 | 1·7E-01 | 6·3E-03 | 1·3E-01 | 1·8E-02 |
| EGR1++ | 9·8E-01 | 5·3E-02 | 7·4e-06** | 1·4E-02 |
| ELF1 | 5·5E-01 | 8·7E-02 | 4·3E-03 | 3·9E-01 |
| ELK1 | 2·4e-04** | 8·2E-01 | 7·6E-01 | 5·7E-01 |
| ELK4++ | 2·9E-01 | 5·5E-01 | 5·4E-01 | 2·7E-01 |
| EP300 | 6·9E-01 | 4·5E-01 | 5·8E-01 | 9·1E-01 |
| ESR1 | 1·3E-01 | 3·9E-01 | 2·4E-02 | 9·7e-05** |
| ESRRA | 2·0E-01 | 2·9E-02 | 9·0E-01 | 4·5E-02 |
| ETS1 | 6·1E-02 | 4·2E-01 | 8·9E-01 | 6·3E-01 |
| EZH2 | 1·7E-03 | 1·8E-01 | 2·3E-02 | 4·4E-01 |
| FAM48A | 4·4E-02 | 5·5E-02 | 8·5E-01 | 6·2E-01 |
| FOS | 2·1e-04** | 1·8E-02 | 1·0E-02 | 6·6E-02 |
| FOSL1 | 3·2E-02 | 1·2E-01 | 1·1E-02 | 3·9E-03 |
| FOSL2 | 1·1E-01 | 7·4E-01 | 5·7E-01 | 6·2E-01 |
| FOXA1 | 3·0E-03 | 3·7E-01 | 7·7E-01 | 1·8E-01 |
| FOXA2 | 1·6E-01 | 7·9E-01 | 7·3E-01 | 4·8E-01 |
| FOXM1 | 1·5e-06** | 2·2E-01 | 7·0E-01 | 1·7E-01 |
| FOXP2 | 4·5E-01 | 3·0E-01 | 9·6E-01 | 1·8E-03 |
| GABPA++ | 3·3E-01 | 4·1E-02 | 3·8E-01 | 2·2E-01 |
| GATA1 | 3·1E-03 | 9·3E-03 | 9·5E-02 | 8·4E-01 |
| GATA2 | 6·2E-03 | 6·7E-02 | 3·6E-01 | 1·7E-01 |
| GATA3 | 2·9E-03 | 6·3E-01 | 6·3E-01 | 2·9E-01 |
| GRp20 | 3·1e-07** | 6·9E-02 | 3·1E-01 | 6·9E-01 |
| GTF2B | 1·8e-04** | 5·1E-01 | 3·3E-02 | 8·2E-04 |
| GTF2F1 | 2·6E-01 | 8·3E-01 | 8·1E-01 | 1·5E-01 |
| GTF3C2 | 1·5E-01 | 6·1E-01 | 5·7E-02 | 1·3E-01 |
| HDAC1 | 6·6E-01 | 9·0e-06** | 9·7E-01 | 4·2E-01 |
| HDAC2 | 6·6E-01 | 4·1E-01 | 3·4E-01 | 1·4E-03 |
| HDAC6 | 9·7E-01 | 8·8E-01 | 1·4E-01 | 8·8E-03 |
| HDAC8 | 1·9E-01 | 1·9E-01 | 1·4E-02 | 2·0E-02 |
| HMGN3 | 6·7E-01 | 2·2E-03 | 1·5E-03 | 2·0E-03 |
| HNF4A | 9·1E-01 | 1·9e-04** | 1·0E-01 | 7·3E-01 |
| HNF4G | 7·5E-02 | 4·0E-01 | 3·0E-02 | 3·1E-01 |
| HSF1 | 1·9E-01 | 1·4E-01 | 2·1E-02 | 3·7E-01 |
| IKZF1 | 3·4E-01 | 3·0E-02 | 1·1E-02 | 8·4E-02 |
| IRF1 | 5·4E-02 | 5·1E-01 | 6·8E-01 | 9·6E-01 |
| IRF3++ | 4·3E-04 | 4·8E-01 | 2·4E-02 | 4·8E-01 |
| IRF4 | 7·6E-01 | 3·9E-02 | 1·7E-01 | 1·5E-03 |
| JUN | 8·9E-01 | 9·4E-01 | 2·2E-03 | 1·7E-01 |
| JUNB | 9·4E-01 | 5·7E-01 | 7·6E-04 | 2·4E-01 |
| JUND | 7·3E-03 | 1·4E-01 | 5·8E-01 | 9·5E-01 |
| KAP1 | 2·1e-04** | 6·4E-03 | 2·5E-03 | 1·8E-03 |
| KDM5A | 8·7E-02 | 1·1E-01 | 1·4E-03 | 7·9E-01 |
| KDM5B | 6·0E-02 | 3·2E-02 | 9·4E-01 | 7·4E-03 |
| MAFF | 4·7E-01 | 8·2E-01 | 1·2E-02 | 3·1E-01 |
| MAFK | 8·1E-03 | 9·3E-01 | 1·5E-01 | 1·1E-01 |
| MAX++ | 1·2E-03 | 2·4E-01 | 1·4E-03 | 5·3E-02 |
| MAZ | 5·1E-02 | 3·4E-01 | 6·1E-01 | 1·3e-04** |
| MBD4++ | 3·3E-01 | 7·1E-01 | 1·8E-01 | 6·7E-01 |
| MEF2A | 1·8e-05** | 2·7E-01 | 6·2E-02 | 3·6E-01 |
| MEF2C++ | 7·9E-01 | 2·1e-04** | 4·9E-01 | 1·2E-01 |
| MTA3 | 2·7E-01 | 8·3E-01 | 8·1E-01 | 1·0E-03 |
| MXI1 | 1·3E-02 | 3·9E-04 | 3·2E-02 | 1·7E-02 |
| MYBL2 | 2·8E-01 | 3·6E-02 | 8·4E-01 | 2·6E-01 |
| MYC | 1·5E-01 | 4·2E-01 | 1·1E-01 | 7·5E-01 |
| NANOG | 3·7E-01 | 2·0E-01 | 1·5e-06** | 1·9E-01 |
| NFATC1 | 7·2e-08** | 7·6E-01 | 4·2E-01 | 1·4E-03 |
| NFE2 | 6·2E-01 | 8·8E-03 | 3·0E-02 | 2·2E-01 |
| NFIC | 2·1E-01 | 3·3E-01 | 8·5E-02 | 1·4e-07** |
| NFYA++ | 1·1E-02 | 1·2E-03 | 6·3E-02 | 1·1E-01 |
| NFYB | 2·1E-02 | 8·5E-01 | 6·5E-02 | 9·1E-01 |
| NR2C2 | 3·3E-01 | 1·1E-01 | 3·2E-01 | 1·0E-03 |
| NR2F2 | 2·5E-01 | 7·8E-03 | 5·9E-01 | 2·6E-01 |
| NR3C1 | 5·8E-01 | 8·0E-04 | 5·0E-01 | 3·1E-01 |
| NRF1 | 4·9E-02 | 3·2e-07** | 1·2E-01 | 7·3E-01 |
| PAX5 | 8·3E-01 | 1·0e-04** | 6·2E-01 | 1·8E-01 |
| PBX3 | 3·7E-01 | 2·1E-01 | 4·1E-01 | 9·9E-01 |
| PHF8 | 2·8E-01 | 9·8E-01 | 5·7E-01 | 1·4E-03 |
| PML | 8·7E-03 | 1·7E-01 | 1·8E-03 | 5·3E-04 |
| POLR2A | 1·2e-05** | 1·1E-02 | 4·1E-02 | 1·9E-02 |
| POLR3G | 9·8E-01 | 2·4E-01 | 9·7E-01 | 4·5E-01 |
| POU2F2 | 2·4E-01 | 1·3E-02 | 2·9E-01 | 8·4E-02 |
| POU5F1 | 2·9E-02 | 2·7E-02 | 1·7E-02 | 3·5E-01 |
| PPARGC1A | 3·0E-01 | 9·4e-07** | 7·3E-01 | 1·5E-01 |
| PRDM1 | 4·2E-02 | 1·4E-01 | 3·0E-02 | 6·1E-02 |
| RAD21 | 6·2E-02 | 8·6E-01 | 9·8E-01 | 3·2E-01 |
| RBBP5 | 5·5E-01 | 5·3E-01 | 9·7E-02 | 1·2E-01 |
| RCOR1 | 4·7E-01 | 4·7E-01 | 4·2E-01 | 8·8E-02 |
| RDBP | 1·7E-01 | 4·7E-04 | 1·6E-01 | 1·5E-01 |
| RELA | 2·5e-04** | 3·4E-02 | 4·4E-03 | 5·4E-01 |
| REST | 1·3E-02 | 2·3E-01 | 2·7E-01 | 3·6E-01 |
| RFX5 | 4·8E-01 | 7·7E-01 | 4·0E-01 | 2·6E-02 |
| RPC155 | 5·1E-01 | 3·4E-01 | 2·4E-01 | 4·4E-01 |
| RUNX3 | 4·4E-01 | 9·8E-02 | 9·8E-01 | 3·4E-01 |
| RXRA | 8·8E-01 | 2·1E-02 | 6·3E-01 | 5·7E-01 |
| SAP30++ | 9·4E-03 | 5·1E-01 | 1·3E-01 | 1·2E-01 |
| SETDB1 | 2·0e-04** | 6·2E-01 | 1·9e-05** | 4·9E-01 |
| SIN3A | 1·3E-01 | 9·9E-01 | 3·7E-03 | 7·1E-01 |
| SIN3AK20 | 4·2E-01 | 4·9E-01 | 1·3E-01 | 7·6E-03 |
| SIRT6 | 9·3E-02 | 1·0e-04** | 8·0E-01 | 1·8E-01 |
| SIX5 | 1·2E-01 | 1·5E-01 | 3·8E-03 | 2·0E-02 |
| SMARCA4 | 5·3E-01 | 1·9E-01 | 4·2E-01 | 8·1E-01 |
| SMARCB1 | 6·3E-01 | 2·6E-02 | 3·6E-01 | 9·5E-03 |
| SMARCC1 | 5·9E-02 | 4·4E-02 | 6·0E-01 | 5·2E-01 |
| SMARCC2++ | 3·9E-01 | 5·0E-01 | 8·1E-01 | 4·0E-01 |
| SMC3 | 8·4E-02 | 2·3E-02 | 7·2E-02 | 4·7E-01 |
| SP1 | 3·8E-01 | 8·6E-01 | 3·5E-01 | 9·2E-01 |
| SP2 | 1·5E-01 | 1·6E-01 | 7·4E-01 | 3·3E-03 |
| SP4 | 3·0E-02 | 1·6E-01 | 4·4E-01 | 7·5E-01 |
| SPI1 | 6·0E-01 | 4·9E-02 | 6·7E-01 | 6·9E-02 |
| SREBP1 | 7·2E-04 | 8·6E-01 | 5·6E-02 | 2·6e-05** |
| SRF | 3·5E-02 | 4·2E-01 | 1·4E-01 | 3·7E-02 |
| STAT1 | 2·6E-01 | 5·3E-02 | 4·7E-02 | 5·9E-01 |
| STAT2 | 2·9E-01 | 6·4e-08** | 9·5E-01 | 1·4E-01 |
| STAT3 | 2·3E-02 | 1·5E-02 | 4·0E-04 | 4·5E-01 |
| STAT5A | 3·1E-01 | 5·5E-02 | 6·5E-01 | 3·1E-02 |
| SUZ12 | 1·6E-03 | 4·4E-02 | 5·9E-01 | 3·2E-03 |
| TAF1 | 2·8E-03 | 3·8E-01 | 5·7E-01 | 2·5E-01 |
| TAF7 | 9·8E-02 | 2·3E-02 | 4·3E-01 | 6·7E-01 |
| TAL1 | 7·2E-01 | 4·4E-03 | 3·4E-01 | 7·6E-01 |
| TBL1XR1 | 4·8E-02 | 3·6E-01 | 6·5E-01 | 2·1E-01 |
| TBP | 9·2E-01 | 6·1E-01 | 6·3E-01 | 9·8E-01 |
| TCF12 | 2·8E-03 | 7·5E-02 | 3·7E-01 | 9·2E-03 |
| TCF3 | 2·8E-01 | 3·2E-01 | 1·5E-03 | 1·9E-01 |
| TCF7L2 | 1·5E-01 | 4·1E-01 | 2·5E-01 | 4·9E-01 |
| TEAD4 | 2·4E-01 | 6·2E-01 | 1·4E-02 | 2·9e-05** |
| TFAP2A | 4·9E-01 | 9·0E-02 | 8·3E-04 | 4·6E-01 |
| TFAP2C | 3·0E-02 | 2·4E-01 | 2·8E-02 | 7·6E-01 |
| THAP1++ | 9·1E-01 | 9·8E-01 | 9·9e-07** | 7·1E-01 |
| TRIM28 | 1·0e-06** | 7·8E-01 | 1·5E-01 | 6·5E-01 |
| UBTF | 5·0E-01 | 8·3E-01 | 4·8E-01 | 5·6E-01 |
| USF1 | 4·9E-01 | 9·9E-01 | 2·6E-02 | 3·8E-01 |
| USF2 | 4·2E-01 | 2·6E-01 | 6·1E-01 | 5·9E-01 |
| WRNIP1++ | 3·0E-01 | 6·1E-01 | 3·3E-02 | 1·3E-01 |
| YY1 | 4·6E-01 | 5·1E-01 | 4·8E-02 | 4·1E-01 |
| ZBTB33 | 7·6e-06** | 9·6E-01 | 1·4E-01 | 5·5E-01 |
| ZBTB7A | 6·1E-04 | 2·9E-03 | 1·1e-04** | 4·2e-08** |
| ZEB1 | 2·5e-06** | 2·3E-02 | 3·3E-02 | 6·6E-01 |
| ZKSCAN1++ | 1·6E-02 | 6·8E-01 | 1·9E-02 | 3·2E-01 |
| ZNF143 | 5·7E-02 | 7·8E-01 | 2·0E-01 | 1·5e-07** |
| ZNF217++ | 1·5E-01 | 7·9E-01 | 2·3E-02 | 1·7E-02 |
| ZNF263 | 6·5E-01 | 2·9E-01 | 4·7E-02 | 5·2E-01 |
| ZNF274 | 2·1E-01 | 8·2E-04 | 1·8E-02 | 1·7e-05** |
| ZZZ3 | 3·0E-01 | 5·6E-01 | 4·0E-01 | 7·3E-02 |
